# GARP is a key molecule for mesenchymal stromal cell responses to TGF‐β and fundamental to control mitochondrial ROS levels

**DOI:** 10.1002/sctm.19-0372

**Published:** 2020-02-19

**Authors:** Ana Belén Carrillo‐Gálvez, Sheyla Gálvez‐Peisl, Juan Elías González‐Correa, Marina de Haro‐Carrillo, Verónica Ayllón, Pedro Carmona‐Sáez, Verónica Ramos‐Mejía, Pablo Galindo‐Moreno, Francisca E. Cara, Sergio Granados‐Principal, Pilar Muñoz, Francisco Martin, Per Anderson

**Affiliations:** ^1^ Centre for Genomics and Oncological Research (GENYO), Pfizer/University of Granada/Andalucian Regional Government Granada Spain; ^2^ Department of Oral Surgery and Implant Dentistry School of Dentistry, University of Granada Granada Spain; ^3^ UGC de Oncología Médica, Hospital Universitario de Jaén Jaén Spain; ^4^ Servicio de Análisis Clínicos e Inmunología, UGC Laboratorio Clínico Hospital Universitario Virgen de las Nieves Granada Spain; ^5^ Biosanitary Institute of Granada (ibs.Granada), University of Granada Spain

**Keywords:** DNA damage, mesenchymal stromal cells (MSCs), proliferation, ROS, TGF‐β

## Abstract

Multipotent mesenchymal stromal cells (MSCs) have emerged as a promising cell therapy in regenerative medicine and for autoimmune/inflammatory diseases. However, a main hurdle for MSCs‐based therapies is the loss of their proliferative potential in vitro. Here we report that glycoprotein A repetitions predominant (GARP) is required for the proliferation and survival of adipose‐derived MSCs (ASCs) via its regulation of transforming growth factor‐β (TGF‐β) activation. Silencing of GARP in human ASCs increased their activation of TGF‐β which augmented the levels of mitochondrial reactive oxygen species (mtROS), resulting in DNA damage, a block in proliferation and apoptosis. Inhibition of TGF‐β signaling reduced the levels of mtROS and DNA damage and restored the ability of GARP^−/low^ASCs to proliferate. In contrast, overexpression of GARP in ASCs increased their proliferative capacity and rendered them more resistant to etoposide‐induced DNA damage and apoptosis, in a TGF‐β‐dependent manner. In summary, our data show that the presence or absence of GARP on ASCs gives rise to distinct TGF‐β responses with diametrically opposing effects on ASC proliferation and survival.

## Graphical abstract

Silencing of glycoprotein A repetitions predominant (GARP) in adipose‐derived mesenchymal stromal cells (ASCs) increases the activation of transforming growth factor‐β (TGF‐β), resulting in mitochondrial reactive oxygen species‐dependent DNA damage, inhibition of proliferation, and apoptosis. In contrast, overexpression of GARP induces a TGF‐β response characterized by an increased resistance to DNA damage, increased proliferation, and a reduction in apoptosis. We show that GARP is critical in controlling TGF‐β activation/signaling in ASCs during in vitro expansion.

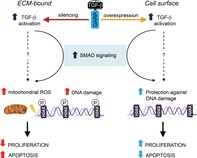

**Significance statement**

The expansion of multipotent mesenchymal stromal cells (MSCs) in vitro is associated with a decrease in their proliferative and therapeutic capacity making basic research on factors regulating MSC proliferation of fundamental importance for their successful translation into clinical applications. It is shown that glycoprotein A repetitions predominant (GARP) is critical for the proliferation and survival of adipose‐derived MSCs (ASCs) in vitro. GARP prevents an aberrant transforming growth factor‐β (TGF‐β) response in ASCs, characterized by oxidative DNA damage and cell death, while inducing a productive TGF‐β response that increases their proliferation and resistance to DNA damage. The data highlight the importance of GARP in controlling TGF‐β activation/signaling in ASCs during in vitro expansion.

## 1 INTRODUCTION

Multipotent mesenchymal stromal cells (MSCs) are non‐hematopoietic, perivascular cells that can be found in virtually all organs and tissues in the body. Isolated MSCs have been defined as plastic adherent, CD73^+^CD90^+^CD105^+^CD11b^−^CD31^−^CD45^−^ cells that can differentiate into adipocytes, chondroblasts, and osteoblasts in vitro^1^. in vitro expanded MSCs have been shown to promote tissue repair and inhibit inflammatory/autoimmune responses in preclinical^2^, ^3^, ^4^ and human clinical trials.^5^, ^6^ However, despite promising results and the observed safety, the beneficial effects of MSCs in many clinical trials have been disappointing.^7^

A major hurdle for MSC‐based therapies is the need to expand MSCs in vitro due to the large number of cells needed/patient (1‐10 × 10^6^ cells/kg)^8^ and the low frequency of MSCs in tissues (from 0.3% to 4% in adipose tissue^9^ to 0.001%‐0.01% in the bone marrow^10^). The expansion of MSCs is associated with several problems including the loss of homing capacity,^11^ onset of cellular senescence,^12^ decrease in differentiation capacity,^13^ and susceptibility to genomic instability and malignant transformation,^14^, ^15^ limiting the utility of MSCs as a cell‐based therapy. Another problem is the low survival and engraftment of injected cells in vivo,^16^ and it has been estimated that up to 99% of the injected MSCs will die shortly after administration.^17^, ^18^ Several of the above obstacles can be circumvented using the MSC‐derived secretome which has shown beneficial effects in inflammatory/autoimmune diseases and in regenerative medicine. However, the therapeutic efficacy of the secretome depends on the functional properties of the MSCs from which it was obtained. Thus, research on optimizing the culture conditions of MSCs is an important endeavor when developing therapies using either MSCs or the MSC‐derived secretome.^19^, ^20^ Understanding the factors governing MSC expansion in vitro and the development of an MSC product with high‐expansion capacity and increased genomic stability would most likely increase the success of MSCs in cell therapy.^21^

Glycoprotein A repetitions predominant (GARP), also known as LRRC32, is a type I transmembrane protein which belongs to the leucine‐rich repeat family of proteins which encompasses a large number of intracellular and extracellular proteins with distinct functions in neural development, innate immunity, and inflammation, including toll‐like receptors and cell adhesion molecules.^22^, ^23^ GARP binds and activates latency‐associated peptide (LAP)/transforming growth factor (TGF)‐β1 complexes on the surface of regulatory T cells (Tregs)^24^, ^25^, ^26^ and B‐cells,^27^, ^28^ supporting their immunosuppressive capacity and isotype switching to IgA production, respectively. We have previously shown that GARP binds LAP/TGF‐β1 to the surface of murine ASCs, regulating the activation of TGF‐β and modulating their immunomodulatory capacity. Interestingly, silencing of GARP in ASCs significantly decreased their proliferative capacity, but the mechanisms remain unknown.^29^ GARP has been shown to both inhibit^30^ and promote^31^, ^32^ T‐cell proliferation. Also, GARP expression in HeLa cells correlated positively with their proliferative capacity, whereas silencing of GARP in the NmuMG breast cancer cell line did not affect their proliferation,^32^, ^33^ suggesting that the impact of GARP on proliferation depends on cell type. GARP can also regulate cell proliferation/survival via its activation of TGF‐β. TGF‐β1 has been found to both promote^34^, ^35^ and inhibit^36^, ^37^ the proliferation of MSCs in vitro, and some studies have suggested that TGF‐β1 can exert a biphasic effect on MSC proliferation, where low concentrations (≤0.25 ng/mL) increase proliferation while higher concentrations (≥1 ng/mL) inhibit proliferation.^38^, ^39^

The aim of the current study was to investigate the mechanism behind the block in proliferation of GARP‐silenced human ASCs and analyze the effect of GARP‐overexpression on ASC proliferation and survival. We found that silencing of GARP in ASCs (GARP^−/low^ASCs) increased the activation of TGF‐β and induced an increase in basal mitochondrial reactive oxygen species (mtROS) and γ‐H2AX levels, indicating higher levels of DNA damage. Blocking TGF‐β signaling in GARP^−/low^ASCs reduced the mtROS levels and DNA damage and significantly reverted their block in proliferation, suggesting that in the absence of GARP, the activation of TGF‐β by ASCs induces a deleterious response. In contrast, GARP‐overexpressing ASCs exhibited an increased proliferative capacity and an increased resistance to DNA damage and apoptosis, through a TGF‐β‐dependent mechanism. Our data indicate that GARP is required to regulate TGF‐β activation/signaling, allowing ASCs to grow in stress conditions, such as in vitro culture.

## 2 MATERIALS AND METHODS

### 2.1 Culture of ASCs

Passage 3 ASCs were obtained from the Biobanco del Sistema Sanitario Público de Andalucía (Parque Tecnológico Ciencias de la Salud, Centro de investigación Biomédica, Granada, Spain). All experiments using human samples were performed according to the Institutional Guidelines and approved by the Ethics Committee of the H.U. Virgen de Macarena. The ASCs were cultured in complete advanced DMEM (supplemented with 10% FCS—Invitrogen, Carlsbad, California), Glutamax and 100 U/mL penicillin/streptomycin (both from GIBCO, Life Technologies, California) at 21% O_2_/5% CO_2_ at 37°C. All MSCs used in our studies were CD45^−^CD73^+^CD90^+^CD105^+^ (data not shown).

### 2.2 Production of lentiviral vectors and transduction of ASCs

In order to silence GARP in ASCs, lentiviral vectors (LVs) encoding two human GARP‐specific shRNAs (MISSION shRNA plasmid DNAs, RefSeq: SHCLND‐NM_005512; Sigma‐Aldrich, Missouri) were used, referred to in the manuscript as LV#18 (TRCN0000005218) and LV#19 (TRCN0000005219). A MISSION pLKO.1‐puro non‐mammalian shRNA plasmid (Sigma‐Aldrich) was used as control. The human codon‐optimized GARP cDNA was synthesized by Genscript (Genscript, Piscataway, New Jersey) and subcloned into the LV‐CEWP plasmid,^40^ under the control of the CMV promoter, exchanging the eGFP and creating LV‐GARP. LVs were produced by co‐transfecting 293 T cells with (i) vector LV‐shRNA/LV‐GARP/LV‐CEWP plasmid, (ii) packaging plasmid pCMVΔR8.91, and (iii) envelope plasmid pMD.G as previously described.^41^ The LVs were subsequently concentrated (×30) using centrifugal filter devices (Amicon Ultra‐15, 100 kD, Merck). Genetic modifications of ASCs with LVs and the determination of vector copy number/transduced ASC were performed as previously described.^42^ For the different LV‐transduced cells, the following primers were used: LV‐backbone FW: 5′‐GACGGTACAGGCCAGACAA‐3′, LV‐backbone RV: 5′‐ TGGTGCAAATGAGTTTTCCA‐3′. We used an MOI~10 to obtain 2‐3 LV integrations/cell.

To overexpress GARP, referred to in the manuscript as LV‐GARP, 0.7 × 10^6^ ASCs (passage 3‐6) were mixed with the concentrated viruses (LV‐GARP) and subsequently seeded in 6‐well plates and maintained at 37°C. After 5 hours, the media were replaced by fresh medium. The next day, cells were transduced again with the concentrated virus and after 5 hours were expanded in T75 flasks at 21% O_2_/5% CO_2_ at 37°C.

To rescue GARP expression in GARP‐silenced cells, referred to in the manuscript as LV#19 + LV‐GARP, 0.7 × 10^6^ ASCs (passage 3‐6) were mixed with concentrated LV#19 and LV‐GARP for 5 hours. The LV‐containing media were then replaced by fresh medium. The next day, cells were transduced again with concentrated LV‐GARP for 5 hours and were subsequently expanded in T75 flasks at 21% O_2_/5% CO_2_ at 37°C. GARP expression was analyzed by flow cytometry 4 days after transduction.

### 2.3 Detection of surface GARP and sorting of GARP^++^ASCs

ASCs were stained with an anti‐human GARP antibody (GARP‐eFluor660) from eBioscience (San Diego, California). A rat IgG2a kappa‐eFluor660 Isotype control (eBioscience) was used in order to determine the background staining, and dead cells were excluded using 7AAD (eBioscience). Cells were acquired on a FACS Canto II flow cytometer and analyzed using the FACS Diva Software (BD Bioscience). After GARP staining of LV‐GARP ASCs, the nontransduced (NT(S) and GARP‐overexpressing (GARP^++^) ASCs were separated using a FACS Aria flow cytometer (BD Bioscience).

### 2.4 Analysis of ASC proliferation

To analyze the effect of GARP on ASC proliferation, the xCelligence real‐time cell analyzer system from Roche (Roche Applied System, Penzberg, Germany) was used. In brief, 1000 cells/well of NT, LV‐CTRL, LV#18, LV#19, or LV#19 + LV‐GARP ASCs were added to 16‐well E‐plates (Roche Applied System) as previously described.^43^ The E‐plates were then placed on the device station in the incubator (21% O_2_/5% CO_2_ at 37°C) for the continuous recording of impedance, as reflected by cell index. In order to understand the mechanisms behind the inhibition of proliferation in GARP^−/low^ASCs, NT, LV‐CTRL, LV#18, and LV#19 ASCs were treated with SB431542 (10 μM), N‐acetyl cystein (NAC, 1 mM), apocynin (5 mM), mitoTEMPO (25 μM) (all inhibitors are from Sigma‐Aldrich) or anti‐TGF‐β1/2/3 Ab (11D1, R&D Systems, Minneapolis, Minnesota), starting 1 day after GARP silencing. Cells were harvested 3 days later and added to 16‐well E‐plates (1000 cells/well), and the proliferation was followed as described above. Fresh media with or without inhibitors were added every 3 days. To analyze the proliferation of NT(S) and GARP^++^ASCs, cells were plated at low density (50 000 cells/well) in 6‐well plates. When the cell cultures reached an 80‐90% of confluence, cells were harvested, counted, and replated at the same concentration. The proliferation was followed for 3‐4 weeks.

### 2.5 7AAD/Annexin V Staining

To analyze apoptosis, 50 000 cells/well of NT, LV‐CTRL, LV#18, LV#19, LV#19 + LV‐GARP, or LV‐GARP ASCs were added to 12‐well plates, 4 days after cell transduction. Apoptosis was analyzed 5 and 11 days later using the 7AAD/PE Annexin V Apoptosis Kit I (BD Biosciences) according to the manufacturer's instructions. The frequency of Annexin V‐positive cells was measured by flow cytometry. To analyze apoptosis in NT(S) and GARP^++^ASCs, 50 000 cells/well were plated in 12‐well plates. The next day, 25 μM of etoposide (Sigma‐Aldrich) was added to the cells, and 2 days later the percentage of Annexin V‐positive cells was analyzed by flow cytometry. In some experiments, SB431542 was added to the cells 5 hours before the addition of etoposide.

### 2.6 Gene expression profiling and data analysis

In order to analyze the effects of GARP silencing on the gene expression in ASCs, total RNA (500 ng), isolated from three independent biological replicates of NT, LV‐CTRL, LV#18, and LV#19 ASCs 6 days after transduction, was amplified using the Illumina TotalPrep RNA Amplification Kit (Ambion, Austin, Texas), reverse‐transcribed into first‐ and second‐strand cDNA and cRNA labeled with biotin was generated according to the manufacturer's instructions. The cRNA was hybridized overnight to the Human HT‐12 V4.0 BeadChip arrays (Illumina). Beadchips were washed, stained with dye‐labeled streptavidin, and scanned with the Illumina IScan. Raw data were exported from Illumina GenomeStudio and processed in R using negative control probes for background correction and quantile normalization.^44^ Probes with detection *P*‐values <.05 in at least two replicates were discharged, and expression values of the remaining probes corresponding to the same gene were aggregated by the median value. Differential expression analysis was carried out using linear models implemented in the limma R package.^45^ Genes with an FDR‐adjusted *P*‐value <.05 and absolute fold‐change >0.5 were selected as significantly differentially expressed. Analysis of gene functions and canonical pathways was performed using the Ingenuity Pathway Analysis (IPA) software (Ingenuity Systems Inc., Redwood City, California).

### 2.7 5‐bromo‐2′‐deoxyuridine incorporation assay

To study the proliferation of NT, LV‐CTRL, LV#18, and LV#19 ASCs, cells were pulsed with 5‐bromo‐2′‐deoxyuridine (BrdU) (10 μM for 3 hours), 5 days after transduction. Cells were harvested after the BrdU pulse and stained for BrdU using the BD Pharmingen BrdU Flow kit (BD Biosciences) following the manufacturer's instructions. The cell cycle of ASCs was studied by pulsing NT, LV‐CTRL, LV#18, and LV#19 ASCs with BrdU (10 μM for 3 hours), 24 hours after transduction and harvesting the cells 3 days later. Cells were stained for BrdU and 7AAD using the BD Pharmingen BrdU Flow kit (BD Biosciences) following the manufacturer's instructions. The frequency of BrdU‐labeled ASCs was analyzed by flow cytometry.

### 2.8 Analysis of H2AX phosphorylation

In order to quantify the amount of double‐strand DNA breaks (DSBs) in ASCs lacking GARP and analyze the underlying mechanisms, phosphorylated H2AX (γ‐H2AX) was measured by flow cytometry using the FlowCellect Histone H2AX Phosphorylation Assay Kit (Millipore, Massachusetts). To induce DSBs, NT, LV‐CTRL, LV#18 and LV#19, NT(S), and GARP^++^ASCs were seeded in 12‐well plates (50 000 cells/well) and treated with 25 μM etoposide (Sigma‐Aldrich) the following day. Cells were subsequently harvested at different time points (2, 6, and 24 hours after etoposide addition) and stained for γ‐H2AX according to the manufacturer's instructions. In order to study the effect of the inhibition of mtROS and TGF‐β signaling on the induction of DSBs, NT, LV‐CTRL, LV#18 and LV#19 ASCs were seeded in 12‐well plates (50 000 cells/well), 5 hours after silencing of GARP. The following day, SB431542 (10 μM) or MitoTEMPO (25 μM) were added to the cells. Cells were harvested 3 days later, stained for γ‐H2AX, and analyzed by flow cytometry. In order to analyze in more detail the number of γ‐H2AX foci/cell, NT, LV‐CTRL, LV#18, and LV#19 ASCs were stained for γ‐H2AX as described above and acquired on an ImageStream X Mark II Imaging flow cytometer (Millipore) and analyzed using the IDEAS software (Spot Wizard).

In order to study the effect of TGF‐β signaling on the induction of DSBs in GARP‐overexpressing ASCs, 50 000 NT(S) and GARP^++^ASCs were plated in 12‐well plates. After 5 hours, 10 μM of SB431542 was added to the cells. The following day, cells were treated with etoposide (25 μM) and after 6 or 24 hours, the cells were stained for γ‐H2AX as explained above.

### 2.9 ROS measurement

Total ROS was measured using the DCFDA/H2DCFDA—Cellular Reactive Oxygen Species Detection Assay Kit (Abcam, Cambridge, UK). NT, LV‐CTRL, LV#18, and LV#19 ASCs were seeded in 12‐well plates (50 000 cells/well), 4 days after GARP silencing. The next day, the cells were incubated with DCFDA (20 μM) for 30 minutes at 37°C, washed and analyzed by flow cytometry. Mitochondrial ROS (mtROS) was analyzed using MitoSOX (Molecular Probes, Invitrogen). NT, LV‐CTRL, LV#18, and LV#19 ASCs, cultured in the absence or presence of MitoTEMPO (25 μM), were seeded in 12‐well plates (50.000 cells/well), 4 days after GARP silencing. The next day, cells were incubated with MitoSOX (5 μM) for 10 minutes at 37°C. Cells were then incubated with fresh medium for another 3 hours at 37°C, harvested and analyzed by flow cytometry.

### 2.10 Measurement of active TGF‐β

To measure the levels of active TGF‐β in ASCs, NT, LV‐CTRL, LV#19, NT(S), and GARP^++^ASCs were cultured in 12‐well plates (80 000 cells/well) using DMEM supplemented with 0.5% fetal bovine serum. After 2 days, the supernatants of these cells were collected. Recombinant TGF‐β1 (positive control; 1 ng/mL; Peprotech) and conditioned medium (CM) of ASCs were added to 40 000 SMAD‐binding element (SBE)‐HEK293 cells (BPS Bioscience) and plated in a 96‐well Assay Plates (Sigma‐Aldrich). After 18 hours, the SBE activity was analyzed by reading the luciferase induction using the One‐Step Luciferase Assay System (BPS Bioscience) on a Glomax Multi Detection System (Promega) following the manufacturer's instructions.

### 2.11 Statistical analysis

The statistical analysis was performed using the GraphPad Prism software (GraphPad Software, Inc, La Jolla, California). All data are represented as mean (SD) of three independent experiments unless otherwise stated in the figure legend. Multiple comparisons of the data were performed using the one‐way analysis of variance, followed by the Dunnett's or Bonferroni post‐test. Correlation of data was performed using the two‐tailed Pearson test. *P* values ≤.05 were considered statistically significant.

## 3 RESULTS

### 3.1 GARP is required for ASC proliferation and survival

**Figure 1:**
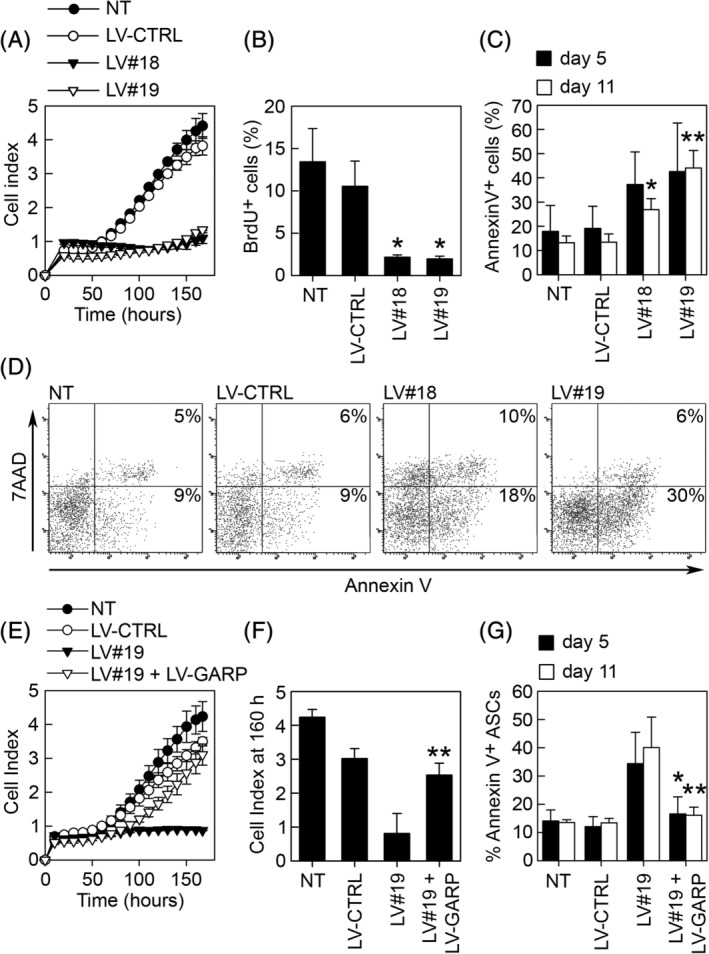
Silencing of GARP inhibits the expansion of ASCs in vitro and induces apoptosis. Human ASCs were transduced with LVs expressing two GARP‐specific shRNAs (LV#18 and LV#19) targeting distinct sequences of the coding region of the GARP mRNA. Non‐transduced (NT) and LV‐CTRL‐transduced ASCs were used as controls. A, The proliferation of NT, LV‐CTRL, LV#18, and LV#19 ASCs were analyzed using the xCelligence real‐time cell analyzer system. Proliferation is represented by cell index, and the data show one representative experiment out of three. B, NT, LV‐CTRL, LV#18, and LV#19 ASCs were pulsed with BrdU for 3 hours and subsequently stained for BrdU‐incorporation and analyzed by flow cytometry. The data are shown as mean (SD) of three independent experiments. *P < .05 vs LV‐CTRL. C, NT, LV‐CTRL, LV#18, and LV#19 ASCs were stained with 7AAD and Annexin V, 5 days (black bars) and 11 days (white bars) after transduction and analyzed by flow cytometry. Data are shown as mean (SD) of four independent experiments. *P < .05 vs LV‐CTRL day 11, **P < .01 vs LV‐CTRL day 11. D, Representative dot plots of 7AAD/Annexin V‐stained NT, LV‐CTRL, LV#18, and LV#19 ASCs on day 5 after transduction. E, The proliferative capacity of NT, LV‐CTRL, LV#19, and LV#19 + LV‐GARP ASCs was analyzed using the xCelligence real‐time cell analyzer system. F, The proliferation data are represented as the cell index at 160 hours. The results are shown as mean (SD) of four independent experiments. **P < .01 vs LV#19. G, The percentages of apoptotic (Annexin V^+^) NT, LV‐CTRL, LV#19, and LV#19 + LV‐GARP ASCs were analyzed on day 5 (black bars) and day 11 (white bars) after transduction by flow cytometry. Results are shown as mean (SD) based on four independent experiments. *P < .05 vs LV#19 day 5, **P < .01 vs LV#19 day 11. ASCs, adipose‐derived mesenchymal stromal cells; GARP, glycoprotein A repetitions predominant; LVs, lentiviral vectors

We have previously shown that GARP is important for the expansion of murine and human ASCs in vitro,^29^ and we wanted to understand the mechanisms behind this observation. In order to silence GARP, we transduced ASCs with LV vectors encoding for two distinct GARP‐targeting shRNAs (LV#18 and LV#19) or a control shRNA (LV‐CTRL). Using the xCelligence real‐time cell analyzer system (Figure 1A) and a BrdU‐incorporation assay (Figure 1B), we confirmed that silencing of GARP in ASCs (GARP^−/low^ASCs) inhibited their proliferation compared with non‐transduced (NT) and control (LV‐CTRL) ASCs. We also observed higher levels of apoptosis in GARP^−/low^ASCs (Figure 1C and D; LV#18 and LV#19) compared with GARP^+^ ASCs (Figure 1C and D; LV‐CTRL and NT), both 5 and 11 days after GARP silencing. Overexpression of GARP in GARP^−/low^ASCs rescued their block in proliferation (Figure 1E and F) and prevented their death by apoptosis (Figure 1G). This effect was seen either when simultaneously co‐transducing ASCs with LV#19 and LV‐GARP (expressing codon‐optimized hGARP, resistant to the shRNAs) or when firstly silencing GARP using LV#19 and subsequently overexpressing GARP the following day (data not shown).

### 3.2 Silencing of GARP affects the transcriptional program of ASCs, modulating genes involved in cellular fitness, cell cycle regulation, and DNA repair

In order to analyze the impact of GARP silencing on the transcriptional program of ASCs and identify potential mechanisms involved in the observed phenotype, we performed a microarray analysis on NT, LV‐CTRL, LV#18, and LV#19 ASCs. We used ASCs from three unrelated donors and silenced GARP using three different batches of LVs (Figures S1A and B). The mRNA was isolated 6 days post‐transduction, a time point at which the proliferative block of GARP^−/low^ASCs was evident. Using linear models, we identified genes differentially expressed in both LV#18‐ and LV#19‐transduced ASCs compared with NT and LV‐CTRL ASCs and obtained a list of 378 genes that were upregulated (>0.5 logFC) and 556 genes that were downregulated (<−0.5 logFC) (Table S1).

**Figure 2:**
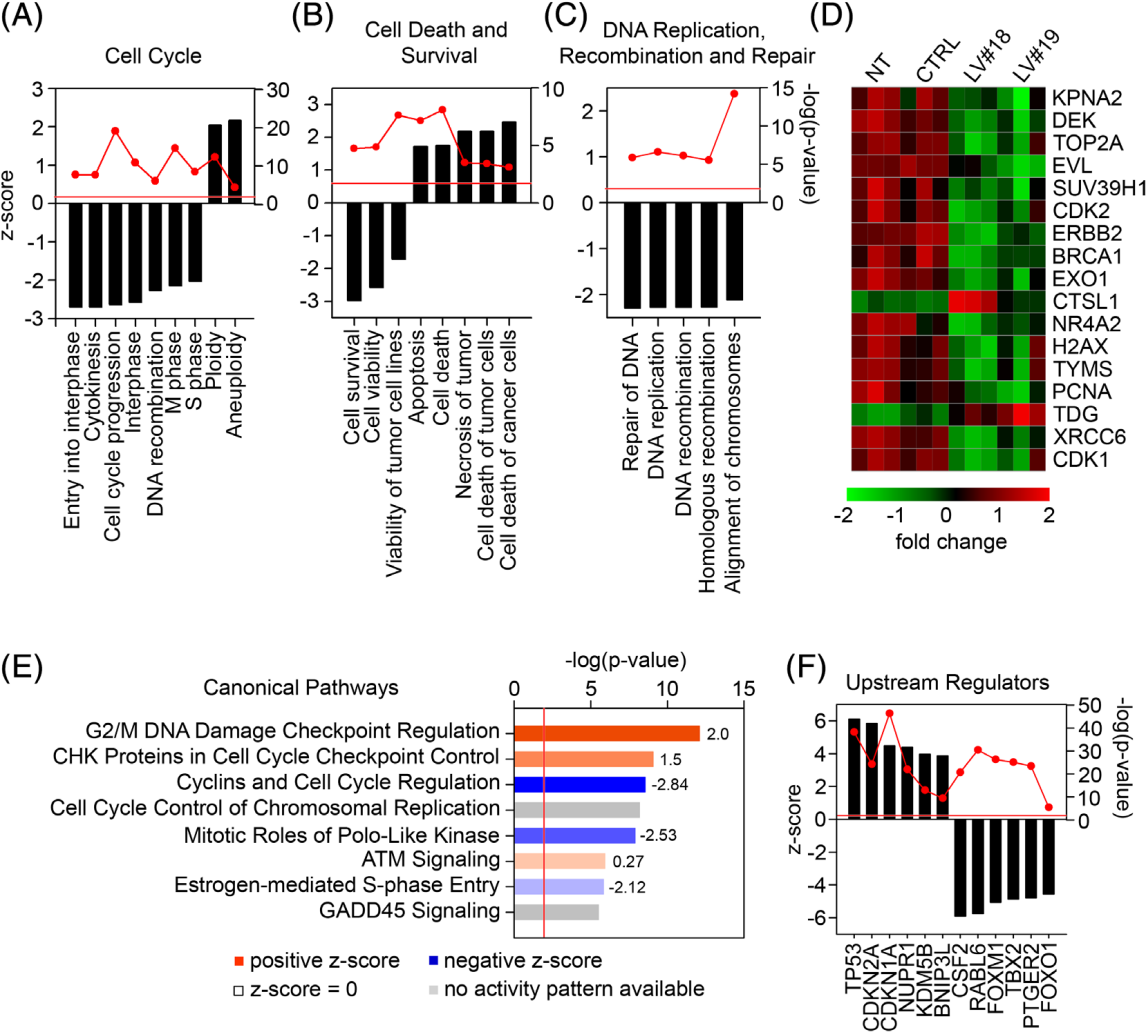
Silencing of GARP affects the transcriptional program of ASCs, modulating genes involved in cell cycle regulation, apoptosis, and DNA repair. Total RNA was isolated from NT, LV‐CTRL, LV#18, and LV#19 ASCs 6 days after transduction, and gene expression was analyzed using the HumanHT‐12v4 Expression BeadChip. An ingenuity pathway analysis (IPA) of the modulated genes was performed and revealed several biological categories and biofunctions significantly affected in GARP^−/low^ASCs compared with NT and LV‐CTRL ASCs. In‐depth analysis of the biological categories “Cell Cycle” (A), “Cell Death and Survival” (B), and “DNA Replication, Recombination and Repair” (C), showing the most prominent biofunctions (black bars) and their predicted activation (positive z‐score) or inhibition (negative z‐score). Red circles show the statistical significance for each biofunction and the red line represents *P* = .01. D, Heatmap showing the top significantly changed genes (LV#18/LV#19 vs NT/LV‐CTRL) in the biofunction “DNA Replication, Recombination and Repair.” E, IPA prediction of activated/inhibited canonical pathways that were significantly overrepresented in GARP^−/low^ASCs compared with NT and LV‐CTRL ASCs. Bar colors represent the predicted activation (red), inhibition (blue), z‐score = 0 (no color), and no activity pattern available (grey) based on the z‐score. The values next to the bars represent the z‐scores when available. The red line represents *P* = .01. F, IPA prediction of upstream regulators, activated (positive z‐score) or inhibited (negative z‐score), responsible for the obtained gene expression profile in GARP^−/low^ASCs. Red circles show the statistical significance for each biofunction and the red line represents *P* = .01. ASCs, adipose‐derived mesenchymal stromal cells; GARP, glycoprotein A repetitions predominant; LVs, lentiviral vectors

As expected, the IPA software identified several inhibited biofunctions in the “Cell Cycle” category required for cell cycle progression (Figure 2A) and several activated biofunctions related to apoptosis and cell death in the “Cell Death and Survival” category (Figure 2B). Interestingly, many biofunctions in “DNA replication, Recombination, and Repair” category were downregulated in GARP^−/low^ASCs (Figure 2C). A detailed view of the genes affected in the DNA repair biofunction showed a downregulation of several genes involved in maintaining genomic stability (*BRCA1*, *TOP2A*, *TYMS*) and DSB repair (*EXO1*, *PCNA*) (Figure 2D).

Investigating the effects of GARP‐silencing on the activation/inhibition of canonical pathways in ASCs, the IPA highlighted the activation of the “G2/M DNA Damage Checkpoint Regulation” (z‐score = 2.0) pathway and the inhibition of the “Mitotic Roles of Polo‐like Kinase” (z‐score = −2.84) pathway (Figure 2E). The alterations in these two pathways are suggestive of a block in the G2/M phase of the cell cycle due to DNA damage and/or DNA replication defects in GARP^−/low^ASCs. Finally, the IPA also identified tumor protein (TP)53 as the top activated upstream regulator (Figure 2F). TP53 contributes to the maintenance of the G2/M checkpoint via the transcriptional repression of CDC25C, cyclin B, and CDK1.^46^ In agreement, these genes were downregulated in GARP^−/low^ASCs compared with NT and LV‐CTRL ASCs (Table S1). In addition, the expression of several TP53‐inducible antioxidant genes were upregulated in the GARP^−/low^ASCs, including *SESN1*, *SPATA18*, and *GPX1* 47, 48 (Table S1). All together, these data suggested that GARP silencing could lead to DNA damage and proliferation block in the G2/M phase through activation of TP53.

### 3.3 GARP^−/low^ASCs are blocked in the G2/M phase and exhibit increased DNA damage

**Figure 3:**
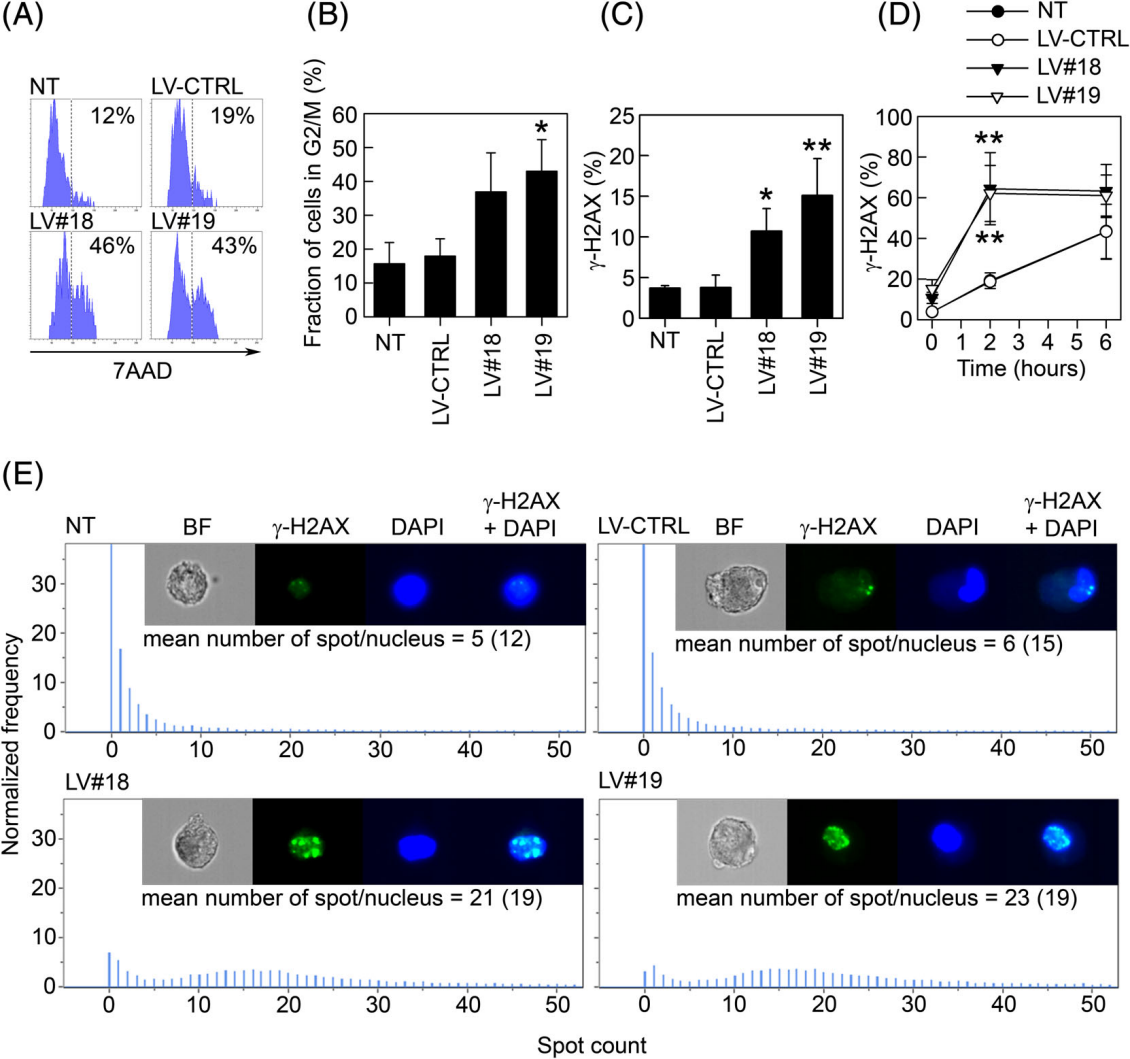
GARP^−/low^ASCs are blocked in G2/M and exhibit increased DNA damage. A, ASCs were transduced with LV‐CTRL, LV#18, and LV#19 and the following day pulsed with BrdU for 3 hours. After 3 days, the cells were harvested and stained for BrdU and 7AAD and analyzed by flow cytometry. The histograms show the BrdU^+^ cells in the phases of the cell cycle visualized by 7AAD. One representative experiment out of three is shown. B, Fraction of BrdU^+^ cells in the G2/M phase of the cell cycle. Data are shown as mean (SD) of three independent experiments. *P < .05 vs LV‐CTRL. C, Phosphorylation of H2AX (γ‐H2AX) was analyzed in NT, LV‐CTRL, LV#18, and LV#19 ASCs by flow cytometry 5 days after silencing of GARP. Data are shown as mean (SD) of three independent experiments. *P < .05 vs LV‐CTRL, **P < .01 vs LV‐CTRL. D, NT, LV‐CTRL, LV#18, and LV#19 ASCs were stimulated with etoposide (25 μM) for different time points, stained for γ‐H2AX and analyzed by flow cytometry. Data are shown as mean (SD) of three independent experiments. **P < .01 vs LV‐CTRL (2 hours). E, NT, LV‐CTRL, LV#18, and LV#19 ASCs were stained for γ‐H2AX, 5 days after GARP silencing and analyzed on an ImageStream X Mark II imaging flow cytometer. The numbers of γ‐H2AX foci/nuclei were quantified using the software IDEAS (Spot Wizard). ASCs, adipose‐derived mesenchymal stromal cells; BrdU, 5‐bromo‐2′´‐deoxyuridine; GARP, glycoprotein A repetitions predominant; LVs, lentiviral vectors

In order to verify the predicted G2/M block, we analyzed the cell cycle kinetics of GARP^−/low^ASCs using BrdU and 7AAD. As predicted by the IPA, higher percentages of BrdU^+^ cells were observed in the G2/M phase of GARP^−/low^ASCs, compared with LV‐CTRL and NT ASCs (Figure 3A and B). As the IPA also suggested a downregulation of the DNA repair machinery, we hypothesized that the observed G2/M block could be a transitory state of the GARP^−/low^ASCs before entering apoptosis due to excessive DNA damage. We therefore studied the degree of DSBs in GARP^−/low^ASCs. H2AX is a key factor in the repair process of damaged DNA and its phosphorylated form (γ‐H2AX) serves as a biomarker for DSBs.^49^ Using flow cytometry, we found that both basal (Figure 3C) and etoposide‐induced (Figure 3D) γ‐H2AX levels were significantly higher in GARP^−/low^ASCs compared with NT and LV‐CTRL ASCs indicating that GARP^−/low^ASCs are more exposed or susceptible to DNA damage. In addition to the FACS analysis, we quantified the number of γ‐H2AX foci in the nuclei in NT, LV‐CTRL, and GARP^−/low^ASCs using an Imaging flow cytometer. These data confirmed our previous results, showing a fourfold increase in foci formation in GARP^−/low^ASCs compared with control cells (Figure 3E).

### 3.4 Inhibition of mtROS reduced the DSBs and reversed the proliferation block in GARP^−/low^ASCs

**Figure 4:**
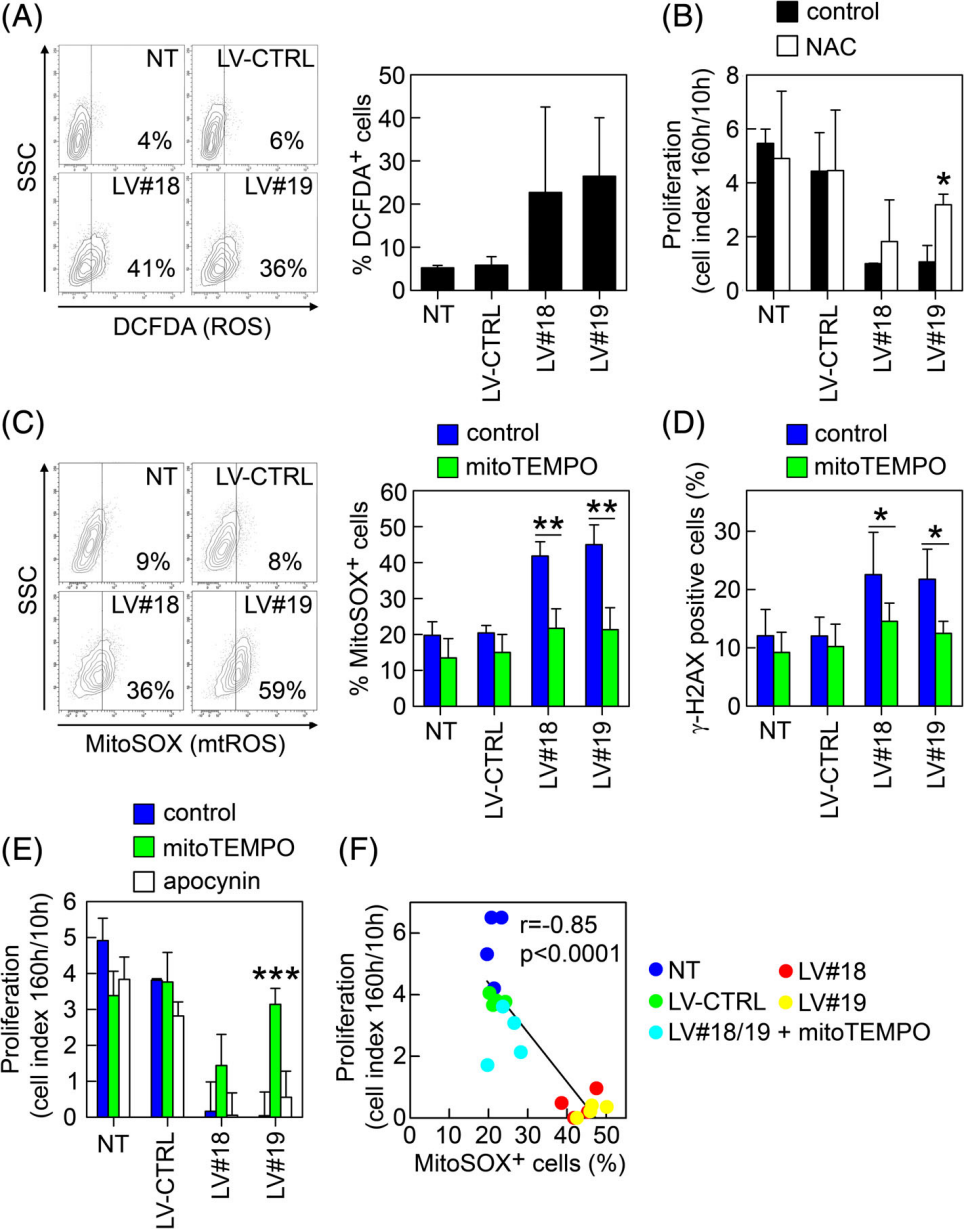
Inhibition of mitochondrial reactive oxygen species (mtROS) reduces the DSBs and reverses the proliferation block in GARP^−/low^ASCs. A, ASCs were transduced with LV‐CTRL, LV#18, and LV#19 and incubated with DCFDA (20 μM) and analyzed by flow cytometry. Representative dot plots showing the percentage of DCFDA^+^ cells in relation to nonstained cells (left panel). Comparison of DCFDA^+^ NT, LV‐CTRL, LV#18, and LV#19 ASCs (right panel). Data are shown as mean (SD) of three independent experiments. B, NT, LV‐CTRL, LV#18, and LV#19 ASCs were cultured with (white bars) or without 1 mM NAC (black bars), and their proliferation was analyzed using the xCelligence real‐time cell analyzer system. Proliferation is represented as cell index at 160 hours/cell index at 10 hours. Data are shown as mean (SD) of three independent experiments. *P < .05 vs LV#19 control. C, In order to measure mtROS, NT, LV‐CTRL, LV#18, and LV#19 ASCs were cultured in the absence or presence of mitoTEMPO (25 μM), labeled with MitoSOX (20 μM) and analyzed by flow cytometry. Representative dot plots showing the percentage of MitoSOX^+^ cells in relation to nonstained cells (left panels). Graph showing the percentages of MitoSOX^+^ NT, LV‐CTRL, LV#18, and LV#19 ASCs (right panel). Data are shown as mean (SD) of three independent experiments. **P < .01 vs LV‐CTRL. D, NT, LV‐CTRL, LV#18, and LV#19 ASCs were cultured without (control, blue bars) or with 25 μM mitoTEMPO (green bars) and the levels of γ‐H2AX were analyzed 4 days after transduction by flow cytometry. Data are shown as mean (SD) or four independent experiments. **P < .01. E, Proliferation of NT, LV‐CTRL, LV#18, and LV#19 ASCs cultured without (blue bars) and with mitoTEMPO (green bars) and apocynin (white bars). Proliferation is shown as cell index at 160 hours/cell index at 10 hours. Data are shown as mean (SD) of three independent experiments. ***P < .001 vs LV#19 control. F, Pearson correlation between the levels of mtROS (MitoSOX^+^ cells) in NT, LV‐CTRL, and LV#18 and LV#19 ASCs (treated or not with mitoTEMPO) and their proliferative capacity. Pearson *r* = −.85. Data are plotted from four independent experiments. ASCs, adipose‐derived mesenchymal stromal cells; DSBs, double‐strand DNA breaks; GARP, glycoprotein A repetitions predominant; LVs, lentiviral vectors

ROS are well‐known inducers of both single‐ and double‐stranded DNA lesions^50^ and apoptosis.^51^ Thus, we set out to analyze whether silencing of GARP would affect the levels of ROS in ASCs using DCFDA. Interestingly, we observed elevated ROS levels in GARP^−/low^ASCs compared with NT and LV‐CTRL ASCs (Figure 4A). Addition of the ROS‐scavenger N‐acetyl cystein (NAC) significantly reverted the proliferation block in GARP^−/low^ASCs (Figure 4B). In MSCs, ROS is mainly produced by the mitochondrial complexes I and III (mtROS) and, to a lesser extent, by the NADPH oxidase (NOX)‐4.^52^ We showed that GARP^−/low^ASCs produced higher levels of mtROS compared with control cells (Figures 4C, plots) that could be reversed by the addition of mitoTEMPO (a mitochondrially targeted antioxidant) (Figure 4C, graph, green bars). Importantly, inhibition of mtROS reduced the levels of DSBs in GARP^−/low^ASCs (Figure 4D), pointing to mtROS as the main trigger of the DSB increment observed after downregulation of GARP. Finally, we found that mitoTEMPO, but not apocynin (a NOX inhibitor), rescued the proliferation of GARP^−/low^ASCs to values near to control cells (Figure 4E). In addition, there was an inverse correlation between the proliferative capacity of the ASCs and their mtROS levels (Figure 4F). In summary, these data suggest that the loss of GARP results in an increase in mtROS which damage the DNA, resulting in an inhibition of cell proliferation.

### 3.5 Inhibition of TGF‐β signaling in GARP^−/low^ASCs reduced mtROS levels, DNA damage, and partially reversed the block in proliferation

**Figure 5:**
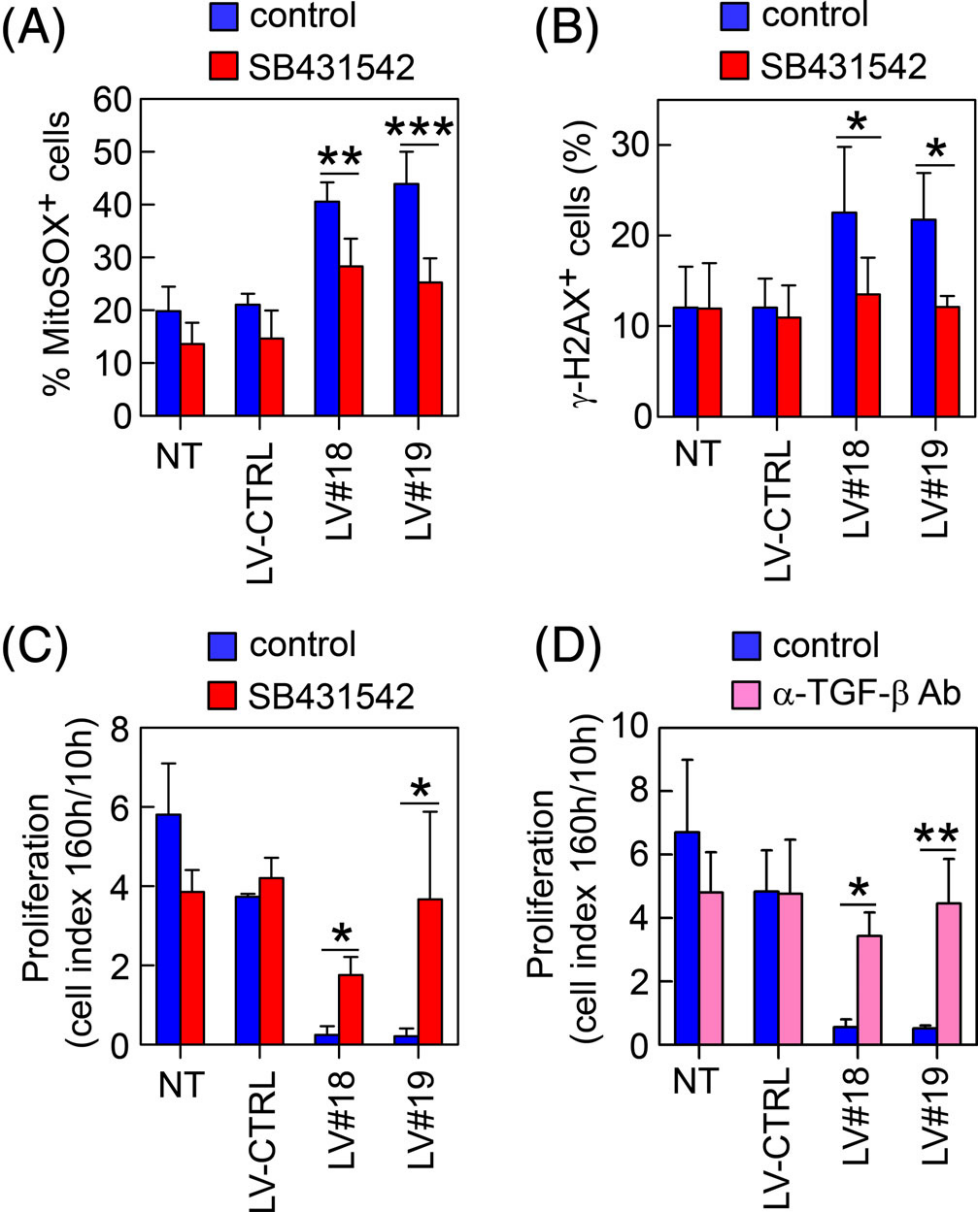
Inhibition of TGF‐β signaling in GARP^−/low^ASCs reduced mtROS, DNA damage, and partially reverted the proliferation block. NT, LV‐CTRL, LV#18, and LV#19 ASCs were cultured without (blue bars) or with 10 μM SB431542 (red bars) and incubated with (A) MitoSOX to analyze the levels of mtROS or (B) stained for γ‐H2AX. Data are shown as mean (SD) of three independent experiments. *P < .05 vs LV#18 or LV#19 control, **P < .01 vs LV#18 control, and ***P < .001 vs LV#19 control. We used the same controls as in Figure 5C (MitoSOX) and Figure 5D (γ‐H2AX) as SB431542, and mitoTEMPO were added simultaneously in the same set of experiments. C, Graph showing the proliferation of NT, LV‐CTRL, LV#18, and LV#19 ASCs cultured without (blue bars) or with SB431542 (red bars). Proliferation was calculated as the cell index at 160 hours/cell index at 10 hours. Data are shown as mean (SD) of three independent experiments. *P < .05 vs LV#18 control, *P < .05 vs LV#19 control. D, Graph showing the proliferation of NT, LV‐CTRL, LV#18, and LV#19 ASCs cultured without (blue bars) or with an anti‐TGF‐β1/2/3 Ab (magenta bars). Proliferation was calculated as the cell index at 160 hours/cell index at 10 hours. Data are shown as mean (SD) of three independent experiments. *P < .05 vs LV#18 control, **P < .01 vs LV#19 control. ASCs, adipose‐derived mesenchymal stromal cells; GARP, glycoprotein A repetitions predominant; LVs, lentiviral vectors; TGF‐β, transforming growth factor‐β

We have previously shown that silencing of GARP in murine ASCs increased their secretion and activation of TGF‐β1^29^ and similarly, silencing GARP in human ASCs increased their production of active TGF‐β (Figure S2). Recent reports have demonstrated that TGF‐β1 can induce mtROS production in MSCs with effects on their proliferation and survival.^53^, ^54^ We found that blocking TGF‐β signaling using SB431542^55^ in GARP^−/low^ASCs, just 1 day after GARP‐silencing, reduced their mtROS levels (Figure 5A), H2AX‐phosphorylation (Figure 5B), and significantly increased their proliferative capacity (Figure 5C). Furthermore, addition of an anti‐TGF‐β1/2/3 antibody (1D11), 1 day after GARP silencing, also reversed the block in proliferation of GARP^−/low^ASCs (Figure 5D). These data suggest that, in the absence of GARP, TGF‐β1 increases the levels of mtROS which induce DSBs and inhibit ASC proliferation.

### 3.6 Overexpression of GARP in ASCs promotes their proliferation and enhances their resistance to apoptosis and DNA damage via TGF‐β signaling

**Figure 6:**
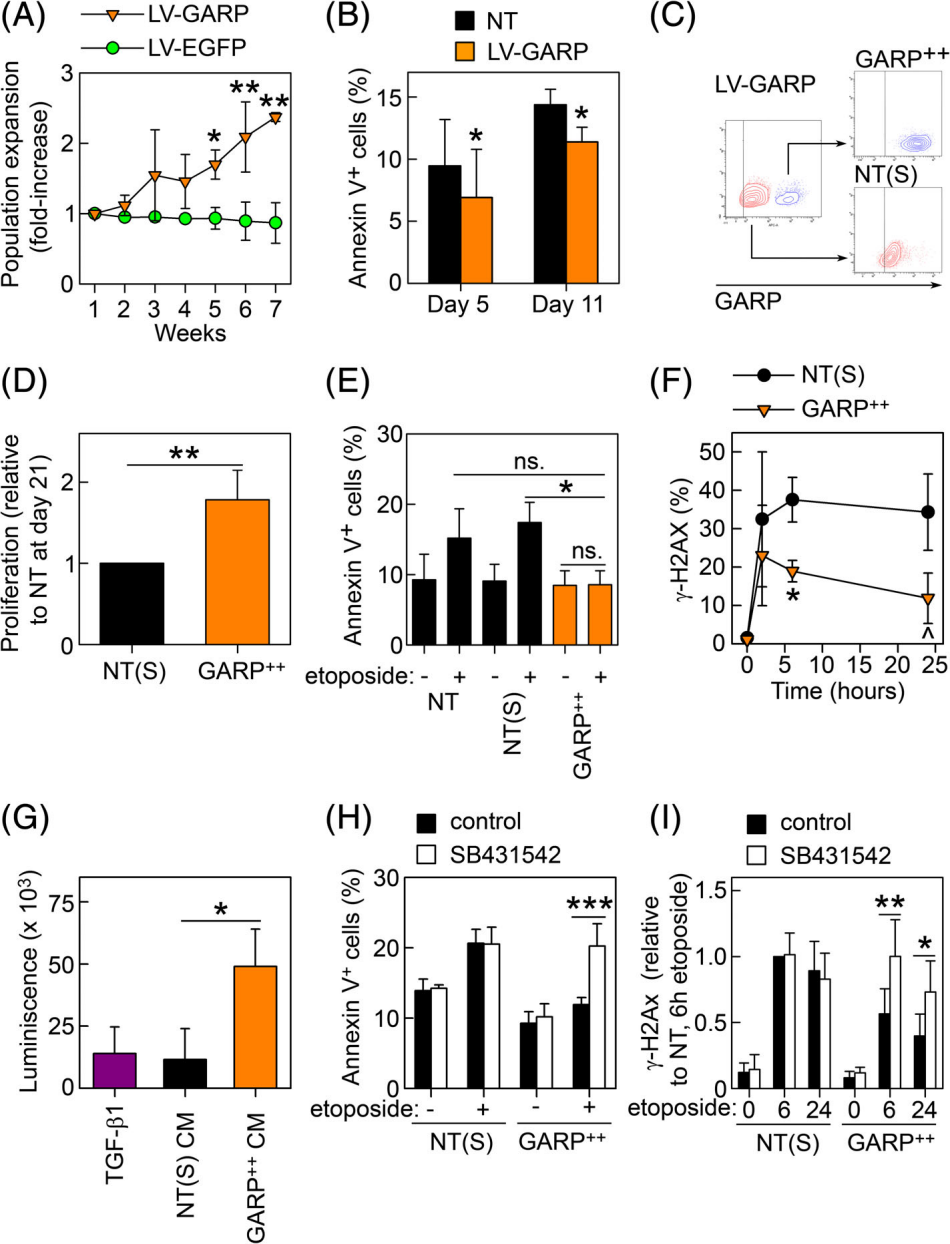
GARP‐mediated activation of TGF‐β protects ASCs against DNA damage and apoptosis and promotes their proliferation. A, ASCs were transduced with LV‐GARP and LV‐EGFP, and the size (%) of the GARP‐ and EGFP‐overexpressing populations in the bulk culture was followed over time by flow cytometry. Data are shown as mean (SD) of three experimental replicates. *P < .05 LV‐GARP vs LV‐EGFP at week 5, **P < .01 LV‐GARP vs LV‐EGFP at weeks 6 and 7. B, NT (black bar) and LV‐GARP‐transduced ASCs (orange bar) were stained with Annexin V/7AAD on days 5 and 11 after transduction, and the amount of apoptotic cells (Annexin V^+^) was analyzed by flow cytometry. Data are shown as mean (SD) of four independent experiments. *P < .05 LV‐GARP vs NT. C, GARP‐overexpressing cells (GARP^++^, upper right dot plot) were separated from NT ASCs (NT(S), lower left dot plot) using an FACSAria cell sorter, and their proliferative capacity was analyzed comparing the total cell number of GARP^++^ASC relative to NT(S) ASCs after 21 days of culturing. Results are shown as mean (SD) of three independent experiments. **P < .01. E, NT, NT(S), and GARP^++^ASCs were cultured with or without etoposide (25 μM) for 2 days and subsequently stained for Annexin V and analyzed by flow cytometry. Results are shown as mean (SD) of three independent experiments. *P < .05, ns = not significant. F, NT(S) and GARP^++^ASCs were stimulated with etoposide (25 μM) for 0, 2, and 6 hours and subsequently stained for γ‐H2AX and analyzed by flow cytometry. Data are shown as mean (SD) of three independent experiments. *P > .05 vs LV‐CTRL, ^*P* < .05 LV‐GARP vs NT at 24 hours. G, Recombinant TGF‐β1 (1 ng/mL) and conditioned medium (CM) from NT(S) and GARP^++^ASCs were added to SBE‐HEK293 cells for 18 hours, and luminescence were read on a Glomax Multi Detection System (Promega). Data are shown as mean (SD) of three independent experiments. *P < .05. H, NT(S) and GARP^++^ASCs were cultured with 25 μM etoposide for 48 hours in the absence (black bars; control) and presence of SB431542 (white bars), stained for AnnexinV/7AAD and analyzed by flow cytometry. ***P < .001. I, NT(S) and GARP^++^ASCs were cultured with 25 μM etoposide for 0, 6, and 24 hours in the absence (black bars; control) and presence of SB431542 (white bars), stained for γ‐H2AX and analyzed by flow cytometry. *P < .05, **P < .01. ASCs, adipose‐derived mesenchymal stromal cells; GARP, glycoprotein A repetitions predominant; LVs, lentiviral vectors; TGF‐β, transforming growth factor‐β

To further investigate the effect of GARP on cell proliferation and survival, we generated several ASCs lines overexpressing GARP in 30%‐50% of the population using LV‐GARP and analyzed changes in the percentage of the GARP‐overexpressing cells along time in culture and the level of apoptosis. We found that the percentage of GARP‐overexpressing ASCs increased in culture with time suggesting a proliferative advantage of these cells over NT and EGFP‐overexpressing (LV‐EGFP) ASCs (Figure 6A). We also observed significantly less LV‐GARP ASCs undergoing apoptosis compared with NT ASCs, both 5 and 11 days after transduction (Figure 6B). We then separated the non‐transduced (NT(S)) and GARP‐overexpressing (GARP^++^) ASCs (Figure 6C) and analyzed their proliferation and susceptibility to apoptosis‐inducing stimuli. As seen in the bulk cultures, GARP^++^ASCs proliferated more compared with NT(S) ASCs (Figure 6D). Interestingly, GARP^++^ASCs were more resistant to etoposide‐induced apoptosis (Figure 6E) and DNA damage, as visualized by γ‐H2AX (Figure 6F), compared with NT(S) ASCs. In summary, these data show that, in contrary to the silencing of GARP, overexpression of GARP in ASCs promotes their proliferation and increases their resistance to apoptosis and DNA damage.

Finally, using a TGF‐β‐responsive reporter cell line, we found that CM of GARP^++^ASCs contained significantly higher levels of active TGF‐β compared with NT(S) ASCs, showing that GARP also promote TGF‐β activation by ASCs (Figure 6G). Inhibition of TGF‐β signaling using SB431542 significantly reversed the protective effects of GARP on etoposide‐induced apoptosis (Figure 6H) and DNA damage (Figure 6I). Our data suggest that presence or absence of GARP gives rise to two distinct TGF‐β responses with diametrically opposing effects on ASC proliferation and survival.

## 4 DISCUSSION

MSCs represent a promising cell‐based therapy in regenerative medicine, inflammatory/autoimmune diseases, and cancer. However, basic research on how to improve the proliferation, survival, and immunomodulation of MSCs is of fundamental importance for their successful translation into clinical applications. We have previously shown that murine ASCs express GARP/LRRC32 which is important for their immunosuppressive and proliferative capacities.^29^ The aim of the current study was to investigate the mechanism behind the block in proliferation of GARP‐silenced human ASCs and analyze the effect of GARP‐overexpression on ASC proliferation and survival.

We performed a microarray analysis comparing gene expression in GARP‐positive and GARP^−/low^ASCs. In accordance to the phenotype, several genes important for MSC proliferation were downregulated in GARP^−/low^ASCs, including six of seven recently identified predictive gene markers for MSC proliferation (PLK1, CDC20, BIR5C, SPC25, PBK, and CCNA2).^56^ An IPA identified TP53 as a possible activated upstream regulator that could explain the obtained gene expression profile in GARP^−/low^ASCs. This is in agreement with Zhou and colleagues who showed that GARP^low^HeLa cells proliferated less and exhibited an increased expression of TP53, p21/Cip1, and p27/Kip1 compared with GARP^intermediate^HeLa and GARP^high^HeLa cells.^32^ Our IPA analysis also revealed an inhibition of the DNA repair biofunction, suggesting an increase in DNA damage in the absence of GARP. In line with these predictions, we found that GARP^−/low^ASCs exhibited higher basal levels of DSBs and were also more susceptible to etoposide‐induced DNA damage.

Considering the potential links between GARP and DNA damage, several pieces of evidence suggested the involvement of TGF‐β and ROS. Firstly, we have previously shown that silencing of GARP in mASCs resulted in an increased activation of TGF‐β1, an increase in SMAD2/3‐phosphorylation, and the induction of TGF‐β1‐responsive genes in GARP^−/low^mASCs.^29^ Secondly, it was recently reported that TGF‐β1 can increase the ROS production in murine BM‐MSCs, which resulted in senescence^53^ and apoptosis.^54^ We thus measured the ROS levels in GARP^−/low^ASCs and detected a significant increase in the levels of mtROS compared with control cells. Although physiological levels of ROS in MSCs are necessary for their proliferation^57^ and adipogenic/chondrogenic differentiation capacity,^58^ high endogenous ROS levels can have detrimental effects on MSCs, including the induction of DNA damage and premature senescence,^59^ impairment of MSC migration,^60^ and a reduction in their immunomodulatory^61^ and osteogenic capacities.^62^ We found that reducing the levels of mtROS in GARP^−/low^ASCs using the mitochondria‐targeted antioxidant mitoTEMPO, decreased the amount of DSBs, and partially reversed their block in proliferation. Importantly, inhibition of TGF‐β signaling in GARP^−/low^ASCs reduced their levels of mtROS and DSBs and almost completely reversed the block in proliferation. These data suggest that, in the absence of GARP, TGF‐β activation increases which induces a deleterious response in ASCs through its induction of mtROS, DNA damage, and apoptosis.

In stark contrast, overexpression of GARP in ASCs also increased their TGF‐β activation, which however rendered the cells more resistant to etoposide‐induced DNA damage and apoptosis. Thus, the question arises as to how both the absence and overexpression of GARP can increase TGF‐β activation which then induces diametrically opposing responses. Considering the two scenarios regarding the bioavailability and activation of TGF‐β, we believe that, in the absence of GARP, ASC‐produced LAP/TGF‐β is secreted and targeted to the extracellular matrix where it has been shown to be activated by thrombospondin‐1.^63^, ^64^ In contrast, GARP‐mediated activation of TGF‐β occurs on the cell surface in a cell‐cell contact‐dependent manner,^21^, ^65^ which results in a qualitatively distinct response due to a higher local concentration of the ligand in the synapse^66^ and/or to the presence of additional cell‐cell interactions.^67^ Although we have not characterized the nature of the TGF‐β responses in GARP^−/low^ and GARP^++^ASCs, TGF‐β has been shown to cause cellular redox imbalance by inducing mtROS via the mTOR pathways^68^ and depleting glutathione via the JNK and induced activating transcription factor 3 pathway,^69^ which can result in DNA damage. In contrast, TGF‐β has also been shown to protect against genomic instability by enhancing nonhomologous end‐joining repair,^70^ ATM activity,^71^ and the SMAD3/β2spectrin/Fanconi anemia DNA repair pathway.^72^

Importantly, three TGF‐β isoforms (TGF‐β1, TGF‐β2, and TGF‐β3) exist which bind to the same receptors but exhibit distinct functions in vivo, in part due to their different tissue distributions and expression levels.^21^ Most of the work on GARP‐mediated activation of TGF‐β has focused on the TGF‐β1 isoform but GARP can also bind TGF‐β2 with low affinity^25^ and activate TGF‐β3.^73^ Future studies should investigate the role of GARP in the activation of TGF‐β2, and TGF‐β3, especially during MSC differentiation into chondrocytes where these isoforms play important roles.^21^

Whether GARP, apart from its effects on TGF‐β activation, is directly involved in the DNA damage or antioxidant responses is unknown. However, since GARP contains the highly frequent protein‐protein interacting domain, the leucine‐rich repeat (LRR) motif, the possibility for GARP to interact with other proteins is broad. So far, GARP has been described to interact with LAP/TGFβ,^74^ lysosomal‐associated transmembrane protein 4B (LAPTMP4B)^75^ and with HSP90A^75^ and HSP90B1 (GP96),^76^ members of the HSP90 family of chaperones, in the cytosol and endoplasmic reticulum, respectively. It would be interesting to investigate if GARP interacts with other HSP90 members, since the HSP90 chaperone machinery plays a key role in orchestrating stress responses and regulating the activity of proteins involved in cell cycle and the DNA damage response, such as BRCA1, BRCA2, RAD51, p53, and CHK1.^77^

Regardless of the mechanisms, our data show that GARP converts a potentially deleterious TGF‐β response into an increased proliferative/anti‐apoptotic response in ASCs during their expansion in vitro. Apart from inducing mtROS and DNA damage, uncontrolled TGF‐β signaling in MSCs has been shown to induce the formation of stress fibers and aberrant differentiation in vitro and to promote osteoarthritis^39^, ^78^, ^79^ and fibrosis‐related diseases^80^ in vivo, which also might be inhibited by GARP. The development of MSC products with high expansion capacity and resistance to in vivo infusion/transplantation would most likely increase the success of MSCs in cell therapies for regenerative medicine, autoimmune/inflammatory diseases, and cancer. Since overexpression of GARP in ASCs protected them from DNA damage and reduced their sensitivity to apoptosis, we propose that GARP overexpression in MSCs could be a potential alternative to generate improved MSCs for therapeutic applications. In this sense, GARP‐overexpressing MSCs could be advantageous for the treatment of inflammatory/autoimmune diseases as the GARP/TGF‐β axis promotes immune tolerance, in part, through the induction of Tregs.^81^ However, GARP/TGF‐β was recently shown to promote the osteogenic differentiation of MSCs in vitro.^82^ Thus, GARP^++^MSCs could be ideal for bone regeneration applications while giving rise to unwanted differentiation when used in inflammatory/autoimmune diseases. In addition, GARP has also been found to promote the growth and spread of tumor cells, classifying it as a possible oncogene.^33^ In the context of MSCs, this is probably not a problem as our data suggest that GARP protects against DNA damage and transformation of human MSCs has not yet been observed in vivo. Future studies should analyze the survival and therapeutic efficacy of GARP^++^MSCs in preclinical models of tissue regeneration and inflammation/autoimmunity, also focusing on possible adverse events associated with increased TGF‐β activation, including unwanted differentiation, tumorigenesis and fibrosis.^83^ It will also be interesting to address whether the observed role of GARP in protecting against TGF‐β‐induced DNA damage is specific to ASCs or if these results are reproducible in other GARP expressing cells, including GARP‐expressing tumor cells.^33^ In this sense, GARP could emerge as a therapeutic target for certain cancers known to exhibit an increased GARP expression, such as breast and colorectal cancer.

## 5 CONCLUSIONS

In summary, our data suggest that GARP protects ASCs against deleterious TGF‐β activation in vitro, preventing oxidative DNA damage and cell death. In contrast, GARP‐overexpressing ASCs exhibit increased proliferation and are less susceptible to DNA damage. We propose that overexpression of GARP in MSCs could increase their therapeutic efficacy in clinical applications.

## CONFLICT OF INTEREST

A.B.C‐G., F.M., P.A. declared patent ownership (registered patent number P201331730, entitled “A novel surface marker of multipotent mesenchymal stromal cells”). The other authors declared no potential conflicts of interest.

## AUTHOR CONTRIBUTIONS

A.B.C.‐G.: conception and design, collection and/or assembly of data, data analysis and interpretation, manuscript writing, final approval of manuscript; S.G.‐P., J.E.G.‐C., M.de.H.‐C., P.M.: collection and/or assembly of data, data analysis and interpretation, final approval of manuscript; V.A., P.C.‐S., V.R.‐M.: data analysis and interpretation, manuscript writing, final approval of manuscript; P.G.‐M.: financial support, data analysis and interpretation, final approval of manuscript; S.G.‐P: provision of study material or patients, data analysis and interpretation, final approval of manuscript; F.E.C.: data analysis and interpretation, final approval of manuscript; F.M., P.A.: conception and design, financial support, data analysis and interpretation, manuscript writing, final approval of manuscript.

## Supporting information


**Figure S1** **Phenotypic and functional validation of NT, LV‐CTRL, LV#18 and LV#19 ASCs used for the microarray analysis**. The titers of LVs were determined in each experiment and we used a MOI~10 to obtain 2‐3 LV integrations/cell (data not shown). The silencing of GARP was assessed by FACS and the proliferation of NT, CTRL, LV#18 and LV#19 ASCs was analyzed for each experiment. (A) GARP expression was measured on the surface of NT, LV‐CTRL, LV#18 and LV#19 ASCs (E19, E23 and E27) by flow cytometry, 4 days after transduction (GARP^+^ ASC [% in relation to isotype control staining]) and plotted against their respective mRNA value obtained from the microarray analysis. (B) The proliferation of NT, LV‐CTRL, LV#18 and LV#19 ASCs (E19, E23 and E27) was analyzed using the xCelligence real‐time cell analyzer system.

**Figure S2** **Silencing of GARP in human ASCs increases their activation of TGF‐β**. Recombinant TGF‐β1 (1 ng/mL) and conditioned medium (CM) from NT, LV‐CTRL and LV#19 ASCs were added to SBE‐HEK293 cells for 18 hours and luminescence was read on a Glomax Multi Detection System (Promega). Data are shown as mean(SD) of three independent experiments. * = *P* < 0.05.

**Table S1** Genes down‐ and upregulated in LV#18/LV#19 vs NT/LV‐CTRL ASCs.

## Data Availability

The data that support the findings of this study are available from the corresponding author upon reasonable request.
