# Supplementary figures and images for: GARP is a key molecule for mesenchymal stromal cell responses to TGF‐β and fundamental to control mitochondrial ROS levels
Source: Stem Cells Transl Med. 2020 Feb 19;9(5):636–50. doi: 10.1002/sctm.19-0372 (PMC7180295; doi:10.1002/sctm.19-0372)

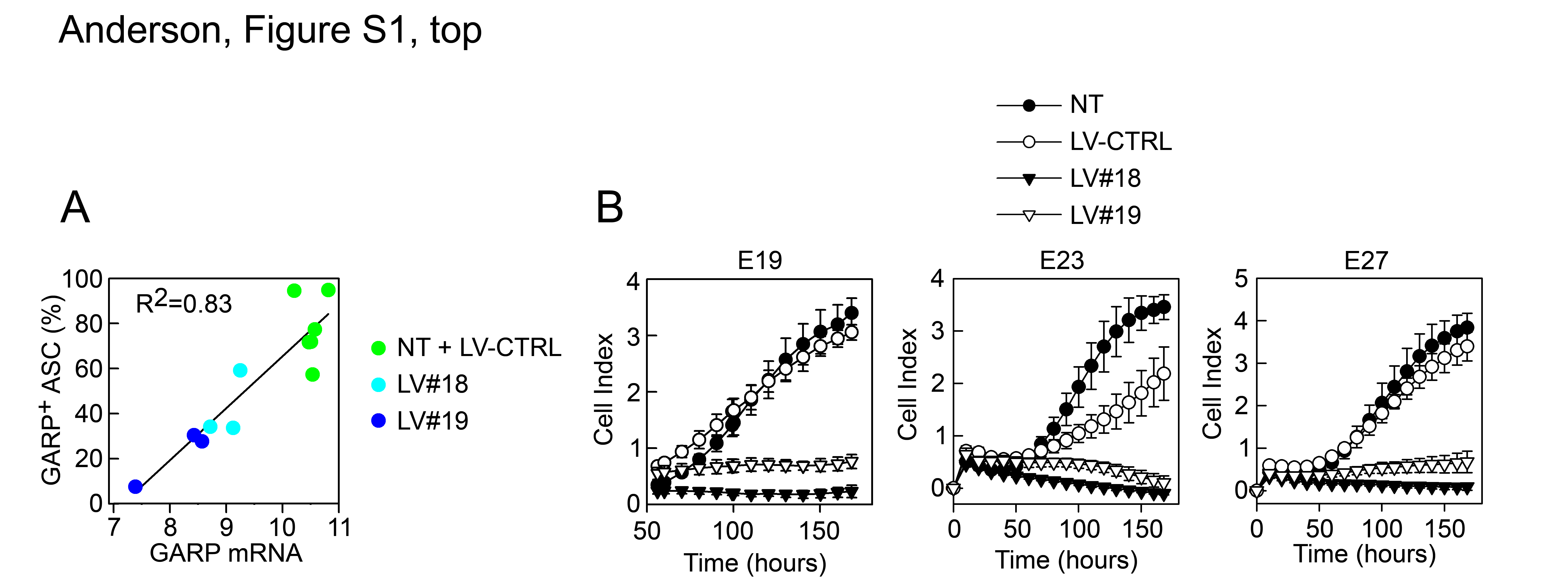

Supplement: Supplementary file 1 — Figure S1 Phenotypic and functional validation of NT, LV‐CTRL, LV#18 and LV#19 ASCs used for the microarray analysis. The titers of LVs were determined in each experiment and we used a MOI~10 to obtain 2‐3 LV integrations/cell (data not shown). The silencing of GARP was assessed by FACS and the proliferation of NT, CTRL, LV#18 and LV#19 ASCs was analyzed for each experiment. (A) GARP expression was measured on the surface of NT, LV‐CTRL, LV#18 and LV#19 ASCs (E19, E23 and E27) by flow cytometry, 4 days after transduction (GARP+ ASC [% in relation to isotype control staining]) and plotted against their respective mRNA value obtained from the microarray analysis. (B) The proliferation of NT, LV‐CTRL, LV#18 and LV#19 ASCs (E19, E23 and E27) was analyzed using the xCelligence real‐time cell analyzer system. [file SCT3-9-636-s001.tif]

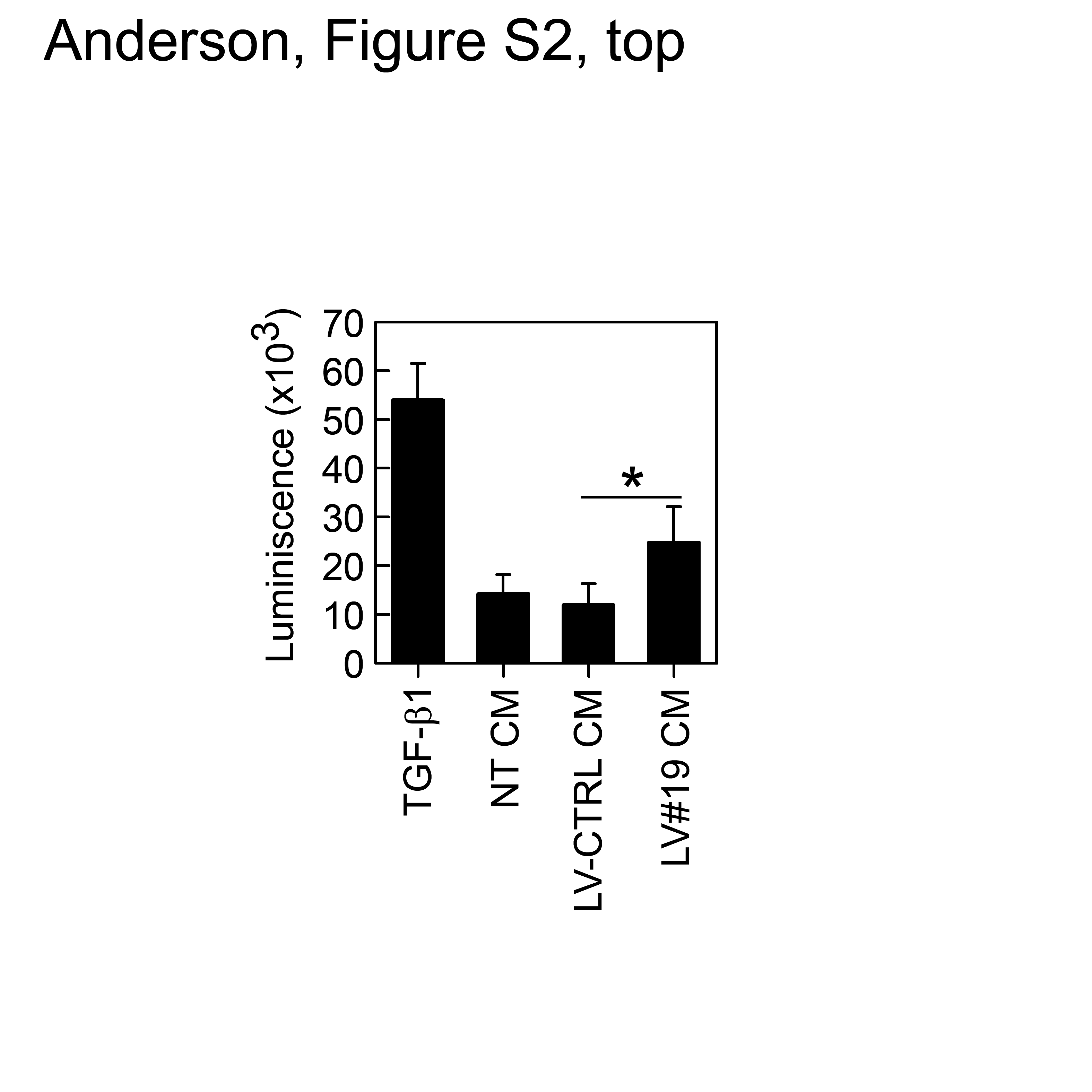

Supplement: Supplementary file 2 — Figure S2 Silencing of GARP in human ASCs increases their activation of TGF‐β. Recombinant TGF‐β1 (1 ng/mL) and conditioned medium (CM) from NT, LV‐CTRL and LV#19 ASCs were added to SBE‐HEK293 cells for 18 hours and luminescence was read on a Glomax Multi Detection System (Promega). Data are shown as mean(SD) of three independent experiments. * = P < 0.05. [file SCT3-9-636-s002.tif]
