# Supplementary material for: GARP is a key molecule for mesenchymal stromal cell responses to TGF‐β and fundamental to control mitochondrial ROS levels
Source: Stem Cells Transl Med. 2020 Feb 19;9(5):636–50. doi: 10.1002/sctm.19-0372 (PMC7180295; doi:10.1002/sctm.19-0372)
Supplement: Supplementary file 3 — Table S1 Genes down‐ and upregulated in LV#18/LV#19 vs NT/LV‐CTRL ASCs. [file SCT3-9-636-s003.docx]

SUPPLEMENTARY TABLE 1

| Genes downregulated in LV#18/LV#19 vs. NT/CTRL | | | | | | | | | | | | | | |
| --- | --- | --- | --- | --- | --- | --- | --- | --- | --- | --- | --- | --- | --- | --- |
| Genes | NT:19 | NT:23 | NT:27 | CTRL:19 | CTRL:23 | CTRL:27 | LV#18:19 | LV#18:23 | LV#18:27 | LV#19:19 | LV#19:23 | LV#19:27 | logFC | adj.P.Val |
| KRT81 | 7,76 | 7,29 | 9,06 | 8,36 | 7,63 | 9,20 | 5,54 | 4,87 | 7,26 | 5,15 | 4,53 | 6,72 | -2,54 | 0,008 |
| IL6 | 11,49 | 11,50 | 9,99 | 11,27 | 8,16 | 10,03 | 8,25 | 8,81 | 7,95 | 8,98 | 7,22 | 6,89 | -2,39 | 0,018 |
| LRRC32 | 10,21 | 10,53 | 10,51 | 10,81 | 10,47 | 10,58 | 9,25 | 8,43 | 9,12 | 8,72 | 7,39 | 8,57 | -1,94 | 0,001 |
| CH25H | 8,81 | 7,52 | 8,27 | 9,15 | 6,96 | 8,35 | 6,51 | 5,35 | 4,76 | 7,46 | 7,16 | 6,68 | -1,86 | 0,028 |
| KIF20A | 8,20 | 9,03 | 8,69 | 7,69 | 8,34 | 8,27 | 6,48 | 6,06 | 5,62 | 7,14 | 6,11 | 7,74 | -1,85 | 0,006 |
| CEP55 | 9,04 | 9,51 | 9,41 | 8,24 | 9,04 | 8,89 | 7,07 | 6,37 | 7,10 | 7,88 | 6,32 | 8,32 | -1,85 | 0,006 |
| VIL2 | 9,65 | 10,16 | 10,19 | 9,31 | 10,25 | 10,26 | 7,15 | 6,35 | 7,65 | 9,14 | 8,63 | 9,86 | -1,84 | 0,034 |
| CCNB1 | 8,52 | 9,01 | 8,88 | 7,75 | 8,54 | 8,54 | 6,57 | 6,19 | 6,45 | 7,33 | 6,17 | 7,95 | -1,76 | 0,005 |
| CDKN3 | 8,68 | 9,34 | 9,08 | 7,73 | 8,10 | 8,52 | 6,59 | 5,84 | 6,49 | 7,49 | 6,40 | 8,11 | -1,76 | 0,012 |
| ALDOC | 8,41 | 7,76 | 7,69 | 9,07 | 8,24 | 8,10 | 6,64 | 6,25 | 6,18 | 6,93 | 6,06 | 6,72 | -1,75 | 0,001 |
| EZR | 10,58 | 11,19 | 11,10 | 10,25 | 11,17 | 11,07 | 8,25 | 7,70 | 8,61 | 9,95 | 9,75 | 10,78 | -1,72 | 0,029 |
| CDC45L | 8,24 | 8,78 | 8,40 | 7,65 | 7,88 | 7,76 | 6,38 | 5,73 | 6,14 | 7,14 | 5,62 | 7,43 | -1,71 | 0,007 |
| CDC20 | 10,53 | 11,06 | 10,91 | 10,15 | 10,69 | 10,60 | 9,13 | 8,08 | 8,59 | 9,76 | 8,11 | 10,15 | -1,69 | 0,009 |
| RHOB | 9,69 | 9,55 | 9,92 | 9,33 | 8,75 | 9,80 | 7,91 | 7,42 | 8,73 | 8,02 | 6,94 | 8,02 | -1,67 | 0,004 |
| KRT7 | 7,74 | 8,06 | 7,94 | 7,70 | 8,61 | 8,53 | 5,79 | 5,30 | 5,27 | 6,99 | 7,62 | 7,60 | -1,67 | 0,026 |
| CDCA3 | 7,68 | 8,59 | 8,21 | 7,00 | 7,99 | 7,74 | 6,02 | 4,92 | 5,77 | 6,84 | 6,01 | 7,74 | -1,65 | 0,021 |
| AURKB | 7,23 | 7,63 | 7,22 | 6,73 | 7,48 | 7,07 | 5,36 | 4,72 | 5,67 | 6,07 | 5,20 | 6,41 | -1,65 | 0,003 |
| ASPM | 8,79 | 9,14 | 9,06 | 8,24 | 8,80 | 8,68 | 6,83 | 6,22 | 6,90 | 7,85 | 6,62 | 8,42 | -1,65 | 0,009 |
| NUSAP1 | 8,42 | 9,16 | 8,89 | 8,10 | 8,61 | 8,50 | 7,14 | 6,29 | 6,62 | 7,48 | 6,29 | 8,09 | -1,63 | 0,007 |
| KLF2 | 11,05 | 10,77 | 11,29 | 10,92 | 10,30 | 11,26 | 9,07 | 8,24 | 9,82 | 9,85 | 8,80 | 10,20 | -1,60 | 0,008 |
| PRC1 | 9,77 | 10,51 | 10,26 | 9,31 | 10,21 | 10,12 | 8,21 | 7,48 | 7,87 | 9,23 | 8,17 | 9,62 | -1,60 | 0,012 |
| PBK | 8,03 | 8,58 | 8,72 | 7,71 | 8,45 | 8,64 | 6,54 | 6,08 | 6,24 | 7,32 | 6,32 | 8,07 | -1,59 | 0,009 |
| FANCI | 7,70 | 7,97 | 7,66 | 7,04 | 8,00 | 7,28 | 6,26 | 5,85 | 5,88 | 6,45 | 5,27 | 6,49 | -1,58 | 0,002 |
| CCNA2 | 9,16 | 9,56 | 9,25 | 8,39 | 9,02 | 8,88 | 7,21 | 6,65 | 6,81 | 8,40 | 7,37 | 8,41 | -1,57 | 0,009 |
| GINS2 | 8,31 | 8,90 | 8,33 | 7,80 | 8,02 | 7,82 | 6,78 | 5,58 | 6,40 | 7,37 | 5,86 | 7,79 | -1,56 | 0,014 |
| CCNB2 | 9,03 | 9,51 | 9,41 | 8,71 | 9,30 | 9,15 | 7,29 | 6,74 | 6,95 | 8,12 | 7,48 | 9,14 | -1,56 | 0,013 |
| DLGAP5 | 7,73 | 8,51 | 8,19 | 6,92 | 7,91 | 7,79 | 6,23 | 5,64 | 5,84 | 6,80 | 5,84 | 7,37 | -1,55 | 0,010 |
| CENPF | 7,86 | 8,47 | 8,18 | 7,07 | 7,72 | 7,66 | 6,47 | 6,00 | 5,80 | 6,50 | 5,79 | 7,16 | -1,54 | 0,005 |
| AK3L1 | 8,01 | 7,77 | 7,78 | 8,58 | 8,77 | 8,27 | 6,94 | 7,34 | 7,01 | 6,61 | 5,86 | 6,20 | -1,54 | 0,004 |
| BUB1 | 6,65 | 7,61 | 7,12 | 6,03 | 6,87 | 6,62 | 5,20 | 4,72 | 5,41 | 5,65 | 4,89 | 6,03 | -1,50 | 0,006 |
| CDC2 | 7,89 | 8,55 | 8,26 | 7,72 | 8,24 | 8,15 | 6,63 | 6,00 | 6,25 | 6,91 | 6,20 | 7,87 | -1,49 | 0,007 |
| NCAPG | 8,32 | 8,72 | 8,61 | 7,88 | 8,63 | 7,95 | 6,89 | 6,30 | 7,04 | 7,33 | 5,86 | 7,75 | -1,49 | 0,008 |
| NDC80 | 8,08 | 8,30 | 8,03 | 7,44 | 7,88 | 7,78 | 6,29 | 5,63 | 6,19 | 6,97 | 6,10 | 7,49 | -1,47 | 0,007 |
| TMPO | 6,57 | 6,99 | 6,84 | 5,62 | 6,35 | 6,50 | 5,02 | 4,37 | 4,96 | 5,45 | 4,69 | 5,59 | -1,46 | 0,005 |
| FAM83D | 7,28 | 7,62 | 7,63 | 6,55 | 7,50 | 7,24 | 5,48 | 5,28 | 5,53 | 6,40 | 5,53 | 6,85 | -1,46 | 0,008 |
| MLEC | 9,04 | 9,31 | 9,13 | 8,79 | 8,89 | 8,83 | 7,45 | 6,99 | 7,51 | 8,07 | 7,23 | 8,01 | -1,46 | 0,001 |
| ANLN | 8,32 | 9,16 | 8,67 | 7,75 | 8,99 | 8,39 | 6,93 | 6,11 | 6,63 | 7,66 | 6,78 | 8,43 | -1,45 | 0,021 |
| KIF11 | 7,24 | 7,59 | 7,44 | 6,59 | 7,07 | 7,15 | 5,62 | 4,59 | 5,32 | 6,39 | 5,48 | 6,97 | -1,45 | 0,016 |
| CENPA | 6,28 | 7,27 | 7,20 | 6,29 | 6,84 | 6,94 | 5,40 | 4,68 | 4,89 | 5,69 | 5,32 | 6,25 | -1,43 | 0,007 |
| KIAA0101 | 9,10 | 9,83 | 9,71 | 8,62 | 8,82 | 9,22 | 7,85 | 7,04 | 7,42 | 8,32 | 7,00 | 9,06 | -1,43 | 0,019 |
| BIRC5 | 6,91 | 7,96 | 7,51 | 6,53 | 7,13 | 7,48 | 5,85 | 4,94 | 5,60 | 6,37 | 5,68 | 6,52 | -1,43 | 0,009 |
| CHAF1B | 6,82 | 7,13 | 6,82 | 6,63 | 6,74 | 6,45 | 5,09 | 4,59 | 5,06 | 5,89 | 5,59 | 5,86 | -1,42 | 0,003 |
| KIF2C | 6,93 | 7,39 | 7,17 | 6,40 | 6,83 | 6,99 | 5,41 | 5,09 | 5,30 | 6,04 | 5,08 | 6,28 | -1,42 | 0,004 |
| MAD2L1 | 8,22 | 8,76 | 8,40 | 7,85 | 8,26 | 8,09 | 6,59 | 6,14 | 6,46 | 7,33 | 6,54 | 8,05 | -1,41 | 0,009 |
| CDCA5 | 8,49 | 9,00 | 8,58 | 8,11 | 8,72 | 8,40 | 7,25 | 6,92 | 6,97 | 7,54 | 6,20 | 7,97 | -1,41 | 0,006 |
| MT1F | 8,08 | 7,80 | 7,40 | 8,00 | 6,35 | 7,55 | 7,45 | 6,25 | 6,00 | 5,75 | 5,11 | 6,22 | -1,40 | 0,029 |
| EXO1 | 6,79 | 7,33 | 6,91 | 6,48 | 6,67 | 6,29 | 5,32 | 4,91 | 5,21 | 5,90 | 4,72 | 6,05 | -1,39 | 0,005 |
| TNFRSF11B | 9,50 | 9,23 | 8,99 | 8,85 | 8,56 | 9,13 | 7,87 | 7,20 | 7,69 | 7,95 | 7,29 | 7,93 | -1,39 | 0,001 |
| KIF20B | 7,82 | 8,44 | 8,31 | 7,44 | 8,26 | 7,77 | 6,47 | 6,28 | 6,39 | 7,04 | 5,99 | 7,55 | -1,39 | 0,007 |
| TK1 | 8,91 | 8,82 | 8,89 | 8,11 | 7,87 | 8,48 | 7,06 | 5,74 | 6,44 | 8,14 | 6,90 | 8,48 | -1,39 | 0,049 |
| UHRF1 | 9,25 | 9,70 | 9,49 | 8,81 | 9,12 | 9,03 | 8,17 | 7,68 | 8,10 | 8,36 | 6,59 | 8,20 | -1,38 | 0,009 |
| RCN2 | 9,85 | 10,07 | 9,85 | 10,06 | 9,98 | 9,75 | 9,14 | 9,06 | 8,90 | 8,26 | 7,84 | 8,22 | -1,36 | 0,003 |
| MXRA5 | 10,13 | 10,35 | 10,14 | 10,48 | 10,03 | 10,09 | 9,82 | 9,88 | 8,47 | 9,09 | 7,55 | 8,27 | -1,36 | 0,025 |
| SLC2A1 | 10,20 | 9,87 | 9,69 | 10,90 | 10,56 | 10,19 | 9,72 | 9,07 | 8,53 | 9,11 | 8,34 | 8,56 | -1,35 | 0,007 |
| TACC3 | 6,79 | 7,47 | 7,15 | 6,26 | 7,12 | 6,93 | 5,57 | 4,97 | 5,48 | 6,30 | 5,13 | 6,33 | -1,32 | 0,009 |
| CENPE | 7,14 | 7,53 | 7,50 | 6,47 | 7,13 | 7,01 | 5,85 | 5,56 | 5,52 | 6,14 | 5,39 | 6,41 | -1,32 | 0,004 |
| HMMR | 8,27 | 8,90 | 8,62 | 7,90 | 8,54 | 8,29 | 7,14 | 6,63 | 6,75 | 7,46 | 6,79 | 7,89 | -1,31 | 0,005 |
| TOP2A | 8,38 | 8,83 | 8,87 | 8,18 | 8,65 | 8,65 | 7,53 | 6,49 | 6,87 | 7,72 | 6,83 | 8,26 | -1,31 | 0,010 |
| PGK1 | 10,82 | 10,48 | 10,68 | 11,36 | 11,35 | 10,90 | 9,96 | 10,04 | 9,89 | 9,67 | 8,83 | 9,36 | -1,31 | 0,005 |
| MUC1 | 7,12 | 7,24 | 7,41 | 7,89 | 7,67 | 7,63 | 5,57 | 5,68 | 5,43 | 7,05 | 6,70 | 6,71 | -1,30 | 0,013 |
| CDT1 | 6,91 | 6,97 | 7,01 | 6,42 | 6,50 | 6,44 | 5,11 | 4,61 | 5,60 | 5,79 | 4,75 | 6,59 | -1,30 | 0,015 |
| FLJ14213 | 6,22 | 6,43 | 6,45 | 6,67 | 6,99 | 6,61 | 5,87 | 5,25 | 5,63 | 5,08 | 4,96 | 4,81 | -1,29 | 0,002 |
| POLQ | 6,87 | 6,95 | 6,89 | 6,36 | 6,99 | 6,71 | 4,68 | 4,78 | 5,42 | 6,13 | 5,56 | 6,46 | -1,29 | 0,012 |
| TRIP13 | 8,59 | 8,99 | 8,98 | 8,07 | 8,45 | 8,34 | 7,33 | 6,68 | 6,84 | 7,80 | 6,90 | 8,15 | -1,29 | 0,009 |
| DEPDC1 | 6,50 | 6,88 | 6,51 | 5,89 | 6,60 | 6,58 | 5,14 | 4,74 | 4,93 | 5,48 | 4,88 | 6,04 | -1,29 | 0,005 |
| MCM2 | 6,70 | 7,33 | 6,94 | 6,20 | 6,25 | 6,01 | 5,01 | 4,81 | 5,17 | 5,85 | 5,06 | 5,83 | -1,29 | 0,008 |
| NEK2 | 6,66 | 7,28 | 6,94 | 5,96 | 6,71 | 6,77 | 5,24 | 4,41 | 5,26 | 6,01 | 5,24 | 6,45 | -1,29 | 0,019 |
| CCDC85A | 7,17 | 6,81 | 7,46 | 6,67 | 7,53 | 7,62 | 5,68 | 5,02 | 5,36 | 6,76 | 6,18 | 6,54 | -1,29 | 0,016 |
| MCM6 | 9,95 | 9,84 | 9,76 | 9,25 | 9,15 | 9,41 | 7,98 | 6,99 | 7,68 | 9,30 | 8,51 | 9,18 | -1,29 | 0,035 |
| LOC646993 | 7,10 | 7,48 | 7,33 | 6,67 | 7,17 | 7,50 | 6,16 | 6,47 | 5,83 | 5,66 | 4,99 | 6,44 | -1,28 | 0,007 |
| OIP5 | 7,07 | 7,49 | 7,18 | 6,43 | 7,03 | 7,03 | 5,77 | 5,28 | 5,44 | 6,02 | 5,40 | 6,65 | -1,28 | 0,006 |
| POLA2 | 7,57 | 7,99 | 7,70 | 7,48 | 7,65 | 7,25 | 6,46 | 5,93 | 6,37 | 6,46 | 5,77 | 6,98 | -1,28 | 0,003 |
| HIST1H4C | 12,21 | 12,94 | 12,60 | 11,88 | 12,48 | 12,28 | 10,82 | 10,23 | 10,79 | 12,01 | 10,88 | 12,01 | -1,28 | 0,018 |
| TYMS | 9,97 | 10,11 | 10,01 | 9,29 | 9,34 | 9,72 | 8,65 | 7,66 | 7,94 | 9,15 | 7,82 | 9,58 | -1,28 | 0,023 |
| FOXM1 | 7,07 | 7,73 | 7,39 | 6,41 | 6,80 | 6,80 | 5,65 | 5,05 | 5,26 | 6,36 | 5,63 | 6,64 | -1,27 | 0,016 |
| FAM26F | 7,20 | 7,05 | 6,94 | 7,64 | 7,17 | 7,10 | 5,65 | 5,76 | 5,59 | 6,29 | 5,77 | 6,41 | -1,27 | 0,001 |
| POLE2 | 7,33 | 7,73 | 7,40 | 6,86 | 6,95 | 6,79 | 5,71 | 5,55 | 5,78 | 6,44 | 5,32 | 6,65 | -1,27 | 0,007 |
| KCTD12 | 6,72 | 7,14 | 6,94 | 6,68 | 6,79 | 6,79 | 5,48 | 5,64 | 5,98 | 5,59 | 5,22 | 5,54 | -1,27 | 0,000 |
| BNIP3 | 12,04 | 11,73 | 11,64 | 12,13 | 11,98 | 12,01 | 10,89 | 10,72 | 10,42 | 11,04 | 10,45 | 10,38 | -1,27 | 0,001 |
| CENPM | 6,90 | 7,68 | 7,09 | 6,48 | 6,88 | 6,86 | 5,96 | 5,12 | 5,07 | 6,21 | 5,24 | 6,69 | -1,27 | 0,016 |
| PLK1 | 6,52 | 7,00 | 6,93 | 6,10 | 6,63 | 6,54 | 5,51 | 4,90 | 5,00 | 5,62 | 5,01 | 6,15 | -1,26 | 0,006 |
| NIPSNAP1 | 7,62 | 7,22 | 7,20 | 7,76 | 7,39 | 7,46 | 6,08 | 6,16 | 5,76 | 6,63 | 6,04 | 6,45 | -1,26 | 0,001 |
| LDB2 | 9,12 | 9,55 | 9,97 | 8,87 | 9,16 | 9,87 | 8,44 | 7,14 | 8,77 | 8,62 | 7,30 | 8,75 | -1,25 | 0,026 |
| HJURP | 6,47 | 7,05 | 6,86 | 6,25 | 6,61 | 6,67 | 5,08 | 4,83 | 5,37 | 5,71 | 5,19 | 6,23 | -1,25 | 0,005 |
| FSTL3 | 6,62 | 6,78 | 6,98 | 6,76 | 7,39 | 7,31 | 5,36 | 5,96 | 6,61 | 5,51 | 5,48 | 5,42 | -1,25 | 0,005 |
| PRR11 | 5,96 | 6,86 | 6,64 | 5,82 | 6,43 | 6,62 | 5,32 | 4,71 | 4,89 | 5,31 | 4,73 | 5,93 | -1,24 | 0,008 |
| LOC653110 | 5,48 | 5,87 | 5,63 | 5,60 | 6,45 | 6,18 | 4,37 | 4,41 | 4,82 | 4,56 | 4,83 | 4,80 | -1,24 | 0,001 |
| LOC644774 | 10,74 | 10,33 | 10,44 | 11,33 | 11,09 | 10,82 | 10,20 | 10,09 | 9,69 | 9,41 | 8,79 | 9,14 | -1,23 | 0,009 |
| SPC24 | 8,52 | 8,93 | 8,63 | 7,88 | 8,29 | 8,40 | 7,17 | 6,73 | 6,81 | 7,71 | 6,63 | 8,20 | -1,23 | 0,013 |
| TTK | 7,09 | 7,44 | 7,32 | 6,59 | 7,11 | 6,98 | 5,49 | 5,57 | 5,49 | 6,33 | 5,47 | 6,79 | -1,23 | 0,008 |
| HMGB2 | 9,27 | 9,53 | 9,09 | 8,54 | 8,82 | 8,90 | 7,64 | 7,25 | 7,25 | 8,35 | 7,51 | 8,77 | -1,23 | 0,013 |
| TPX2 | 7,49 | 8,08 | 7,83 | 6,96 | 7,58 | 7,55 | 6,29 | 5,35 | 6,00 | 6,86 | 6,04 | 7,57 | -1,23 | 0,027 |
| CKAP2L | 7,14 | 7,91 | 7,56 | 6,81 | 7,25 | 7,07 | 6,10 | 6,29 | 6,01 | 6,35 | 4,85 | 6,77 | -1,23 | 0,016 |
| C13orf3 | 6,48 | 6,48 | 6,33 | 5,71 | 6,27 | 5,88 | 4,74 | 4,77 | 5,13 | 5,37 | 4,65 | 5,18 | -1,22 | 0,002 |
| ASF1B | 6,10 | 6,99 | 6,06 | 5,96 | 6,49 | 6,22 | 5,09 | 4,43 | 5,12 | 5,59 | 4,83 | 5,46 | -1,22 | 0,006 |
| MXRA7 | 7,65 | 7,47 | 7,09 | 7,79 | 8,03 | 7,50 | 6,64 | 7,04 | 6,84 | 5,80 | 5,65 | 6,29 | -1,21 | 0,009 |
| SCARA3 | 7,36 | 7,54 | 7,79 | 7,40 | 7,16 | 7,67 | 6,62 | 5,72 | 6,50 | 6,50 | 6,07 | 6,27 | -1,21 | 0,001 |
| CDCA8 | 6,82 | 7,53 | 7,31 | 6,45 | 7,39 | 6,97 | 5,79 | 5,05 | 5,70 | 6,38 | 5,48 | 6,91 | -1,20 | 0,020 |
| DEK | 10,51 | 10,74 | 10,39 | 9,98 | 10,24 | 10,01 | 9,27 | 8,84 | 9,08 | 9,55 | 8,49 | 9,46 | -1,19 | 0,004 |
| SLC7A5 | 9,31 | 9,35 | 9,34 | 9,73 | 10,14 | 9,95 | 8,78 | 8,82 | 8,24 | 8,07 | 8,54 | 8,22 | -1,19 | 0,003 |
| KCTD10 | 8,24 | 9,38 | 8,24 | 8,23 | 9,24 | 8,29 | 7,15 | 8,45 | 7,28 | 7,03 | 7,32 | 7,26 | -1,19 | 0,019 |
| ZWINT | 6,40 | 6,88 | 6,69 | 5,86 | 6,20 | 6,19 | 4,61 | 4,46 | 5,10 | 5,56 | 5,06 | 6,32 | -1,19 | 0,020 |
| NDUFA4L2 | 6,44 | 5,48 | 5,98 | 7,49 | 6,32 | 5,76 | 4,75 | 4,75 | 5,19 | 5,38 | 4,92 | 5,41 | -1,18 | 0,020 |
| PLK4 | 7,00 | 7,69 | 7,32 | 6,64 | 7,83 | 7,04 | 6,17 | 6,12 | 5,75 | 6,15 | 5,75 | 6,50 | -1,18 | 0,005 |
| DACT1 | 8,50 | 9,02 | 7,67 | 8,34 | 8,61 | 8,14 | 7,24 | 7,68 | 5,90 | 7,45 | 7,81 | 7,16 | -1,18 | 0,028 |
| SYNM | 6,07 | 6,60 | 5,70 | 5,89 | 6,89 | 6,28 | 4,79 | 4,94 | 4,81 | 5,31 | 5,62 | 4,91 | -1,17 | 0,006 |
| C11orf24 | 7,68 | 8,08 | 7,97 | 7,96 | 8,00 | 8,02 | 7,18 | 7,10 | 7,47 | 6,53 | 6,01 | 6,40 | -1,17 | 0,007 |
| CKAP2 | 7,58 | 8,48 | 7,95 | 7,10 | 8,14 | 7,96 | 6,26 | 6,24 | 6,23 | 7,16 | 6,71 | 7,63 | -1,16 | 0,019 |
| KIFC1 | 6,37 | 6,77 | 6,66 | 5,62 | 6,07 | 6,03 | 5,15 | 4,48 | 4,61 | 5,44 | 4,77 | 6,09 | -1,16 | 0,019 |
| SULF1 | 10,94 | 11,57 | 11,46 | 10,75 | 10,61 | 11,36 | 9,59 | 10,15 | 10,08 | 9,93 | 9,79 | 10,19 | -1,16 | 0,003 |
| LOC643287 | 10,01 | 10,37 | 10,06 | 9,79 | 9,43 | 9,75 | 9,54 | 9,12 | 9,35 | 8,45 | 7,50 | 8,48 | -1,16 | 0,029 |
| NNMT | 11,05 | 11,30 | 11,00 | 11,28 | 10,63 | 11,29 | 10,13 | 9,97 | 8,93 | 10,18 | 10,63 | 9,85 | -1,14 | 0,010 |
| NUF2 | 6,38 | 7,51 | 7,00 | 6,09 | 6,91 | 6,39 | 5,45 | 5,18 | 5,26 | 5,93 | 5,19 | 6,42 | -1,14 | 0,019 |
| AURKA | 8,14 | 9,08 | 8,67 | 7,72 | 8,69 | 8,55 | 7,10 | 6,56 | 6,99 | 7,79 | 7,16 | 8,43 | -1,14 | 0,031 |
| RAD51AP1 | 7,33 | 7,84 | 7,53 | 6,57 | 6,85 | 7,09 | 6,26 | 6,13 | 6,31 | 6,36 | 5,06 | 6,31 | -1,13 | 0,015 |
| LOC728873 | 10,44 | 10,77 | 10,51 | 10,13 | 9,95 | 10,21 | 9,96 | 9,77 | 9,71 | 9,07 | 7,90 | 8,86 | -1,13 | 0,034 |
| WDR51A | 8,32 | 8,88 | 8,44 | 8,12 | 8,56 | 8,39 | 7,35 | 7,26 | 6,99 | 7,41 | 6,81 | 8,16 | -1,12 | 0,006 |
| NCAPG2 | 6,24 | 5,94 | 6,04 | 5,84 | 5,69 | 6,01 | 4,54 | 4,84 | 4,49 | 5,07 | 4,87 | 5,21 | -1,12 | 0,001 |
| KIF14 | 6,78 | 7,32 | 6,88 | 6,24 | 6,88 | 6,77 | 5,79 | 5,52 | 5,52 | 5,96 | 5,16 | 6,19 | -1,12 | 0,005 |
| RASSF7 | 6,59 | 6,34 | 6,26 | 6,82 | 7,73 | 6,87 | 5,12 | 5,02 | 4,97 | 6,40 | 6,04 | 6,38 | -1,12 | 0,045 |
| EVL | 9,92 | 9,94 | 9,86 | 10,25 | 9,98 | 9,98 | 9,41 | 9,49 | 9,08 | 8,66 | 8,16 | 8,44 | -1,12 | 0,008 |
| PFKFB3 | 7,78 | 8,03 | 7,73 | 8,25 | 8,40 | 8,13 | 7,19 | 7,03 | 7,15 | 7,10 | 6,25 | 6,91 | -1,12 | 0,003 |
| CKAP4 | 11,74 | 12,11 | 12,04 | 11,67 | 11,96 | 11,83 | 11,29 | 11,69 | 11,51 | 10,43 | 9,51 | 10,25 | -1,11 | 0,045 |
| CCNE2 | 5,96 | 6,41 | 6,31 | 5,51 | 6,44 | 5,73 | 4,63 | 4,55 | 5,16 | 5,68 | 4,69 | 4,98 | -1,11 | 0,009 |
| EFEMP1 | 9,49 | 10,42 | 9,32 | 9,42 | 10,28 | 9,68 | 8,71 | 9,46 | 8,46 | 8,40 | 8,51 | 8,40 | -1,11 | 0,012 |
| RFC3 | 6,13 | 6,59 | 6,39 | 5,72 | 6,18 | 5,86 | 4,90 | 4,60 | 4,70 | 5,36 | 4,82 | 5,86 | -1,11 | 0,009 |
| TRIM6 | 6,96 | 7,44 | 6,95 | 7,20 | 7,35 | 7,21 | 6,00 | 5,42 | 5,55 | 6,78 | 5,91 | 6,82 | -1,11 | 0,012 |
| PGM2 | 7,10 | 7,00 | 7,31 | 7,27 | 6,88 | 7,19 | 5,21 | 5,03 | 5,50 | 6,91 | 6,60 | 6,86 | -1,11 | 0,048 |
| KCNJ8 | 7,01 | 7,36 | 6,52 | 6,83 | 7,20 | 6,67 | 5,61 | 5,29 | 5,22 | 6,52 | 6,18 | 6,12 | -1,11 | 0,012 |
| C6orf173 | 9,19 | 9,95 | 9,45 | 8,45 | 9,08 | 9,07 | 7,98 | 7,82 | 7,90 | 8,51 | 7,08 | 9,27 | -1,11 | 0,049 |
| TBC1D2B | 7,39 | 7,39 | 7,52 | 8,15 | 7,58 | 7,67 | 6,76 | 5,42 | 6,72 | 6,75 | 6,73 | 6,68 | -1,10 | 0,011 |
| UBE2C | 7,28 | 7,61 | 7,35 | 6,86 | 7,31 | 7,62 | 6,15 | 5,53 | 5,94 | 6,63 | 6,07 | 7,07 | -1,10 | 0,011 |
| RUNX1 | 7,85 | 8,28 | 8,05 | 8,02 | 7,78 | 7,79 | 7,17 | 6,64 | 6,81 | 6,94 | 6,89 | 6,76 | -1,09 | 0,001 |
| KIAA1618 | 7,88 | 7,47 | 7,59 | 7,60 | 7,24 | 7,56 | 7,15 | 6,20 | 7,00 | 6,67 | 5,29 | 6,49 | -1,09 | 0,020 |
| NCAPD2 | 7,59 | 8,57 | 7,98 | 7,28 | 7,86 | 7,58 | 6,47 | 6,12 | 6,07 | 7,37 | 7,03 | 7,27 | -1,09 | 0,023 |
| BUB1B | 6,34 | 6,74 | 6,58 | 5,84 | 6,23 | 6,37 | 5,45 | 5,01 | 4,72 | 5,71 | 5,12 | 5,60 | -1,08 | 0,005 |
| CENPK | 7,23 | 8,11 | 7,23 | 6,91 | 7,69 | 7,31 | 6,11 | 6,10 | 6,02 | 6,38 | 6,28 | 7,10 | -1,08 | 0,010 |
| CRISPLD2 | 9,32 | 9,53 | 9,02 | 9,02 | 8,47 | 9,11 | 8,35 | 8,69 | 7,40 | 8,26 | 8,05 | 7,23 | -1,08 | 0,018 |
| TROAP | 6,76 | 6,96 | 6,98 | 6,26 | 6,62 | 6,39 | 5,59 | 5,33 | 5,05 | 5,74 | 5,41 | 6,36 | -1,08 | 0,008 |
| LOC731314 | 8,45 | 9,08 | 8,58 | 8,05 | 8,67 | 8,41 | 7,48 | 6,90 | 7,05 | 7,85 | 7,41 | 8,10 | -1,08 | 0,009 |
| PSRC1 | 6,71 | 7,02 | 6,88 | 6,46 | 6,67 | 6,51 | 5,55 | 4,93 | 4,91 | 6,30 | 5,35 | 6,76 | -1,07 | 0,033 |
| LOC648822 | 8,98 | 9,47 | 9,29 | 8,60 | 8,64 | 8,89 | 7,95 | 7,50 | 7,35 | 8,10 | 7,82 | 8,77 | -1,07 | 0,013 |
| RRM2 | 6,22 | 7,02 | 6,44 | 5,65 | 6,43 | 6,03 | 5,11 | 5,27 | 5,37 | 5,24 | 4,94 | 5,52 | -1,06 | 0,007 |
| LOC440132 | 5,45 | 5,82 | 5,72 | 6,35 | 6,49 | 6,51 | 4,54 | 4,85 | 4,53 | 5,35 | 5,25 | 5,48 | -1,06 | 0,014 |
| CCDC58 | 6,14 | 6,68 | 6,44 | 5,76 | 6,04 | 5,83 | 4,83 | 4,68 | 4,76 | 5,86 | 4,70 | 5,73 | -1,06 | 0,018 |
| C16orf30 | 7,39 | 6,82 | 6,97 | 7,53 | 6,76 | 7,20 | 5,54 | 6,70 | 6,66 | 5,93 | 5,53 | 5,96 | -1,06 | 0,013 |
| ADARB1 | 7,04 | 7,30 | 7,25 | 7,08 | 7,15 | 7,71 | 5,80 | 5,53 | 5,87 | 6,67 | 6,76 | 6,60 | -1,05 | 0,012 |
| RACGAP1 | 6,90 | 7,49 | 7,11 | 6,29 | 7,09 | 6,64 | 5,55 | 5,07 | 5,83 | 6,26 | 5,84 | 6,68 | -1,05 | 0,023 |
| MELK | 8,40 | 8,80 | 8,61 | 8,03 | 8,46 | 8,20 | 7,03 | 6,47 | 7,10 | 7,95 | 7,37 | 8,33 | -1,04 | 0,028 |
| RNASEH2A | 7,28 | 7,99 | 7,61 | 6,81 | 7,41 | 7,36 | 6,43 | 6,16 | 6,29 | 6,42 | 6,15 | 6,77 | -1,04 | 0,005 |
| NHS | 6,02 | 6,05 | 6,04 | 6,20 | 5,82 | 6,40 | 4,90 | 4,71 | 4,69 | 5,57 | 5,06 | 5,36 | -1,04 | 0,003 |
| GAL3ST4 | 7,64 | 6,97 | 7,55 | 7,87 | 6,95 | 7,40 | 6,49 | 6,02 | 6,04 | 6,89 | 5,85 | 6,86 | -1,04 | 0,012 |
| PAQR4 | 7,32 | 7,58 | 7,28 | 7,08 | 6,91 | 7,05 | 5,56 | 5,67 | 6,50 | 6,57 | 5,66 | 7,06 | -1,04 | 0,020 |
| EIF2C1 | 6,38 | 6,25 | 6,37 | 5,93 | 5,47 | 5,94 | 4,70 | 4,84 | 4,72 | 5,28 | 5,09 | 5,51 | -1,03 | 0,006 |
| SLBP | 8,95 | 9,14 | 9,01 | 8,85 | 9,23 | 8,88 | 7,65 | 7,37 | 7,57 | 8,49 | 8,25 | 8,52 | -1,03 | 0,008 |
| C10orf26 | 6,89 | 6,86 | 6,60 | 7,17 | 7,01 | 7,06 | 6,42 | 6,06 | 6,41 | 5,77 | 5,42 | 5,33 | -1,03 | 0,008 |
| LOC728026 | 10,68 | 10,87 | 10,79 | 10,41 | 10,26 | 10,45 | 10,09 | 9,96 | 10,23 | 9,35 | 8,18 | 9,48 | -1,03 | 0,043 |
| LOC651816 | 8,77 | 9,64 | 9,15 | 8,55 | 9,22 | 9,02 | 8,07 | 7,89 | 7,79 | 8,08 | 7,87 | 8,47 | -1,03 | 0,005 |
| FBXO5 | 7,20 | 7,53 | 7,19 | 6,65 | 7,01 | 6,78 | 5,99 | 5,40 | 5,97 | 6,33 | 5,77 | 6,78 | -1,02 | 0,012 |
| LOC100129034 | 6,71 | 6,84 | 6,63 | 7,23 | 7,30 | 7,00 | 6,27 | 5,92 | 5,95 | 5,94 | 5,59 | 5,92 | -1,02 | 0,002 |
| BHLHB2 | 9,77 | 9,60 | 10,00 | 10,45 | 10,42 | 10,50 | 9,52 | 9,63 | 9,52 | 8,71 | 8,40 | 8,88 | -1,02 | 0,019 |
| CENPN | 8,45 | 9,46 | 8,71 | 7,89 | 8,48 | 8,37 | 7,03 | 7,40 | 7,28 | 7,66 | 7,56 | 8,36 | -1,01 | 0,025 |
| PTRF | 13,24 | 13,21 | 13,29 | 13,07 | 13,19 | 13,32 | 11,59 | 11,34 | 11,83 | 12,89 | 12,78 | 12,84 | -1,01 | 0,029 |
| FAM72D | 6,96 | 7,49 | 7,17 | 6,70 | 6,97 | 6,98 | 5,93 | 5,35 | 5,89 | 6,38 | 5,90 | 6,80 | -1,01 | 0,012 |
| C7orf68 | 8,02 | 7,94 | 7,54 | 9,22 | 8,11 | 7,82 | 7,50 | 7,41 | 6,63 | 7,20 | 6,88 | 6,99 | -1,00 | 0,023 |
| ANXA8 | 5,48 | 5,35 | 4,70 | 5,47 | 5,80 | 5,60 | 4,41 | 4,28 | 4,55 | 4,50 | 4,37 | 4,27 | -1,00 | 0,003 |
| CCNF | 7,78 | 8,31 | 7,94 | 7,45 | 8,21 | 7,85 | 6,96 | 6,21 | 6,67 | 7,39 | 6,79 | 7,54 | -1,00 | 0,014 |
| C15orf23 | 7,12 | 7,97 | 7,61 | 6,77 | 7,41 | 7,35 | 6,50 | 6,44 | 6,54 | 6,20 | 5,86 | 6,72 | -1,00 | 0,009 |
| LOC100132060 | 7,83 | 7,79 | 7,77 | 7,95 | 7,99 | 7,72 | 7,18 | 7,59 | 7,11 | 6,39 | 6,46 | 6,35 | -0,99 | 0,010 |
| ALS2CR4 | 9,58 | 9,47 | 9,64 | 9,64 | 9,56 | 9,68 | 9,07 | 8,80 | 8,56 | 8,57 | 8,08 | 8,55 | -0,99 | 0,002 |
| BARD1 | 6,14 | 6,70 | 6,17 | 5,59 | 6,46 | 5,47 | 4,59 | 4,42 | 4,90 | 5,48 | 5,56 | 5,67 | -0,99 | 0,035 |
| PTK7 | 7,49 | 6,85 | 7,63 | 8,05 | 6,83 | 7,74 | 7,06 | 6,24 | 6,37 | 6,60 | 6,02 | 6,38 | -0,99 | 0,018 |
| MSTO1 | 8,48 | 8,26 | 8,19 | 8,33 | 7,83 | 7,80 | 6,98 | 7,04 | 6,85 | 7,51 | 7,22 | 7,39 | -0,98 | 0,003 |
| CHSY1 | 10,28 | 10,09 | 10,12 | 10,12 | 9,73 | 10,11 | 9,32 | 9,35 | 9,15 | 9,07 | 8,60 | 9,05 | -0,98 | 0,002 |
| GTSE1 | 6,46 | 6,76 | 6,81 | 6,06 | 6,70 | 6,56 | 5,61 | 4,95 | 5,43 | 5,99 | 5,09 | 6,39 | -0,98 | 0,018 |
| CKS2 | 10,11 | 10,85 | 10,39 | 9,60 | 10,46 | 10,07 | 9,36 | 8,90 | 9,14 | 9,57 | 8,48 | 10,15 | -0,98 | 0,035 |
| DTL | 5,88 | 6,22 | 6,20 | 5,62 | 5,38 | 5,48 | 4,60 | 4,50 | 4,78 | 5,32 | 4,55 | 5,16 | -0,98 | 0,009 |
| LOC729666 | 6,77 | 6,51 | 6,51 | 7,10 | 7,06 | 6,83 | 6,16 | 6,25 | 6,54 | 5,30 | 5,01 | 5,63 | -0,98 | 0,024 |
| LOC100132888 | 6,06 | 6,00 | 6,06 | 6,98 | 6,94 | 6,56 | 5,59 | 6,23 | 5,75 | 5,24 | 4,97 | 4,95 | -0,98 | 0,028 |
| PHF19 | 6,73 | 7,00 | 7,07 | 6,46 | 6,85 | 6,54 | 6,06 | 5,25 | 5,56 | 5,89 | 5,36 | 6,67 | -0,98 | 0,016 |
| SNORA73B | 6,09 | 5,96 | 6,16 | 5,38 | 5,59 | 5,66 | 4,67 | 4,95 | 5,44 | 4,57 | 4,66 | 4,71 | -0,97 | 0,006 |
| C13orf15 | 6,23 | 5,97 | 6,08 | 6,57 | 5,41 | 6,16 | 4,66 | 4,87 | 5,64 | 5,55 | 4,91 | 4,94 | -0,97 | 0,013 |
| C4orf43 | 6,31 | 6,34 | 6,29 | 6,28 | 6,09 | 6,14 | 4,68 | 4,80 | 5,19 | 5,92 | 5,32 | 5,73 | -0,97 | 0,009 |
| NAV1 | 8,09 | 8,68 | 8,59 | 8,17 | 8,56 | 8,50 | 7,43 | 7,74 | 7,41 | 7,35 | 7,52 | 7,34 | -0,97 | 0,001 |
| SLC38A5 | 6,52 | 6,83 | 6,96 | 7,59 | 7,32 | 7,25 | 6,08 | 5,45 | 6,26 | 6,44 | 5,79 | 6,62 | -0,97 | 0,015 |
| FAM113B | 6,63 | 6,68 | 6,37 | 6,56 | 6,73 | 6,71 | 4,72 | 5,49 | 5,16 | 6,27 | 6,24 | 6,00 | -0,97 | 0,023 |
| CTDSP2 | 9,84 | 9,76 | 9,93 | 10,09 | 10,10 | 10,17 | 8,61 | 8,38 | 8,56 | 9,65 | 9,60 | 9,30 | -0,97 | 0,017 |
| SNORD80 | 7,61 | 7,44 | 7,24 | 6,82 | 6,62 | 6,87 | 6,48 | 5,70 | 6,56 | 6,29 | 5,39 | 6,38 | -0,97 | 0,020 |
| SLC12A8 | 7,89 | 8,28 | 7,70 | 8,28 | 9,14 | 8,33 | 7,11 | 8,09 | 6,56 | 7,34 | 7,79 | 6,93 | -0,96 | 0,046 |
| LOC730746 | 8,81 | 8,91 | 8,81 | 8,37 | 8,10 | 8,40 | 7,87 | 7,45 | 7,67 | 7,41 | 7,27 | 8,00 | -0,96 | 0,006 |
| C11orf82 | 6,52 | 7,22 | 6,52 | 5,79 | 6,84 | 6,42 | 5,60 | 5,60 | 5,59 | 5,65 | 5,28 | 5,88 | -0,95 | 0,011 |
| PDXP | 8,41 | 9,05 | 8,48 | 8,53 | 8,94 | 8,58 | 8,01 | 7,89 | 7,85 | 7,67 | 7,19 | 7,73 | -0,94 | 0,004 |
| ENPP1 | 7,31 | 7,84 | 7,49 | 6,75 | 6,88 | 6,91 | 5,91 | 5,85 | 6,21 | 6,77 | 6,26 | 6,52 | -0,94 | 0,014 |
| SNORD104 | 6,23 | 6,39 | 6,13 | 6,01 | 5,89 | 6,31 | 5,09 | 4,58 | 5,70 | 5,39 | 5,14 | 5,43 | -0,94 | 0,006 |
| TCF4 | 8,86 | 8,42 | 8,70 | 8,83 | 8,16 | 8,63 | 7,80 | 7,71 | 7,71 | 7,83 | 7,41 | 7,50 | -0,94 | 0,002 |
| TACC1 | 9,63 | 9,95 | 10,15 | 9,73 | 9,81 | 9,98 | 9,15 | 8,64 | 9,13 | 9,07 | 8,41 | 9,22 | -0,94 | 0,004 |
| GPT2 | 8,22 | 7,62 | 7,78 | 8,91 | 8,32 | 8,60 | 8,15 | 7,11 | 7,50 | 7,28 | 7,02 | 6,79 | -0,93 | 0,035 |
| KIAA0363 | 5,83 | 5,53 | 5,39 | 5,72 | 5,68 | 5,84 | 4,65 | 4,63 | 4,71 | 4,99 | 4,73 | 4,73 | -0,93 | 0,001 |
| TOMM22 | 8,84 | 9,00 | 8,88 | 8,87 | 8,87 | 8,94 | 7,82 | 7,28 | 7,55 | 8,61 | 7,87 | 8,74 | -0,92 | 0,020 |
| CYB5R2 | 7,92 | 8,21 | 7,56 | 7,91 | 7,82 | 7,78 | 6,81 | 6,56 | 6,67 | 7,31 | 7,07 | 7,24 | -0,92 | 0,004 |
| ATAD2 | 7,12 | 7,43 | 7,27 | 6,50 | 6,97 | 6,63 | 5,52 | 5,57 | 6,05 | 6,55 | 5,93 | 6,78 | -0,92 | 0,027 |
| GREM1 | 12,57 | 12,19 | 12,29 | 12,12 | 11,55 | 12,12 | 12,19 | 11,18 | 11,19 | 11,40 | 10,51 | 10,84 | -0,92 | 0,034 |
| EHD4 | 8,19 | 7,90 | 8,16 | 7,94 | 7,70 | 7,87 | 7,15 | 6,83 | 7,50 | 7,22 | 6,27 | 7,27 | -0,92 | 0,009 |
| CCDC34 | 8,45 | 8,92 | 8,74 | 8,48 | 8,28 | 8,58 | 7,71 | 7,16 | 7,54 | 8,01 | 7,24 | 8,30 | -0,92 | 0,012 |
| NEDD9 | 6,56 | 5,46 | 5,80 | 5,83 | 5,82 | 5,87 | 4,70 | 4,48 | 4,73 | 5,46 | 5,21 | 5,28 | -0,92 | 0,015 |
| CDC25B | 7,89 | 8,48 | 8,48 | 7,48 | 7,71 | 7,83 | 6,83 | 6,61 | 6,56 | 7,36 | 7,25 | 7,77 | -0,91 | 0,029 |
| TMEM204 | 7,47 | 7,15 | 7,05 | 7,50 | 7,17 | 7,28 | 6,44 | 6,59 | 7,00 | 5,86 | 6,29 | 5,95 | -0,91 | 0,009 |
| LIMA1 | 9,47 | 9,28 | 9,34 | 9,43 | 9,17 | 9,46 | 7,76 | 8,06 | 8,12 | 8,89 | 9,08 | 8,76 | -0,91 | 0,016 |
| LOC731049 | 9,60 | 10,51 | 9,99 | 9,29 | 10,17 | 10,05 | 9,11 | 8,92 | 8,81 | 9,00 | 8,69 | 9,66 | -0,90 | 0,017 |
| CTGF | 13,01 | 12,42 | 13,08 | 12,98 | 13,20 | 13,27 | 11,73 | 12,47 | 11,98 | 12,08 | 12,13 | 12,20 | -0,90 | 0,005 |
| SNORA73A | 6,38 | 5,88 | 6,11 | 5,66 | 5,37 | 5,69 | 4,96 | 4,52 | 5,15 | 5,05 | 4,88 | 5,15 | -0,90 | 0,008 |
| COL8A2 | 6,06 | 5,95 | 6,17 | 6,54 | 6,68 | 6,13 | 5,87 | 5,74 | 5,28 | 5,19 | 4,92 | 5,15 | -0,90 | 0,010 |
| LOC730534 | 12,05 | 12,17 | 12,23 | 11,59 | 11,53 | 11,77 | 11,02 | 10,54 | 10,54 | 11,27 | 11,05 | 11,55 | -0,89 | 0,013 |
| LOC201725 | 6,57 | 6,71 | 6,61 | 6,12 | 6,59 | 6,54 | 5,82 | 5,63 | 5,78 | 5,73 | 5,01 | 5,82 | -0,89 | 0,005 |
| C14orf80 | 5,94 | 6,32 | 6,43 | 5,75 | 5,97 | 6,13 | 5,14 | 4,83 | 5,03 | 5,07 | 4,99 | 6,11 | -0,89 | 0,015 |
| C19orf48 | 6,87 | 7,31 | 6,92 | 6,75 | 6,90 | 6,74 | 6,14 | 5,75 | 6,08 | 6,27 | 5,67 | 6,23 | -0,89 | 0,003 |
| SNORD96A | 6,57 | 6,24 | 6,22 | 5,60 | 6,09 | 6,29 | 5,43 | 5,07 | 5,25 | 5,30 | 4,90 | 5,70 | -0,89 | 0,008 |
| ARAF | 8,76 | 8,83 | 8,80 | 8,93 | 9,21 | 8,97 | 8,10 | 8,46 | 8,26 | 7,99 | 7,41 | 7,93 | -0,89 | 0,006 |
| TRMT5 | 11,29 | 11,58 | 11,44 | 10,66 | 10,61 | 11,22 | 10,36 | 9,51 | 9,66 | 10,63 | 10,44 | 10,84 | -0,89 | 0,041 |
| RILPL2 | 6,37 | 6,47 | 6,20 | 6,25 | 5,58 | 6,04 | 5,29 | 4,50 | 4,54 | 5,89 | 5,74 | 5,61 | -0,89 | 0,044 |
| SNORA61 | 7,61 | 8,16 | 7,65 | 7,46 | 7,56 | 7,49 | 6,54 | 5,97 | 6,74 | 7,16 | 7,00 | 7,19 | -0,89 | 0,016 |
| PAICS | 9,75 | 9,67 | 9,70 | 9,85 | 9,50 | 9,53 | 9,21 | 8,63 | 9,00 | 9,06 | 8,17 | 8,61 | -0,89 | 0,007 |
| LTBP2 | 10,55 | 10,35 | 10,31 | 10,82 | 11,11 | 10,62 | 10,18 | 9,77 | 9,80 | 9,98 | 9,32 | 9,42 | -0,88 | 0,009 |
| NPM3 | 8,12 | 8,27 | 8,31 | 7,91 | 7,58 | 7,97 | 7,18 | 6,24 | 6,85 | 7,81 | 6,96 | 7,81 | -0,88 | 0,040 |
| LOC729505 | 8,65 | 8,92 | 8,87 | 8,16 | 8,24 | 8,51 | 7,72 | 7,22 | 7,46 | 7,91 | 7,48 | 8,26 | -0,88 | 0,012 |
| ZDHHC12 | 6,69 | 7,10 | 6,44 | 6,44 | 6,60 | 7,13 | 5,65 | 5,42 | 5,52 | 5,93 | 6,19 | 6,44 | -0,88 | 0,014 |
| PRIM1 | 6,78 | 7,14 | 6,92 | 6,22 | 6,49 | 6,37 | 5,65 | 5,07 | 5,34 | 6,27 | 5,91 | 6,45 | -0,87 | 0,036 |
| PSMC3IP | 6,46 | 6,51 | 6,67 | 6,03 | 6,66 | 6,18 | 5,53 | 5,27 | 5,80 | 5,54 | 4,87 | 6,29 | -0,87 | 0,020 |
| SGOL2 | 5,94 | 6,13 | 6,23 | 5,52 | 5,85 | 5,93 | 5,29 | 4,96 | 4,89 | 5,36 | 4,81 | 5,09 | -0,87 | 0,004 |
| PPFIA4 | 5,93 | 4,94 | 5,41 | 6,16 | 5,89 | 5,81 | 4,74 | 4,38 | 4,40 | 5,42 | 5,08 | 4,93 | -0,87 | 0,029 |
| GINS3 | 6,65 | 7,11 | 6,53 | 6,53 | 6,91 | 6,32 | 5,69 | 5,16 | 5,98 | 6,11 | 5,65 | 6,27 | -0,87 | 0,014 |
| LOC148915 | 10,98 | 11,41 | 11,19 | 10,39 | 10,54 | 10,91 | 10,00 | 9,58 | 9,51 | 10,38 | 10,11 | 10,64 | -0,86 | 0,027 |
| SMC4 | 7,41 | 7,58 | 7,59 | 6,87 | 6,87 | 6,99 | 6,49 | 5,93 | 6,12 | 6,70 | 6,09 | 6,80 | -0,86 | 0,014 |
| NEIL3 | 5,40 | 5,97 | 6,08 | 5,54 | 5,79 | 5,61 | 5,00 | 4,60 | 4,42 | 5,34 | 4,68 | 5,16 | -0,86 | 0,009 |
| LOC387703 | 8,74 | 9,01 | 8,79 | 8,55 | 7,81 | 8,50 | 7,68 | 7,34 | 7,62 | 7,89 | 7,55 | 8,14 | -0,86 | 0,014 |
| LOC100128007 | 5,65 | 6,37 | 5,51 | 5,41 | 6,07 | 5,80 | 5,08 | 4,69 | 4,79 | 4,96 | 4,94 | 5,18 | -0,86 | 0,007 |
| SKA1 | 6,30 | 6,56 | 6,57 | 5,90 | 6,12 | 5,60 | 4,98 | 5,02 | 5,21 | 5,28 | 5,78 | 5,61 | -0,86 | 0,015 |
| SPAG5 | 5,76 | 6,20 | 6,21 | 5,28 | 5,77 | 5,70 | 4,70 | 4,72 | 5,09 | 5,30 | 4,86 | 5,09 | -0,86 | 0,009 |
| ZFHX4 | 5,93 | 6,40 | 6,33 | 6,14 | 6,28 | 6,35 | 4,62 | 5,29 | 5,44 | 5,73 | 5,69 | 5,55 | -0,85 | 0,010 |
| SNORA76 | 5,84 | 6,05 | 5,81 | 5,65 | 4,95 | 5,83 | 4,83 | 4,42 | 5,23 | 4,61 | 4,60 | 5,33 | -0,85 | 0,020 |
| VTA1 | 6,20 | 5,46 | 6,06 | 6,13 | 6,43 | 6,24 | 5,63 | 5,59 | 5,92 | 5,00 | 4,59 | 4,69 | -0,85 | 0,043 |
| LOC729687 | 8,78 | 8,88 | 8,64 | 8,18 | 8,10 | 8,47 | 7,61 | 7,08 | 7,32 | 7,60 | 7,99 | 8,35 | -0,85 | 0,024 |
| STIL | 7,34 | 7,52 | 7,52 | 6,86 | 7,20 | 7,21 | 6,14 | 6,01 | 6,53 | 6,68 | 6,23 | 6,96 | -0,85 | 0,010 |
| EXOSC9 | 9,13 | 9,34 | 9,13 | 8,56 | 8,82 | 8,86 | 7,99 | 7,50 | 7,76 | 8,69 | 8,08 | 8,73 | -0,85 | 0,026 |
| GADD45B | 8,53 | 8,27 | 8,41 | 8,27 | 8,66 | 8,58 | 7,66 | 7,66 | 7,46 | 7,35 | 7,86 | 7,65 | -0,84 | 0,001 |
| LOC100133372 | 10,90 | 11,26 | 11,08 | 10,75 | 10,59 | 10,95 | 10,35 | 9,81 | 10,23 | 10,06 | 9,67 | 10,37 | -0,84 | 0,006 |
| C17orf53 | 7,06 | 7,38 | 7,20 | 6,80 | 7,01 | 6,95 | 6,58 | 6,21 | 6,03 | 6,39 | 5,52 | 6,62 | -0,84 | 0,012 |
| TIMELESS | 7,95 | 8,40 | 8,30 | 7,51 | 7,69 | 7,96 | 7,11 | 6,44 | 6,95 | 7,53 | 6,98 | 7,75 | -0,84 | 0,029 |
| GPI | 9,26 | 9,17 | 9,33 | 9,81 | 10,14 | 9,62 | 8,69 | 9,19 | 8,72 | 8,52 | 8,61 | 8,57 | -0,84 | 0,012 |
| UAP1 | 10,88 | 10,82 | 11,02 | 11,02 | 11,45 | 10,78 | 9,61 | 9,69 | 9,99 | 10,37 | 10,59 | 10,69 | -0,84 | 0,020 |
| GJA1 | 9,80 | 10,11 | 9,97 | 10,52 | 10,34 | 10,22 | 9,03 | 9,50 | 9,57 | 9,18 | 9,29 | 9,36 | -0,84 | 0,004 |
| LOC100132528 | 11,02 | 11,29 | 11,05 | 10,75 | 10,57 | 10,93 | 10,38 | 9,92 | 10,10 | 10,16 | 9,60 | 10,43 | -0,84 | 0,008 |
| BCL2L12 | 7,92 | 8,34 | 8,20 | 7,62 | 7,95 | 7,88 | 6,88 | 6,79 | 6,77 | 7,61 | 6,88 | 7,99 | -0,83 | 0,030 |
| MTR | 8,18 | 7,95 | 8,17 | 8,27 | 7,96 | 7,88 | 7,74 | 7,76 | 7,62 | 7,06 | 6,35 | 6,87 | -0,83 | 0,033 |
| SLC38A1 | 6,77 | 6,34 | 6,82 | 6,66 | 6,92 | 6,66 | 6,25 | 6,19 | 6,02 | 5,68 | 5,65 | 5,38 | -0,83 | 0,008 |
| TTF2 | 6,82 | 7,36 | 7,10 | 6,73 | 7,24 | 6,72 | 5,74 | 5,77 | 6,03 | 6,69 | 6,16 | 6,57 | -0,83 | 0,016 |
| ZWILCH | 6,97 | 6,89 | 7,01 | 6,17 | 6,30 | 6,65 | 5,66 | 6,09 | 5,79 | 5,93 | 5,43 | 6,11 | -0,83 | 0,011 |
| CDC25C | 5,54 | 6,18 | 5,81 | 5,23 | 5,33 | 5,46 | 4,61 | 4,45 | 4,43 | 4,81 | 4,78 | 5,48 | -0,83 | 0,020 |
| MCM7 | 8,22 | 8,56 | 8,16 | 7,55 | 7,78 | 7,59 | 7,19 | 6,54 | 6,83 | 7,44 | 7,12 | 7,79 | -0,82 | 0,036 |
| MCM10 | 5,71 | 6,29 | 5,77 | 5,55 | 5,85 | 5,40 | 5,12 | 4,48 | 5,02 | 5,06 | 4,58 | 5,39 | -0,82 | 0,014 |
| KCTD11 | 5,82 | 5,54 | 5,50 | 6,70 | 5,69 | 6,00 | 5,30 | 5,34 | 5,03 | 4,94 | 4,95 | 4,74 | -0,82 | 0,019 |
| IMPDH2 | 9,86 | 9,91 | 9,94 | 9,80 | 9,37 | 9,89 | 9,02 | 8,60 | 9,06 | 9,08 | 8,97 | 9,13 | -0,82 | 0,003 |
| ZDHHC4 | 7,55 | 7,82 | 7,77 | 7,96 | 7,70 | 7,85 | 6,76 | 6,85 | 6,54 | 7,28 | 7,29 | 7,01 | -0,82 | 0,005 |
| APOBEC3B | 5,44 | 6,19 | 5,47 | 4,80 | 5,08 | 4,84 | 4,28 | 4,52 | 4,33 | 4,57 | 4,61 | 4,64 | -0,82 | 0,026 |
| PFN2 | 9,69 | 10,14 | 9,96 | 9,76 | 9,91 | 9,98 | 9,10 | 9,22 | 9,14 | 9,25 | 8,59 | 9,27 | -0,81 | 0,004 |
| FN3KRP | 9,87 | 9,91 | 9,89 | 9,77 | 9,76 | 9,83 | 9,00 | 8,43 | 8,81 | 9,54 | 8,67 | 9,71 | -0,81 | 0,020 |
| DLC1 | 7,62 | 7,05 | 7,23 | 7,20 | 6,68 | 7,41 | 6,55 | 6,53 | 6,70 | 6,49 | 5,69 | 6,36 | -0,81 | 0,017 |
| CENPH | 6,43 | 6,41 | 6,32 | 5,72 | 5,68 | 5,93 | 5,15 | 5,22 | 5,13 | 5,21 | 5,12 | 5,80 | -0,81 | 0,012 |
| SAP30 | 8,99 | 8,91 | 8,95 | 8,82 | 8,61 | 8,63 | 7,74 | 7,30 | 7,70 | 8,34 | 8,15 | 8,79 | -0,81 | 0,029 |
| C4orf46 | 6,18 | 6,64 | 6,65 | 6,00 | 6,32 | 6,49 | 5,66 | 5,31 | 5,83 | 5,63 | 5,16 | 5,84 | -0,81 | 0,008 |
| XRCC6 | 9,61 | 9,75 | 9,60 | 9,31 | 9,31 | 9,51 | 8,61 | 8,37 | 8,48 | 9,06 | 8,68 | 9,06 | -0,81 | 0,006 |
| MCM5 | 7,39 | 7,80 | 7,65 | 6,80 | 7,08 | 7,14 | 6,40 | 6,31 | 6,29 | 6,91 | 6,18 | 6,96 | -0,80 | 0,021 |
| LOC286016 | 11,10 | 10,76 | 10,63 | 11,60 | 11,15 | 11,07 | 10,51 | 10,36 | 10,19 | 10,33 | 9,80 | 10,32 | -0,80 | 0,011 |
| C1orf112 | 6,22 | 6,48 | 6,12 | 5,72 | 6,08 | 5,66 | 5,21 | 5,09 | 5,20 | 5,54 | 4,98 | 5,47 | -0,80 | 0,008 |
| KCTD15 | 7,14 | 6,91 | 7,42 | 7,25 | 6,45 | 7,11 | 6,75 | 6,39 | 6,34 | 6,02 | 6,05 | 5,97 | -0,80 | 0,014 |
| GALNT12 | 5,67 | 6,85 | 5,94 | 5,79 | 6,05 | 5,82 | 5,09 | 4,83 | 5,46 | 5,14 | 5,67 | 5,17 | -0,79 | 0,024 |
| E2F2 | 5,88 | 5,83 | 5,75 | 5,53 | 5,38 | 5,47 | 4,43 | 4,61 | 4,75 | 5,14 | 4,75 | 5,40 | -0,79 | 0,011 |
| CDK2 | 6,83 | 7,39 | 7,06 | 6,62 | 7,05 | 6,92 | 5,77 | 5,93 | 6,10 | 6,50 | 6,08 | 6,74 | -0,79 | 0,015 |
| SUV39H1 | 7,71 | 8,26 | 7,90 | 7,42 | 8,04 | 7,45 | 6,95 | 7,22 | 6,90 | 7,19 | 6,42 | 7,40 | -0,79 | 0,019 |
| MKI67 | 4,87 | 5,53 | 5,65 | 4,88 | 5,40 | 4,57 | 4,42 | 4,26 | 4,31 | 4,40 | 4,37 | 4,43 | -0,78 | 0,014 |
| DHFR | 6,17 | 6,34 | 6,58 | 5,69 | 5,71 | 6,00 | 4,92 | 5,39 | 5,29 | 5,46 | 5,04 | 5,69 | -0,78 | 0,015 |
| RNU105A | 6,21 | 6,07 | 6,03 | 5,43 | 4,95 | 5,81 | 5,00 | 4,32 | 5,17 | 5,24 | 4,77 | 5,31 | -0,78 | 0,048 |
| LYL1 | 6,17 | 6,29 | 6,29 | 5,89 | 5,98 | 6,41 | 5,61 | 4,98 | 5,01 | 5,42 | 5,39 | 5,99 | -0,77 | 0,014 |
| AKAP7 | 5,97 | 6,26 | 6,17 | 5,90 | 5,57 | 5,67 | 5,41 | 4,85 | 4,65 | 5,40 | 5,45 | 5,20 | -0,76 | 0,015 |
| HIF1A | 9,39 | 9,30 | 9,24 | 8,94 | 8,78 | 9,22 | 8,54 | 8,87 | 8,43 | 8,24 | 7,89 | 8,33 | -0,76 | 0,012 |
| MCM3 | 8,47 | 8,95 | 8,57 | 8,03 | 8,42 | 8,06 | 7,35 | 7,06 | 7,64 | 8,15 | 7,48 | 8,25 | -0,76 | 0,045 |
| GBAS | 7,55 | 7,66 | 7,17 | 7,71 | 7,74 | 7,25 | 7,13 | 7,16 | 6,69 | 6,67 | 6,41 | 6,48 | -0,76 | 0,012 |
| SMC2 | 6,23 | 6,73 | 6,37 | 5,96 | 6,40 | 5,96 | 5,61 | 5,47 | 5,74 | 5,55 | 5,02 | 5,74 | -0,75 | 0,012 |
| CCDC109B | 9,73 | 10,06 | 9,76 | 9,89 | 10,15 | 9,91 | 8,53 | 8,59 | 8,98 | 9,48 | 9,62 | 9,77 | -0,75 | 0,043 |
| SGOL1 | 5,86 | 6,08 | 5,88 | 5,34 | 5,80 | 5,44 | 5,07 | 4,84 | 4,81 | 4,99 | 4,76 | 5,39 | -0,75 | 0,009 |
| CARM1 | 10,29 | 10,24 | 10,38 | 10,22 | 10,29 | 10,44 | 9,88 | 9,77 | 10,01 | 9,41 | 8,70 | 9,56 | -0,75 | 0,023 |
| LOC653874 | 8,63 | 8,92 | 8,60 | 8,17 | 8,11 | 8,21 | 7,44 | 7,25 | 7,48 | 8,08 | 7,70 | 8,18 | -0,75 | 0,026 |
| GRSF1 | 9,48 | 9,27 | 9,52 | 9,47 | 9,55 | 9,58 | 9,12 | 8,99 | 9,34 | 8,68 | 7,77 | 8,45 | -0,75 | 0,047 |
| GPSM2 | 6,88 | 7,22 | 7,06 | 6,27 | 6,79 | 6,60 | 5,77 | 5,65 | 5,71 | 6,17 | 6,10 | 6,92 | -0,75 | 0,049 |
| LOC100131609 | 10,72 | 10,98 | 10,86 | 10,42 | 10,40 | 10,64 | 10,03 | 9,59 | 9,93 | 10,04 | 9,63 | 10,31 | -0,75 | 0,009 |
| LOC729816 | 9,98 | 10,37 | 10,02 | 9,74 | 9,59 | 9,80 | 8,84 | 8,75 | 8,66 | 9,65 | 9,32 | 9,80 | -0,75 | 0,044 |
| IL11RA | 7,18 | 7,22 | 7,50 | 7,21 | 7,19 | 7,23 | 6,44 | 6,12 | 6,01 | 6,99 | 6,49 | 7,03 | -0,74 | 0,019 |
| WWP2 | 6,80 | 6,91 | 6,69 | 6,89 | 7,07 | 6,64 | 6,34 | 6,32 | 5,96 | 5,94 | 5,83 | 6,14 | -0,74 | 0,004 |
| LOC730246 | 11,80 | 12,12 | 11,99 | 11,58 | 11,63 | 11,85 | 11,20 | 11,00 | 11,22 | 11,07 | 10,56 | 11,47 | -0,74 | 0,010 |
| LOC644037 | 8,31 | 8,40 | 8,33 | 8,12 | 7,64 | 7,96 | 7,55 | 7,35 | 7,50 | 7,40 | 6,81 | 7,70 | -0,74 | 0,015 |
| LOC402112 | 8,89 | 9,15 | 8,70 | 8,82 | 8,59 | 8,98 | 8,48 | 7,93 | 8,20 | 8,21 | 7,48 | 8,37 | -0,74 | 0,014 |
| NGF | 8,67 | 8,43 | 8,37 | 8,58 | 8,38 | 8,49 | 7,45 | 7,85 | 7,74 | 7,77 | 7,77 | 7,90 | -0,74 | 0,001 |
| KPNA2 | 9,24 | 9,70 | 9,56 | 8,88 | 9,65 | 9,33 | 8,74 | 8,79 | 8,93 | 8,49 | 7,91 | 9,06 | -0,74 | 0,033 |
| EEF1E1 | 8,86 | 9,04 | 8,83 | 8,50 | 8,84 | 8,73 | 7,94 | 8,04 | 8,52 | 8,03 | 7,45 | 8,39 | -0,74 | 0,015 |
| SH3PXD2A | 10,93 | 10,98 | 11,15 | 11,15 | 10,79 | 11,44 | 11,12 | 10,37 | 10,66 | 10,15 | 9,88 | 9,84 | -0,74 | 0,041 |
| MXD3 | 5,54 | 6,21 | 5,44 | 5,28 | 5,75 | 5,29 | 4,85 | 4,38 | 4,71 | 5,22 | 4,79 | 5,14 | -0,74 | 0,023 |
| LOC643319 | 7,77 | 7,42 | 7,61 | 7,84 | 7,81 | 7,88 | 7,32 | 7,14 | 7,28 | 6,63 | 6,69 | 6,85 | -0,74 | 0,009 |
| KNTC1 | 6,65 | 7,27 | 6,91 | 6,53 | 6,55 | 6,48 | 6,42 | 5,43 | 5,81 | 6,36 | 5,62 | 6,33 | -0,73 | 0,037 |
| TNS1 | 7,09 | 7,23 | 7,22 | 6,96 | 7,02 | 7,13 | 6,64 | 7,06 | 6,64 | 6,17 | 5,60 | 6,13 | -0,73 | 0,036 |
| INTS9 | 7,49 | 7,45 | 7,53 | 7,58 | 7,26 | 7,41 | 6,46 | 6,20 | 6,49 | 7,19 | 6,87 | 7,11 | -0,73 | 0,015 |
| SHMT1 | 5,54 | 6,16 | 5,88 | 5,55 | 5,74 | 5,45 | 4,77 | 4,51 | 4,67 | 5,38 | 5,12 | 5,49 | -0,73 | 0,027 |
| ZNF275 | 7,75 | 7,55 | 7,84 | 7,64 | 7,71 | 7,71 | 7,23 | 7,24 | 7,22 | 6,91 | 6,68 | 6,53 | -0,73 | 0,008 |
| LOC100129267 | 6,04 | 6,14 | 5,81 | 5,63 | 5,51 | 5,68 | 4,95 | 5,06 | 5,32 | 5,44 | 5,01 | 4,64 | -0,73 | 0,012 |
| C6orf145 | 7,11 | 5,97 | 6,67 | 6,77 | 6,63 | 7,29 | 6,29 | 5,92 | 5,72 | 6,24 | 6,01 | 5,88 | -0,73 | 0,033 |
| LOC100128836 | 8,44 | 8,51 | 8,75 | 8,38 | 8,10 | 8,31 | 7,87 | 7,46 | 7,81 | 7,58 | 7,15 | 8,25 | -0,73 | 0,019 |
| DARS2 | 6,67 | 6,95 | 6,88 | 6,50 | 6,59 | 6,54 | 5,95 | 5,80 | 6,16 | 6,02 | 5,57 | 6,26 | -0,73 | 0,007 |
| C11orf41 | 7,64 | 7,72 | 7,80 | 7,36 | 7,39 | 7,58 | 7,31 | 6,69 | 7,60 | 6,76 | 6,16 | 6,60 | -0,73 | 0,046 |
| TH1L | 8,01 | 8,06 | 8,21 | 8,12 | 7,90 | 8,03 | 7,86 | 7,61 | 7,84 | 7,21 | 6,47 | 7,02 | -0,72 | 0,048 |
| POLR1E | 7,58 | 7,94 | 7,74 | 7,72 | 7,48 | 7,45 | 6,82 | 6,27 | 6,99 | 7,03 | 7,07 | 7,39 | -0,72 | 0,017 |
| MDC1 | 8,07 | 8,21 | 8,24 | 7,73 | 7,81 | 8,02 | 7,13 | 6,62 | 6,96 | 7,74 | 7,33 | 8,01 | -0,72 | 0,049 |
| C1orf135 | 5,90 | 5,88 | 5,33 | 4,66 | 5,43 | 5,02 | 4,62 | 4,59 | 4,48 | 4,95 | 4,34 | 4,93 | -0,72 | 0,046 |
| MTP18 | 6,56 | 6,63 | 6,04 | 6,67 | 6,88 | 6,46 | 5,95 | 5,96 | 5,73 | 5,89 | 5,71 | 5,70 | -0,72 | 0,007 |
| SERBP1 | 6,76 | 7,20 | 7,45 | 7,10 | 7,26 | 7,00 | 6,39 | 5,78 | 6,26 | 6,92 | 6,33 | 6,78 | -0,72 | 0,026 |
| B3GALNT1 | 5,24 | 5,56 | 5,40 | 5,23 | 5,48 | 5,67 | 4,49 | 4,56 | 4,34 | 5,17 | 4,99 | 4,73 | -0,72 | 0,011 |
| HSPD1 | 10,44 | 10,60 | 10,55 | 10,15 | 10,90 | 10,27 | 9,91 | 10,24 | 10,18 | 9,63 | 9,07 | 9,59 | -0,72 | 0,036 |
| RPL22L1 | 5,90 | 5,38 | 5,64 | 5,89 | 5,36 | 5,72 | 5,44 | 4,75 | 5,14 | 4,69 | 4,66 | 4,92 | -0,72 | 0,013 |
| CIT | 5,66 | 5,38 | 5,81 | 5,30 | 4,85 | 5,66 | 4,80 | 4,26 | 5,15 | 4,66 | 4,50 | 5,00 | -0,71 | 0,029 |
| C16orf33 | 8,51 | 8,84 | 8,65 | 8,25 | 8,39 | 8,66 | 7,97 | 8,10 | 7,72 | 7,58 | 7,57 | 8,07 | -0,71 | 0,008 |
| TCF19 | 5,65 | 5,84 | 5,47 | 5,01 | 5,39 | 5,26 | 4,61 | 4,50 | 4,58 | 4,94 | 4,62 | 5,09 | -0,71 | 0,012 |
| RABGGTB | 8,85 | 8,78 | 8,86 | 8,76 | 8,70 | 8,88 | 7,65 | 7,73 | 8,03 | 8,47 | 8,35 | 8,32 | -0,71 | 0,010 |
| TGOLN2 | 10,59 | 10,39 | 10,62 | 10,67 | 10,66 | 10,55 | 9,54 | 9,29 | 9,55 | 10,44 | 10,13 | 10,28 | -0,71 | 0,031 |
| BRCA1 | 5,30 | 5,77 | 5,57 | 5,28 | 5,84 | 5,48 | 4,55 | 4,56 | 4,80 | 5,04 | 5,02 | 5,03 | -0,71 | 0,009 |
| ATP8B2 | 6,57 | 6,44 | 6,58 | 6,53 | 6,27 | 6,41 | 5,51 | 5,15 | 5,42 | 6,28 | 5,95 | 6,26 | -0,71 | 0,032 |
| SF3B3 | 7,16 | 7,66 | 7,38 | 7,36 | 7,64 | 7,28 | 7,02 | 7,17 | 6,67 | 6,78 | 5,91 | 6,70 | -0,71 | 0,031 |
| CKS1B | 9,30 | 9,86 | 9,47 | 8,97 | 9,54 | 9,64 | 8,84 | 8,62 | 8,65 | 8,68 | 8,29 | 9,46 | -0,70 | 0,035 |
| DR1 | 6,84 | 7,33 | 7,45 | 7,19 | 7,46 | 7,30 | 6,12 | 6,22 | 6,63 | 6,94 | 6,63 | 6,80 | -0,70 | 0,016 |
| LOC732165 | 9,84 | 9,60 | 9,47 | 10,47 | 9,94 | 9,85 | 9,35 | 9,20 | 9,04 | 9,00 | 9,22 | 9,14 | -0,70 | 0,012 |
| E2F4 | 8,05 | 8,32 | 8,14 | 8,23 | 8,13 | 8,17 | 7,43 | 7,35 | 7,47 | 7,71 | 7,14 | 7,71 | -0,70 | 0,004 |
| FAM171A1 | 8,88 | 9,27 | 9,19 | 8,86 | 9,59 | 9,32 | 8,16 | 8,44 | 8,84 | 8,48 | 8,32 | 8,65 | -0,70 | 0,012 |
| RRP1B | 5,68 | 5,27 | 5,47 | 5,11 | 5,28 | 5,42 | 4,56 | 4,37 | 4,86 | 4,91 | 4,61 | 4,71 | -0,70 | 0,006 |
| ACSL5 | 6,29 | 6,58 | 5,89 | 6,17 | 5,91 | 6,05 | 5,64 | 5,48 | 5,24 | 5,68 | 5,32 | 5,32 | -0,70 | 0,008 |
| DHFRL1 | 6,83 | 7,16 | 7,22 | 6,62 | 6,61 | 6,84 | 6,26 | 5,98 | 6,27 | 6,32 | 5,62 | 6,63 | -0,70 | 0,021 |
| SNORD16 | 6,26 | 5,83 | 6,02 | 5,85 | 5,79 | 5,94 | 5,32 | 5,48 | 5,52 | 5,46 | 4,97 | 4,77 | -0,70 | 0,012 |
| FAM158A | 7,09 | 6,99 | 7,11 | 7,16 | 6,87 | 7,28 | 5,97 | 6,22 | 6,22 | 6,46 | 6,59 | 6,89 | -0,69 | 0,012 |
| LOC100129673 | 7,25 | 7,64 | 7,02 | 7,37 | 7,59 | 7,36 | 6,60 | 6,05 | 6,42 | 6,98 | 6,70 | 7,34 | -0,69 | 0,041 |
| LOC728732 | 7,98 | 8,57 | 8,29 | 7,89 | 7,62 | 8,17 | 7,68 | 7,36 | 7,32 | 7,48 | 6,99 | 7,58 | -0,69 | 0,019 |
| LOC643995 | 5,77 | 5,95 | 5,93 | 5,49 | 5,33 | 5,46 | 5,07 | 4,71 | 4,99 | 4,99 | 4,74 | 5,30 | -0,69 | 0,011 |
| ERBB2 | 7,94 | 7,98 | 8,04 | 8,03 | 8,25 | 8,21 | 7,32 | 7,19 | 7,09 | 7,57 | 7,65 | 7,50 | -0,69 | 0,005 |
| LOC645691 | 8,37 | 8,43 | 8,37 | 8,08 | 7,83 | 8,17 | 7,58 | 7,31 | 7,61 | 7,49 | 7,25 | 7,89 | -0,69 | 0,009 |
| SNORA70 | 7,66 | 7,66 | 7,50 | 7,33 | 6,95 | 7,60 | 6,89 | 6,98 | 6,94 | 6,84 | 6,26 | 6,65 | -0,69 | 0,016 |
| LOC645436 | 11,40 | 11,45 | 11,13 | 10,65 | 10,84 | 11,16 | 10,49 | 10,13 | 10,41 | 10,52 | 10,10 | 10,87 | -0,69 | 0,022 |
| SHPK | 8,13 | 8,49 | 8,24 | 7,96 | 8,10 | 8,13 | 7,58 | 6,80 | 7,78 | 7,79 | 7,32 | 7,67 | -0,68 | 0,021 |
| TAGLN2 | 9,06 | 8,99 | 9,11 | 9,16 | 9,19 | 9,22 | 8,53 | 8,63 | 8,79 | 8,25 | 7,97 | 8,46 | -0,68 | 0,008 |
| HOMER3 | 7,09 | 6,84 | 6,54 | 7,31 | 7,02 | 7,07 | 6,27 | 5,76 | 6,63 | 6,25 | 6,11 | 6,73 | -0,68 | 0,026 |
| TXNDC5 | 8,24 | 8,40 | 8,33 | 8,36 | 8,66 | 8,31 | 7,45 | 7,50 | 7,39 | 8,06 | 8,19 | 7,61 | -0,68 | 0,014 |
| LOC642817 | 9,94 | 10,16 | 10,08 | 9,77 | 9,48 | 9,61 | 9,13 | 9,27 | 9,11 | 9,22 | 9,11 | 9,10 | -0,68 | 0,006 |
| AACS | 5,75 | 6,03 | 5,81 | 5,99 | 6,50 | 5,94 | 5,31 | 5,19 | 5,41 | 5,72 | 5,48 | 4,81 | -0,68 | 0,019 |
| KIAA1712 | 7,13 | 7,69 | 6,89 | 7,22 | 7,50 | 7,04 | 6,20 | 6,24 | 6,31 | 7,12 | 6,90 | 6,64 | -0,68 | 0,037 |
| C22orf27 | 5,63 | 4,92 | 5,32 | 5,98 | 5,10 | 5,86 | 4,49 | 4,77 | 4,85 | 4,82 | 5,03 | 4,75 | -0,68 | 0,031 |
| UBE4A | 8,37 | 8,14 | 8,14 | 8,39 | 8,30 | 8,19 | 7,43 | 7,53 | 7,54 | 7,91 | 7,41 | 7,60 | -0,68 | 0,003 |
| LOC728643 | 10,48 | 10,81 | 10,33 | 10,01 | 9,69 | 10,15 | 9,63 | 9,35 | 9,81 | 9,63 | 9,21 | 9,77 | -0,68 | 0,030 |
| LOC100130790 | 5,22 | 4,96 | 4,68 | 5,59 | 5,68 | 4,96 | 4,55 | 4,63 | 4,38 | 4,56 | 4,36 | 4,52 | -0,68 | 0,019 |
| FNBP1L | 7,69 | 7,41 | 7,31 | 7,97 | 7,84 | 7,67 | 7,05 | 6,86 | 6,80 | 7,07 | 7,06 | 6,97 | -0,68 | 0,006 |
| NFIA | 5,53 | 5,80 | 5,68 | 5,11 | 5,75 | 5,72 | 4,75 | 5,27 | 4,71 | 4,81 | 5,07 | 4,92 | -0,68 | 0,011 |
| JMJD8 | 10,19 | 10,34 | 10,28 | 10,24 | 10,36 | 10,20 | 9,66 | 9,79 | 9,56 | 9,33 | 9,48 | 9,74 | -0,67 | 0,002 |
| PACSIN2 | 7,62 | 7,71 | 7,73 | 7,45 | 7,54 | 7,78 | 6,78 | 7,12 | 7,33 | 6,97 | 6,75 | 6,86 | -0,67 | 0,005 |
| CNN2 | 8,93 | 8,68 | 8,83 | 8,88 | 8,43 | 8,81 | 8,02 | 8,27 | 7,73 | 8,13 | 7,99 | 8,40 | -0,67 | 0,008 |
| NFIX | 10,12 | 10,07 | 10,40 | 10,13 | 9,96 | 10,04 | 9,39 | 9,40 | 9,41 | 9,49 | 9,38 | 9,64 | -0,67 | 0,002 |
| PODNL1 | 5,62 | 5,58 | 5,15 | 5,48 | 5,22 | 5,69 | 4,94 | 4,86 | 4,88 | 4,57 | 4,91 | 4,57 | -0,67 | 0,007 |
| FAM122B | 6,19 | 6,63 | 6,49 | 6,08 | 6,47 | 6,37 | 5,19 | 5,27 | 5,93 | 6,09 | 5,86 | 5,90 | -0,67 | 0,027 |
| LOC400455 | 9,14 | 9,17 | 9,23 | 9,27 | 9,04 | 9,24 | 8,95 | 8,63 | 8,75 | 8,53 | 7,98 | 8,27 | -0,67 | 0,014 |
| TARBP1 | 5,42 | 5,66 | 5,78 | 5,50 | 5,44 | 5,57 | 5,02 | 4,46 | 4,83 | 5,05 | 5,22 | 4,78 | -0,67 | 0,008 |
| GAS1 | 9,30 | 9,22 | 9,25 | 9,86 | 8,73 | 9,47 | 9,05 | 8,59 | 8,58 | 8,53 | 8,63 | 8,47 | -0,67 | 0,025 |
| GGT3 | 7,27 | 7,43 | 7,07 | 6,90 | 7,15 | 7,17 | 6,76 | 6,74 | 6,56 | 6,31 | 6,33 | 6,29 | -0,67 | 0,007 |
| RNPEP | 8,73 | 8,98 | 8,98 | 8,78 | 9,05 | 8,88 | 8,05 | 8,20 | 7,89 | 8,52 | 8,23 | 8,52 | -0,66 | 0,007 |
| NR2C2AP | 6,67 | 6,78 | 6,60 | 5,84 | 6,35 | 6,14 | 5,71 | 5,33 | 5,83 | 6,21 | 5,33 | 5,97 | -0,66 | 0,048 |
| PDXK | 8,61 | 8,86 | 8,81 | 9,00 | 8,56 | 8,86 | 8,37 | 8,25 | 8,21 | 7,88 | 7,76 | 8,25 | -0,66 | 0,008 |
| NXT2 | 7,06 | 7,11 | 7,05 | 7,00 | 7,11 | 6,74 | 6,53 | 6,85 | 6,71 | 6,38 | 5,54 | 6,08 | -0,66 | 0,048 |
| MAP6D1 | 5,86 | 6,02 | 6,05 | 6,37 | 6,85 | 6,76 | 5,83 | 5,93 | 5,72 | 5,36 | 5,43 | 5,68 | -0,66 | 0,037 |
| C16orf75 | 7,24 | 7,22 | 7,09 | 7,05 | 6,83 | 6,89 | 6,45 | 6,42 | 6,11 | 6,55 | 5,93 | 6,88 | -0,66 | 0,017 |
| KIAA1522 | 5,52 | 5,21 | 5,59 | 5,19 | 5,85 | 5,32 | 5,12 | 4,97 | 4,92 | 4,62 | 4,60 | 4,50 | -0,66 | 0,015 |
| AAGAB | 7,06 | 7,10 | 7,06 | 6,60 | 6,66 | 6,85 | 6,36 | 6,46 | 6,14 | 6,21 | 5,88 | 6,32 | -0,66 | 0,009 |
| LOC729102 | 11,28 | 11,68 | 11,36 | 10,83 | 11,03 | 11,22 | 10,71 | 10,40 | 10,51 | 10,69 | 10,17 | 10,96 | -0,66 | 0,022 |
| HYLS1 | 7,18 | 7,63 | 7,50 | 6,60 | 7,23 | 7,12 | 6,33 | 6,29 | 6,44 | 6,69 | 6,47 | 7,09 | -0,66 | 0,038 |
| UGDH | 10,37 | 10,29 | 10,56 | 10,09 | 9,91 | 10,38 | 9,61 | 9,34 | 9,71 | 9,80 | 9,07 | 10,13 | -0,66 | 0,030 |
| LOC653506 | 11,31 | 11,18 | 11,43 | 11,39 | 11,20 | 11,28 | 10,85 | 10,93 | 10,97 | 10,61 | 10,14 | 10,37 | -0,66 | 0,014 |
| H2AFZ | 12,45 | 12,73 | 12,84 | 12,15 | 12,32 | 12,50 | 11,72 | 11,56 | 11,74 | 12,08 | 11,60 | 12,36 | -0,66 | 0,023 |
| EMG1 | 8,54 | 8,91 | 8,49 | 8,30 | 8,39 | 8,52 | 8,00 | 8,14 | 8,14 | 7,62 | 7,49 | 7,83 | -0,65 | 0,014 |
| HAUS6 | 5,47 | 5,70 | 5,40 | 5,70 | 5,14 | 5,36 | 4,48 | 4,53 | 4,79 | 4,96 | 4,98 | 5,10 | -0,65 | 0,012 |
| C15orf21 | 5,52 | 5,66 | 5,64 | 5,23 | 4,98 | 5,22 | 4,64 | 4,47 | 4,49 | 4,74 | 5,08 | 4,93 | -0,65 | 0,017 |
| LOC399942 | 10,83 | 11,05 | 10,98 | 10,20 | 10,79 | 10,87 | 10,04 | 10,44 | 10,50 | 9,84 | 9,63 | 10,37 | -0,65 | 0,039 |
| PCNA | 8,26 | 8,50 | 8,11 | 7,75 | 7,86 | 7,95 | 7,81 | 7,49 | 7,41 | 7,14 | 6,99 | 7,68 | -0,65 | 0,026 |
| UBE2J1 | 7,46 | 7,65 | 7,44 | 7,42 | 7,65 | 7,39 | 6,83 | 7,06 | 6,81 | 6,66 | 6,87 | 6,88 | -0,65 | 0,002 |
| LOC729423 | 7,85 | 8,10 | 7,83 | 7,58 | 7,47 | 7,92 | 7,30 | 7,06 | 7,32 | 7,02 | 6,57 | 7,56 | -0,65 | 0,025 |
| NSF | 6,81 | 6,68 | 6,87 | 6,46 | 6,77 | 7,00 | 6,49 | 6,55 | 6,35 | 5,69 | 5,83 | 5,77 | -0,65 | 0,031 |
| C3orf26 | 7,84 | 8,18 | 7,89 | 7,83 | 8,10 | 8,25 | 7,77 | 7,34 | 7,66 | 7,12 | 6,80 | 7,53 | -0,65 | 0,025 |
| TP53RK | 6,18 | 6,26 | 6,45 | 6,33 | 6,16 | 6,31 | 5,32 | 6,01 | 5,90 | 5,49 | 5,53 | 5,57 | -0,65 | 0,008 |
| CDKN2A | 5,56 | 5,79 | 5,61 | 5,28 | 5,10 | 5,56 | 5,04 | 4,59 | 4,83 | 4,89 | 4,78 | 4,89 | -0,65 | 0,009 |
| TOR1AIP1 | 8,39 | 8,17 | 8,20 | 8,32 | 8,03 | 8,09 | 7,88 | 7,88 | 8,00 | 7,42 | 6,92 | 7,21 | -0,65 | 0,038 |
| PLP2 | 7,39 | 7,57 | 7,32 | 7,43 | 7,49 | 7,55 | 6,46 | 6,74 | 7,06 | 6,80 | 6,41 | 7,40 | -0,65 | 0,020 |
| MAGED1 | 10,34 | 10,26 | 10,54 | 10,32 | 10,08 | 10,41 | 9,76 | 9,21 | 9,61 | 10,00 | 9,75 | 9,74 | -0,65 | 0,010 |
| HNRNPA1 | 6,37 | 6,77 | 6,64 | 6,24 | 6,20 | 6,29 | 6,07 | 5,49 | 6,21 | 5,70 | 5,24 | 5,92 | -0,65 | 0,032 |
| LOC647346 | 9,11 | 9,14 | 9,17 | 9,12 | 8,99 | 9,19 | 8,96 | 8,76 | 8,90 | 8,25 | 7,85 | 8,13 | -0,65 | 0,041 |
| SEC23B | 8,58 | 8,28 | 8,34 | 8,35 | 8,32 | 8,24 | 7,79 | 7,63 | 8,03 | 7,77 | 7,37 | 7,65 | -0,64 | 0,006 |
| DAP | 8,62 | 8,35 | 8,37 | 8,52 | 8,85 | 8,55 | 8,00 | 8,00 | 8,13 | 7,61 | 8,00 | 7,67 | -0,64 | 0,008 |
| PRKCA | 8,36 | 8,84 | 8,57 | 8,34 | 8,83 | 8,76 | 7,78 | 8,21 | 8,17 | 8,17 | 7,80 | 7,73 | -0,64 | 0,012 |
| GNG12 | 8,06 | 8,18 | 8,42 | 8,10 | 8,46 | 8,29 | 7,29 | 7,46 | 7,25 | 7,85 | 7,73 | 8,10 | -0,64 | 0,020 |
| BUB3 | 8,64 | 8,62 | 8,59 | 8,31 | 8,72 | 8,47 | 7,64 | 7,65 | 7,98 | 7,97 | 7,82 | 8,46 | -0,64 | 0,014 |
| NUP107 | 7,86 | 8,07 | 8,09 | 7,53 | 7,99 | 7,81 | 7,26 | 7,04 | 7,19 | 7,47 | 7,04 | 7,53 | -0,64 | 0,009 |
| PDK3 | 5,49 | 5,29 | 5,18 | 5,84 | 5,68 | 4,93 | 4,85 | 4,86 | 4,85 | 4,60 | 4,77 | 4,64 | -0,64 | 0,017 |
| H2AFX | 6,73 | 7,06 | 6,91 | 6,53 | 6,48 | 6,79 | 6,16 | 5,88 | 5,66 | 6,37 | 5,84 | 6,74 | -0,64 | 0,041 |
| LOC389049 | 6,02 | 6,53 | 6,21 | 5,78 | 6,03 | 6,11 | 5,64 | 5,91 | 5,78 | 5,17 | 5,01 | 5,33 | -0,64 | 0,034 |
| PYCARD | 6,32 | 6,63 | 6,56 | 6,32 | 6,22 | 6,37 | 6,02 | 5,47 | 5,55 | 6,20 | 5,53 | 5,82 | -0,64 | 0,014 |
| RAD54L | 5,42 | 6,06 | 5,62 | 5,29 | 5,72 | 5,24 | 5,38 | 4,58 | 4,72 | 5,10 | 4,62 | 5,11 | -0,64 | 0,038 |
| SRI | 7,61 | 7,54 | 7,59 | 7,52 | 7,27 | 7,74 | 7,02 | 6,33 | 6,80 | 7,30 | 6,75 | 7,23 | -0,64 | 0,023 |
| CSRP1 | 10,61 | 10,71 | 10,46 | 10,56 | 11,08 | 10,74 | 9,74 | 10,28 | 9,88 | 10,07 | 10,16 | 10,21 | -0,64 | 0,010 |
| LOC648210 | 11,33 | 11,33 | 11,46 | 10,87 | 10,85 | 10,98 | 10,41 | 10,43 | 10,55 | 10,59 | 10,18 | 10,83 | -0,64 | 0,015 |
| FLJ20699 | 6,68 | 6,73 | 6,55 | 6,41 | 6,76 | 6,59 | 6,32 | 6,01 | 6,14 | 5,74 | 5,74 | 5,95 | -0,64 | 0,007 |
| C1RL | 6,03 | 5,79 | 6,02 | 6,52 | 5,77 | 5,74 | 5,55 | 5,42 | 5,19 | 5,49 | 5,45 | 4,97 | -0,64 | 0,019 |
| FHL3 | 7,56 | 7,11 | 7,55 | 7,40 | 7,22 | 7,42 | 6,59 | 6,51 | 6,36 | 6,90 | 7,32 | 6,79 | -0,63 | 0,023 |
| HNRPA1L-2 | 10,13 | 10,43 | 10,35 | 9,71 | 9,66 | 10,11 | 9,58 | 9,14 | 9,48 | 9,47 | 9,05 | 9,86 | -0,63 | 0,035 |
| HNRPA1P4 | 10,15 | 10,52 | 10,35 | 9,88 | 9,99 | 10,21 | 9,74 | 9,31 | 9,53 | 9,68 | 9,16 | 9,89 | -0,63 | 0,019 |
| ZFYVE20 | 8,67 | 8,80 | 8,71 | 8,93 | 9,35 | 8,77 | 7,97 | 8,15 | 8,10 | 8,26 | 8,51 | 8,45 | -0,63 | 0,014 |
| LMNA | 10,96 | 10,91 | 10,96 | 10,53 | 10,45 | 10,87 | 10,22 | 9,95 | 10,29 | 10,22 | 9,66 | 10,56 | -0,63 | 0,023 |
| TOB2 | 6,18 | 5,94 | 5,91 | 6,05 | 5,85 | 6,17 | 5,07 | 5,86 | 5,68 | 5,04 | 4,95 | 5,74 | -0,63 | 0,036 |
| LOC648189 | 5,66 | 5,84 | 5,78 | 5,34 | 5,40 | 5,92 | 4,74 | 4,82 | 4,93 | 5,17 | 5,28 | 5,22 | -0,63 | 0,014 |
| SPC25 | 5,82 | 5,98 | 5,80 | 5,32 | 5,87 | 5,59 | 5,03 | 5,29 | 5,03 | 5,04 | 4,73 | 5,50 | -0,63 | 0,018 |
| NAT14 | 7,63 | 7,36 | 7,09 | 7,38 | 7,12 | 7,48 | 6,97 | 6,43 | 6,83 | 6,89 | 6,27 | 6,92 | -0,63 | 0,019 |
| ATAD3A | 7,35 | 7,40 | 7,49 | 6,88 | 6,62 | 7,04 | 6,75 | 6,57 | 6,76 | 6,33 | 5,96 | 6,66 | -0,63 | 0,045 |
| SHCBP1 | 9,96 | 10,06 | 9,93 | 9,55 | 9,88 | 9,71 | 9,03 | 8,91 | 8,99 | 9,42 | 9,26 | 9,72 | -0,63 | 0,019 |
| PKMYT1 | 5,15 | 5,58 | 5,59 | 5,00 | 5,22 | 5,17 | 4,70 | 4,47 | 4,72 | 4,66 | 4,57 | 4,83 | -0,63 | 0,009 |
| LOC727803 | 10,06 | 10,30 | 10,07 | 9,65 | 9,67 | 9,79 | 9,31 | 9,37 | 9,38 | 9,40 | 8,81 | 9,52 | -0,62 | 0,019 |
| WDR33 | 7,07 | 7,22 | 7,06 | 6,92 | 7,06 | 6,87 | 6,42 | 6,56 | 6,75 | 6,29 | 6,04 | 6,41 | -0,62 | 0,008 |
| KDELC2 | 8,55 | 8,71 | 8,64 | 8,35 | 8,31 | 8,12 | 8,15 | 7,44 | 7,98 | 7,79 | 7,46 | 8,13 | -0,62 | 0,026 |
| THOP1 | 7,19 | 7,25 | 7,31 | 6,99 | 6,80 | 7,14 | 6,43 | 6,70 | 6,88 | 6,46 | 5,90 | 6,57 | -0,62 | 0,023 |
| TPI1 | 12,00 | 11,89 | 11,86 | 12,40 | 12,39 | 12,06 | 11,60 | 11,56 | 11,26 | 11,63 | 11,44 | 11,43 | -0,62 | 0,009 |
| DSE | 7,40 | 7,22 | 7,39 | 7,41 | 7,24 | 7,31 | 7,19 | 7,03 | 6,97 | 6,52 | 6,23 | 6,33 | -0,62 | 0,032 |
| MED14 | 5,95 | 5,89 | 6,23 | 6,01 | 6,02 | 6,02 | 5,32 | 5,44 | 5,50 | 5,47 | 5,36 | 5,32 | -0,62 | 0,002 |
| ZBTB42 | 5,47 | 5,42 | 5,40 | 5,30 | 5,82 | 5,39 | 4,77 | 5,31 | 5,29 | 4,44 | 4,56 | 4,72 | -0,62 | 0,032 |
| VRK1 | 6,36 | 6,65 | 6,43 | 6,34 | 6,68 | 6,11 | 5,50 | 5,32 | 5,82 | 5,94 | 5,80 | 6,51 | -0,61 | 0,050 |
| PGM1 | 10,76 | 10,55 | 10,67 | 10,76 | 10,37 | 10,73 | 10,04 | 9,70 | 9,60 | 10,25 | 10,38 | 10,21 | -0,61 | 0,019 |
| PIF1 | 5,63 | 5,84 | 5,75 | 5,50 | 5,90 | 5,57 | 5,11 | 5,11 | 4,89 | 4,81 | 4,93 | 5,66 | -0,61 | 0,018 |
| C13orf34 | 7,40 | 7,17 | 7,25 | 6,61 | 7,07 | 6,92 | 6,59 | 6,35 | 6,75 | 6,71 | 5,78 | 6,58 | -0,61 | 0,048 |
| RUSC2 | 7,33 | 6,72 | 6,76 | 7,04 | 6,97 | 7,24 | 6,64 | 6,65 | 6,70 | 6,37 | 6,17 | 5,87 | -0,61 | 0,033 |
| NCLN | 9,12 | 9,32 | 9,43 | 8,79 | 9,30 | 9,45 | 8,74 | 9,02 | 8,86 | 8,58 | 7,95 | 8,60 | -0,61 | 0,045 |
| TMEM150A | 8,05 | 7,60 | 8,05 | 8,53 | 8,12 | 8,21 | 7,71 | 7,62 | 7,83 | 7,32 | 7,25 | 7,17 | -0,61 | 0,032 |
| TRIOBP | 6,10 | 6,78 | 6,54 | 5,91 | 6,26 | 6,35 | 5,63 | 5,92 | 5,98 | 5,51 | 5,38 | 5,84 | -0,61 | 0,029 |
| CD99L2 | 8,66 | 8,88 | 8,61 | 8,75 | 8,69 | 8,63 | 8,28 | 8,35 | 8,01 | 7,88 | 8,11 | 7,95 | -0,61 | 0,005 |
| HELLS | 5,83 | 6,19 | 5,89 | 5,78 | 5,76 | 5,63 | 5,18 | 5,40 | 5,13 | 5,64 | 4,69 | 5,39 | -0,61 | 0,025 |
| PACS1 | 6,57 | 6,07 | 6,48 | 5,92 | 6,44 | 6,38 | 5,46 | 5,83 | 5,86 | 5,38 | 5,88 | 5,79 | -0,61 | 0,018 |
| CEP78 | 5,60 | 6,34 | 6,03 | 5,58 | 6,09 | 6,01 | 5,30 | 5,27 | 5,13 | 5,72 | 5,26 | 5,35 | -0,60 | 0,022 |
| SFRS3 | 7,65 | 7,65 | 7,87 | 7,43 | 7,39 | 7,55 | 7,19 | 6,97 | 7,20 | 7,04 | 6,20 | 7,33 | -0,60 | 0,047 |
| C17orf96 | 5,25 | 5,47 | 5,11 | 5,64 | 5,59 | 5,10 | 4,96 | 4,81 | 5,10 | 4,43 | 4,71 | 4,55 | -0,60 | 0,020 |
| KANK2 | 7,47 | 7,44 | 7,50 | 7,13 | 6,95 | 7,55 | 7,07 | 6,31 | 7,09 | 6,69 | 6,76 | 6,53 | -0,60 | 0,030 |
| PFAAP5 | 6,76 | 6,57 | 6,81 | 6,56 | 6,37 | 6,66 | 6,23 | 5,94 | 5,79 | 6,10 | 5,86 | 6,21 | -0,60 | 0,008 |
| LOXL1 | 5,91 | 5,36 | 5,41 | 5,28 | 5,28 | 5,55 | 4,73 | 4,96 | 5,19 | 4,83 | 4,89 | 4,61 | -0,60 | 0,015 |
| SPOPL | 5,53 | 5,71 | 5,88 | 5,70 | 5,68 | 5,86 | 5,40 | 5,22 | 5,30 | 4,92 | 4,92 | 5,01 | -0,60 | 0,008 |
| SNRPD1 | 7,00 | 7,21 | 6,92 | 6,73 | 6,72 | 7,05 | 6,44 | 6,47 | 6,58 | 6,45 | 5,79 | 6,32 | -0,60 | 0,019 |
| RPL32 | 5,75 | 5,60 | 5,96 | 5,95 | 5,91 | 5,99 | 5,57 | 5,19 | 5,59 | 5,05 | 5,00 | 5,19 | -0,60 | 0,013 |
| KIAA0586 | 6,26 | 6,44 | 6,32 | 5,84 | 5,93 | 6,16 | 5,99 | 5,14 | 5,34 | 5,86 | 5,40 | 5,64 | -0,59 | 0,034 |
| IQCC | 6,24 | 6,44 | 6,03 | 6,23 | 5,97 | 6,32 | 5,44 | 5,37 | 5,27 | 5,90 | 5,69 | 6,02 | -0,59 | 0,022 |
| CDH11 | 11,57 | 11,78 | 11,63 | 11,78 | 11,54 | 11,67 | 11,10 | 11,38 | 11,24 | 10,98 | 10,94 | 10,78 | -0,59 | 0,008 |
| SMYD2 | 7,91 | 8,02 | 7,87 | 7,98 | 8,06 | 7,72 | 6,97 | 7,01 | 7,11 | 7,47 | 7,55 | 7,92 | -0,59 | 0,033 |
| LOC400986 | 6,42 | 6,89 | 6,57 | 6,93 | 6,63 | 6,61 | 5,91 | 5,67 | 6,04 | 6,48 | 6,12 | 6,31 | -0,59 | 0,023 |
| B4GALT4 | 6,78 | 7,08 | 7,21 | 7,27 | 7,53 | 7,31 | 6,62 | 6,97 | 6,94 | 6,31 | 6,42 | 6,42 | -0,59 | 0,030 |
| GPN1 | 8,63 | 8,56 | 8,76 | 8,31 | 8,64 | 8,59 | 7,74 | 7,76 | 8,02 | 8,38 | 7,73 | 8,36 | -0,59 | 0,021 |
| C12orf48 | 6,06 | 6,33 | 6,19 | 5,71 | 6,18 | 6,23 | 5,53 | 5,22 | 5,26 | 5,67 | 5,55 | 5,97 | -0,58 | 0,023 |
| SNHG1 | 8,38 | 8,65 | 8,19 | 8,04 | 8,18 | 8,00 | 7,56 | 7,41 | 7,72 | 7,57 | 7,72 | 7,97 | -0,58 | 0,016 |
| SNORD36A | 7,63 | 7,53 | 7,64 | 7,33 | 7,34 | 7,32 | 7,20 | 6,77 | 7,12 | 6,83 | 6,27 | 7,11 | -0,58 | 0,031 |
| LGTN | 7,51 | 7,46 | 7,34 | 7,56 | 7,04 | 7,70 | 7,10 | 6,51 | 6,72 | 7,04 | 6,58 | 7,16 | -0,58 | 0,027 |
| FAM14B | 6,15 | 5,53 | 5,80 | 6,36 | 5,96 | 6,32 | 5,28 | 5,46 | 5,61 | 5,29 | 5,35 | 5,65 | -0,58 | 0,026 |
| DEPDC7 | 5,25 | 5,87 | 5,90 | 5,36 | 5,74 | 5,95 | 4,73 | 4,96 | 4,95 | 5,19 | 5,35 | 5,39 | -0,58 | 0,036 |
| SNX26 | 6,84 | 7,11 | 7,01 | 6,24 | 6,88 | 6,92 | 5,94 | 6,08 | 6,14 | 6,45 | 6,17 | 6,74 | -0,58 | 0,046 |
| EXOSC8 | 8,30 | 8,55 | 8,33 | 8,47 | 8,22 | 8,32 | 7,55 | 7,53 | 7,86 | 8,04 | 7,69 | 8,04 | -0,58 | 0,010 |
| TTC38 | 7,05 | 7,02 | 6,94 | 6,47 | 6,85 | 7,26 | 6,44 | 6,64 | 6,17 | 6,57 | 5,87 | 6,45 | -0,58 | 0,036 |
| MTHFS | 7,45 | 7,65 | 7,18 | 7,35 | 7,17 | 7,40 | 6,89 | 7,05 | 6,61 | 6,75 | 6,53 | 6,91 | -0,58 | 0,011 |
| NT5C | 9,49 | 9,38 | 9,33 | 9,60 | 9,05 | 9,29 | 8,93 | 8,91 | 8,78 | 8,97 | 8,35 | 8,73 | -0,58 | 0,015 |
| C9orf46 | 8,83 | 8,43 | 8,41 | 8,59 | 8,16 | 8,32 | 8,14 | 7,77 | 8,05 | 7,90 | 7,51 | 7,92 | -0,58 | 0,019 |
| RANBP1 | 10,89 | 10,97 | 10,82 | 10,35 | 10,42 | 10,56 | 10,11 | 10,17 | 10,22 | 10,15 | 9,61 | 10,29 | -0,58 | 0,027 |
| RFC5 | 5,76 | 6,28 | 6,24 | 5,75 | 6,20 | 5,86 | 5,26 | 5,09 | 5,52 | 5,86 | 5,19 | 5,75 | -0,57 | 0,041 |
| BRI3BP | 5,64 | 5,67 | 5,54 | 5,43 | 5,33 | 5,28 | 4,86 | 4,93 | 4,88 | 4,93 | 4,95 | 4,90 | -0,57 | 0,004 |
| PLEKHA2 | 6,68 | 6,48 | 6,66 | 7,21 | 6,73 | 6,72 | 6,33 | 6,37 | 6,43 | 6,10 | 5,84 | 5,99 | -0,57 | 0,024 |
| GMPPB | 6,96 | 7,26 | 7,16 | 6,89 | 7,13 | 6,81 | 6,54 | 6,49 | 6,53 | 6,52 | 6,14 | 6,59 | -0,57 | 0,009 |
| CDCA4 | 6,64 | 7,32 | 6,75 | 6,67 | 6,74 | 6,53 | 5,93 | 6,01 | 6,30 | 6,37 | 6,09 | 6,55 | -0,57 | 0,030 |
| BCAS4 | 7,07 | 7,10 | 6,92 | 7,40 | 7,45 | 6,96 | 6,69 | 6,78 | 6,81 | 6,22 | 6,53 | 6,46 | -0,57 | 0,019 |
| ISOC1 | 6,68 | 6,91 | 6,46 | 7,01 | 6,87 | 6,66 | 6,10 | 6,05 | 6,40 | 6,13 | 6,19 | 6,32 | -0,57 | 0,009 |
| S100A9 | 4,92 | 5,27 | 5,13 | 5,67 | 5,50 | 5,13 | 4,56 | 4,95 | 4,58 | 4,42 | 4,82 | 4,89 | -0,57 | 0,026 |
| ERCC6L | 5,66 | 5,82 | 5,54 | 5,22 | 5,34 | 5,06 | 4,85 | 4,72 | 4,95 | 5,00 | 4,63 | 5,07 | -0,57 | 0,022 |
| DNAJC4 | 6,35 | 6,32 | 5,78 | 6,48 | 6,64 | 6,10 | 6,00 | 5,48 | 5,92 | 5,57 | 5,61 | 5,71 | -0,56 | 0,033 |
| ACD | 6,03 | 6,46 | 6,16 | 6,03 | 5,79 | 6,21 | 5,70 | 5,49 | 5,89 | 5,26 | 5,26 | 5,71 | -0,56 | 0,026 |
| LOC729366 | 6,08 | 6,04 | 6,10 | 5,76 | 5,64 | 5,93 | 5,89 | 5,31 | 5,36 | 5,23 | 4,81 | 5,58 | -0,56 | 0,047 |
| COL5A1 | 12,93 | 12,93 | 13,12 | 13,09 | 13,20 | 13,14 | 12,55 | 12,77 | 12,33 | 12,45 | 12,67 | 12,28 | -0,56 | 0,008 |
| URB2 | 5,99 | 5,83 | 5,98 | 5,91 | 6,21 | 5,96 | 5,15 | 5,31 | 5,97 | 5,36 | 5,18 | 5,54 | -0,56 | 0,023 |
| PTMS | 8,65 | 8,46 | 8,69 | 8,35 | 8,43 | 8,69 | 8,15 | 8,09 | 7,94 | 8,01 | 7,71 | 8,02 | -0,56 | 0,008 |
| ACTN1 | 11,45 | 11,27 | 11,41 | 11,23 | 11,14 | 11,49 | 10,82 | 11,18 | 10,76 | 10,87 | 10,47 | 10,53 | -0,56 | 0,016 |
| CATSPER2P1 | 5,69 | 6,08 | 5,66 | 5,30 | 5,23 | 5,41 | 4,94 | 4,85 | 5,01 | 4,79 | 5,32 | 5,13 | -0,56 | 0,035 |
| LOC728188 | 9,90 | 9,62 | 9,75 | 10,16 | 10,14 | 9,86 | 9,60 | 9,61 | 9,26 | 9,27 | 9,26 | 9,10 | -0,56 | 0,018 |
| SFRS2 | 11,03 | 11,23 | 11,19 | 10,82 | 10,84 | 11,02 | 10,67 | 10,69 | 10,80 | 10,45 | 9,85 | 10,33 | -0,56 | 0,042 |
| SLC38A2 | 10,84 | 11,20 | 11,24 | 10,72 | 11,52 | 11,20 | 10,58 | 10,55 | 10,59 | 10,63 | 10,44 | 10,59 | -0,55 | 0,018 |
| ANXA11 | 7,93 | 7,78 | 7,68 | 7,36 | 7,36 | 7,78 | 6,96 | 7,20 | 7,07 | 7,28 | 6,81 | 7,25 | -0,55 | 0,019 |
| KCNS1 | 5,18 | 5,48 | 5,13 | 5,17 | 5,17 | 4,98 | 4,52 | 4,38 | 4,80 | 4,92 | 4,66 | 4,52 | -0,55 | 0,012 |
| RNF20 | 8,54 | 8,32 | 8,29 | 8,46 | 8,19 | 8,16 | 7,74 | 7,45 | 7,47 | 8,21 | 7,78 | 8,01 | -0,55 | 0,026 |
| FUT11 | 6,39 | 6,18 | 6,23 | 6,27 | 6,16 | 5,96 | 5,60 | 5,50 | 6,13 | 5,57 | 5,47 | 5,61 | -0,55 | 0,016 |
| LOC402562 | 5,39 | 5,44 | 5,24 | 4,73 | 5,00 | 4,86 | 4,43 | 4,37 | 4,85 | 4,65 | 4,50 | 4,57 | -0,55 | 0,029 |
| CIB1 | 10,93 | 11,09 | 11,01 | 11,09 | 11,11 | 11,14 | 10,76 | 10,80 | 10,88 | 10,35 | 9,98 | 10,32 | -0,55 | 0,036 |
| CCDC8 | 5,16 | 5,50 | 4,91 | 5,02 | 4,75 | 5,01 | 4,36 | 4,29 | 4,37 | 4,72 | 4,93 | 4,41 | -0,54 | 0,035 |
| LSM8 | 6,10 | 6,16 | 5,93 | 6,21 | 5,84 | 6,14 | 5,49 | 5,28 | 5,33 | 5,63 | 5,57 | 5,83 | -0,54 | 0,012 |
| APTX | 8,25 | 8,19 | 8,25 | 8,11 | 8,00 | 8,09 | 7,68 | 7,68 | 7,51 | 7,85 | 7,29 | 7,64 | -0,54 | 0,009 |
| LOC100128936 | 11,92 | 11,82 | 11,75 | 12,04 | 11,55 | 11,84 | 11,37 | 10,90 | 11,17 | 11,38 | 11,37 | 11,51 | -0,54 | 0,016 |
| CAPG | 6,57 | 6,65 | 6,50 | 6,73 | 6,88 | 6,82 | 6,09 | 5,98 | 6,18 | 5,82 | 6,49 | 6,37 | -0,54 | 0,019 |
| H1F0 | 9,33 | 9,47 | 9,39 | 9,49 | 9,65 | 9,33 | 8,42 | 8,61 | 8,77 | 9,11 | 9,16 | 9,38 | -0,54 | 0,049 |
| LOC649445 | 5,68 | 5,89 | 6,13 | 5,43 | 5,60 | 5,86 | 5,29 | 4,92 | 5,15 | 5,35 | 5,19 | 5,48 | -0,54 | 0,024 |
| SKP1 | 7,47 | 7,26 | 7,58 | 7,53 | 7,08 | 7,52 | 7,19 | 6,95 | 7,05 | 6,73 | 6,39 | 6,92 | -0,53 | 0,032 |
| STARD7 | 8,49 | 8,51 | 8,57 | 8,06 | 8,64 | 8,42 | 7,59 | 7,82 | 7,89 | 8,03 | 7,93 | 8,24 | -0,53 | 0,021 |
| FAM54A | 5,14 | 5,69 | 5,54 | 5,33 | 5,37 | 5,25 | 4,64 | 4,69 | 4,57 | 5,10 | 4,76 | 5,37 | -0,53 | 0,044 |
| DARS | 11,64 | 11,34 | 11,72 | 11,95 | 11,34 | 11,75 | 11,32 | 10,90 | 11,15 | 11,33 | 10,78 | 11,07 | -0,53 | 0,030 |
| CECR5 | 7,27 | 7,30 | 6,91 | 7,47 | 7,46 | 7,15 | 6,67 | 6,98 | 6,85 | 6,76 | 6,60 | 6,51 | -0,53 | 0,016 |
| NRM | 6,19 | 6,28 | 5,93 | 5,83 | 5,99 | 6,43 | 5,39 | 5,49 | 5,76 | 5,69 | 5,39 | 5,73 | -0,53 | 0,019 |
| SRM | 8,66 | 8,50 | 8,52 | 8,69 | 8,81 | 8,70 | 8,18 | 8,32 | 8,54 | 7,88 | 7,74 | 8,02 | -0,53 | 0,026 |
| NTF3 | 9,23 | 8,71 | 8,84 | 9,23 | 8,83 | 9,19 | 8,33 | 8,38 | 8,55 | 8,46 | 8,37 | 8,76 | -0,53 | 0,019 |
| SAC3D1 | 7,86 | 7,90 | 7,88 | 7,63 | 7,91 | 7,63 | 7,35 | 7,31 | 7,13 | 7,33 | 6,95 | 7,57 | -0,53 | 0,014 |
| LOC137107 | 6,91 | 6,83 | 6,71 | 7,14 | 6,59 | 6,68 | 6,59 | 6,32 | 6,47 | 5,79 | 6,25 | 6,27 | -0,53 | 0,032 |
| CPNE3 | 8,05 | 8,12 | 8,27 | 8,40 | 8,70 | 8,27 | 7,57 | 7,58 | 7,82 | 7,95 | 7,93 | 7,78 | -0,53 | 0,019 |
| PRKAG1 | 7,13 | 6,86 | 6,99 | 7,13 | 6,74 | 7,01 | 6,23 | 6,23 | 6,23 | 6,47 | 6,81 | 6,74 | -0,53 | 0,025 |
| KIF26B | 5,34 | 5,12 | 5,09 | 5,36 | 5,24 | 5,39 | 4,64 | 4,79 | 5,17 | 4,50 | 4,76 | 4,56 | -0,52 | 0,018 |
| EDC3 | 5,06 | 5,54 | 5,52 | 5,45 | 5,44 | 5,30 | 4,80 | 4,67 | 5,02 | 5,00 | 5,12 | 4,58 | -0,52 | 0,019 |
| BCS1L | 6,70 | 6,96 | 6,85 | 6,47 | 6,60 | 6,63 | 6,01 | 5,88 | 6,48 | 6,09 | 6,19 | 6,43 | -0,52 | 0,023 |
| ESCO2 | 5,13 | 5,10 | 5,31 | 4,86 | 4,45 | 4,53 | 4,24 | 4,49 | 4,39 | 4,44 | 4,26 | 4,45 | -0,52 | 0,045 |
| DUS2L | 7,22 | 7,50 | 7,29 | 7,50 | 7,15 | 7,45 | 6,94 | 6,82 | 6,61 | 6,87 | 6,68 | 7,08 | -0,52 | 0,012 |
| LOC648740 | 9,03 | 8,89 | 9,00 | 8,58 | 8,57 | 8,75 | 7,94 | 8,34 | 8,48 | 8,11 | 8,28 | 8,56 | -0,52 | 0,026 |
| PLAGL2 | 5,72 | 5,29 | 5,56 | 5,72 | 5,48 | 5,57 | 5,31 | 5,27 | 5,40 | 4,74 | 4,95 | 4,59 | -0,52 | 0,048 |
| LDHA | 13,64 | 13,76 | 13,70 | 13,73 | 13,95 | 13,79 | 13,26 | 13,37 | 13,59 | 13,27 | 12,74 | 13,23 | -0,52 | 0,025 |
| RUVBL2 | 8,13 | 8,12 | 8,09 | 7,77 | 7,78 | 7,87 | 7,65 | 7,32 | 7,22 | 7,43 | 7,16 | 7,89 | -0,52 | 0,033 |
| GRPEL2 | 8,03 | 7,59 | 7,56 | 8,23 | 7,90 | 7,64 | 7,40 | 7,26 | 7,25 | 7,39 | 7,25 | 7,30 | -0,51 | 0,021 |
| IMPDH1 | 5,75 | 5,91 | 5,53 | 5,79 | 5,75 | 5,37 | 5,52 | 5,37 | 5,14 | 5,05 | 5,01 | 4,93 | -0,51 | 0,027 |
| PGAM1 | 10,48 | 10,46 | 10,40 | 10,70 | 10,77 | 10,52 | 10,04 | 10,13 | 10,05 | 10,02 | 10,04 | 9,99 | -0,51 | 0,006 |
| RNF26 | 6,92 | 7,19 | 7,02 | 6,64 | 7,22 | 7,13 | 6,44 | 6,63 | 6,47 | 6,45 | 6,37 | 6,70 | -0,51 | 0,016 |
| NUDT15 | 7,48 | 7,64 | 7,54 | 7,55 | 7,51 | 7,40 | 7,10 | 6,81 | 7,00 | 7,15 | 6,67 | 7,34 | -0,51 | 0,017 |
| CCNH | 8,07 | 7,90 | 7,89 | 8,06 | 8,27 | 8,02 | 7,25 | 7,66 | 7,67 | 7,50 | 7,40 | 7,65 | -0,51 | 0,012 |
| LIN9 | 5,63 | 5,39 | 5,51 | 5,50 | 5,42 | 5,32 | 4,61 | 4,88 | 4,76 | 4,92 | 5,04 | 5,51 | -0,51 | 0,035 |
| LOC375295 | 10,05 | 10,04 | 9,79 | 10,02 | 9,96 | 9,83 | 9,15 | 9,36 | 9,18 | 9,69 | 9,52 | 9,73 | -0,51 | 0,020 |
| FBL | 9,81 | 9,92 | 9,93 | 9,90 | 9,58 | 9,85 | 9,24 | 8,95 | 9,28 | 9,66 | 9,06 | 9,75 | -0,51 | 0,042 |
| MRPS14 | 5,99 | 5,74 | 5,71 | 5,60 | 5,39 | 5,38 | 5,38 | 4,58 | 5,21 | 5,25 | 5,20 | 5,14 | -0,51 | 0,048 |
| METTL14 | 5,34 | 5,77 | 5,51 | 5,30 | 5,46 | 5,62 | 4,59 | 4,69 | 4,94 | 5,32 | 5,19 | 5,21 | -0,51 | 0,044 |
| PGAM4 | 10,06 | 9,82 | 9,81 | 10,04 | 10,18 | 10,02 | 9,42 | 9,78 | 9,52 | 9,21 | 9,55 | 9,40 | -0,51 | 0,014 |
| PFAS | 7,90 | 8,03 | 8,00 | 7,59 | 7,52 | 7,44 | 7,32 | 6,91 | 7,30 | 7,38 | 6,97 | 7,54 | -0,51 | 0,048 |
| NUDCD3 | 7,10 | 7,02 | 7,15 | 7,17 | 6,89 | 7,36 | 6,69 | 6,56 | 6,71 | 6,45 | 6,48 | 6,76 | -0,51 | 0,010 |
| RTN4IP1 | 6,39 | 6,81 | 6,72 | 6,25 | 6,11 | 6,32 | 6,06 | 6,23 | 6,01 | 5,83 | 5,61 | 5,82 | -0,51 | 0,045 |
| BOLA3 | 10,83 | 10,68 | 10,59 | 10,58 | 10,52 | 10,55 | 10,28 | 10,11 | 10,16 | 10,18 | 9,48 | 10,50 | -0,51 | 0,049 |
| LOC727761 | 7,61 | 7,65 | 7,48 | 7,32 | 7,27 | 7,50 | 6,80 | 6,67 | 7,16 | 7,24 | 6,84 | 7,09 | -0,51 | 0,020 |
| POLG2 | 6,20 | 6,25 | 6,20 | 6,54 | 6,80 | 6,30 | 6,01 | 5,68 | 5,52 | 5,99 | 5,85 | 6,21 | -0,51 | 0,044 |
| LOC645001 | 5,21 | 5,85 | 5,41 | 5,14 | 4,97 | 5,18 | 4,93 | 4,50 | 4,85 | 4,97 | 4,81 | 4,67 | -0,50 | 0,048 |
| NR4A2 | 5,49 | 5,70 | 5,61 | 5,62 | 5,18 | 5,29 | 4,69 | 4,73 | 5,02 | 5,16 | 5,07 | 5,19 | -0,50 | 0,027 |
| LOC732007 | 10,63 | 10,52 | 10,48 | 10,69 | 10,85 | 10,62 | 10,09 | 10,28 | 10,03 | 10,10 | 10,07 | 10,19 | -0,50 | 0,007 |
| EXOSC2 | 6,98 | 7,55 | 6,98 | 6,60 | 7,13 | 6,86 | 6,62 | 6,75 | 6,56 | 6,46 | 6,33 | 6,37 | -0,50 | 0,048 |
| DAZAP1 | 7,63 | 7,50 | 7,52 | 7,51 | 7,29 | 7,56 | 7,03 | 6,68 | 6,94 | 7,28 | 6,95 | 7,10 | -0,50 | 0,014 |

| Genes upregulated in LV#18/LV#19 vs. NT/CTRL | | | | | | | | | | | | | | |
| --- | --- | --- | --- | --- | --- | --- | --- | --- | --- | --- | --- | --- | --- | --- |
| Genes | NT:19 | NT:23 | NT:27 | CTRL:19 | CTRL:23 | CTRL:27 | LV18:19 | LV18:23 | LV18:27 | LV19:19 | LV19:23 | LV19:27 | logFC | adj.P.Val |
| RGS20 | 4,81 | 5,16 | 4,66 | 5,05 | 6,17 | 4,81 | 6,77 | 7,40 | 7,64 | 6,88 | 7,55 | 7,15 | 2,12 | 0,001 |
| HIST1H4H | 6,04 | 5,26 | 5,96 | 6,86 | 7,11 | 6,02 | 8,89 | 8,69 | 8,47 | 7,29 | 7,90 | 6,69 | 1,78 | 0,015 |
| LRRC6 | 4,70 | 4,46 | 5,00 | 4,60 | 4,74 | 4,64 | 5,93 | 5,41 | 5,51 | 7,32 | 7,28 | 6,82 | 1,69 | 0,008 |
| TSPAN13 | 7,88 | 7,41 | 7,70 | 8,38 | 9,14 | 8,07 | 10,53 | 11,20 | 10,45 | 8,75 | 8,79 | 8,65 | 1,63 | 0,043 |
| AGPAT9 | 4,73 | 4,70 | 4,58 | 4,61 | 4,59 | 4,95 | 7,07 | 6,16 | 7,20 | 6,03 | 5,98 | 5,39 | 1,62 | 0,004 |
| DICER1 | 6,30 | 6,16 | 6,37 | 6,23 | 6,77 | 6,47 | 7,62 | 7,89 | 7,54 | 8,17 | 8,58 | 8,11 | 1,60 | 0,001 |
| GABBR2 | 7,03 | 6,42 | 6,66 | 5,92 | 6,41 | 6,63 | 8,14 | 8,82 | 8,96 | 7,64 | 7,27 | 7,82 | 1,60 | 0,006 |
| YPEL2 | 5,86 | 5,45 | 5,70 | 5,81 | 5,95 | 5,90 | 7,55 | 7,74 | 7,49 | 6,99 | 7,65 | 6,77 | 1,59 | 0,001 |
| STMN3 | 6,76 | 6,52 | 6,73 | 6,87 | 6,28 | 6,79 | 8,15 | 7,88 | 7,59 | 8,63 | 8,72 | 8,45 | 1,58 | 0,001 |
| HIST1H2AC | 7,62 | 7,01 | 7,27 | 8,06 | 8,27 | 7,73 | 9,24 | 9,26 | 9,06 | 9,18 | 9,93 | 8,51 | 1,54 | 0,004 |
| IRAK2 | 6,57 | 6,53 | 5,93 | 6,51 | 6,07 | 5,77 | 7,95 | 8,38 | 8,28 | 7,19 | 7,84 | 6,84 | 1,52 | 0,005 |
| GXYLT1 | 5,41 | 5,53 | 5,57 | 5,76 | 5,84 | 5,33 | 6,61 | 6,83 | 6,55 | 7,30 | 7,82 | 7,29 | 1,50 | 0,001 |
| SESN1 | 6,01 | 6,00 | 6,37 | 6,47 | 6,16 | 6,09 | 7,61 | 7,42 | 6,96 | 7,74 | 8,47 | 7,42 | 1,42 | 0,002 |
| STEAP3 | 7,08 | 7,06 | 7,19 | 6,53 | 5,76 | 6,60 | 8,99 | 8,56 | 8,69 | 7,82 | 7,16 | 7,22 | 1,37 | 0,026 |
| DUSP5 | 7,43 | 6,75 | 7,04 | 7,04 | 6,98 | 6,97 | 8,73 | 8,92 | 9,81 | 7,81 | 7,58 | 7,58 | 1,37 | 0,023 |
| HIST1H2BK | 9,57 | 9,52 | 9,56 | 10,15 | 10,61 | 9,86 | 11,38 | 11,54 | 11,21 | 10,95 | 11,53 | 10,71 | 1,34 | 0,003 |
| TP53INP1 | 5,87 | 5,64 | 6,02 | 6,23 | 6,88 | 6,15 | 8,18 | 8,41 | 7,82 | 6,67 | 7,27 | 6,48 | 1,34 | 0,023 |
| TMED5 | 8,95 | 8,20 | 8,77 | 8,64 | 8,46 | 8,01 | 9,90 | 9,96 | 10,03 | 9,71 | 9,75 | 9,55 | 1,31 | 0,001 |
| HIST1H2BD | 7,41 | 7,07 | 7,12 | 8,59 | 8,55 | 7,82 | 9,13 | 8,52 | 8,84 | 9,34 | 9,57 | 9,02 | 1,31 | 0,014 |
| FAM43A | 7,96 | 6,93 | 7,54 | 7,54 | 6,46 | 7,45 | 9,08 | 8,42 | 8,52 | 8,67 | 8,92 | 8,11 | 1,30 | 0,007 |
| LOC653879 | 5,60 | 5,15 | 4,89 | 6,11 | 4,63 | 5,56 | 7,34 | 6,90 | 6,01 | 6,83 | 7,01 | 5,44 | 1,26 | 0,029 |
| RFTN2 | 5,25 | 4,43 | 5,42 | 5,65 | 4,80 | 5,35 | 6,10 | 4,71 | 6,78 | 7,05 | 6,87 | 6,84 | 1,24 | 0,048 |
| LOC440900 | 5,80 | 5,06 | 5,55 | 5,07 | 5,31 | 5,37 | 6,75 | 7,09 | 6,94 | 6,15 | 6,30 | 6,37 | 1,24 | 0,002 |
| PSG2 | 5,87 | 5,89 | 5,30 | 4,75 | 6,34 | 5,29 | 6,87 | 7,68 | 6,52 | 6,26 | 7,09 | 6,47 | 1,24 | 0,016 |
| NAP1L5 | 7,11 | 6,74 | 6,96 | 7,58 | 7,63 | 7,24 | 8,41 | 8,41 | 8,43 | 8,51 | 8,35 | 8,55 | 1,23 | 0,001 |
| HIST2H2AA4 | 8,86 | 8,12 | 8,48 | 9,81 | 9,95 | 9,01 | 10,61 | 10,13 | 10,30 | 10,24 | 10,24 | 10,07 | 1,23 | 0,015 |
| SQRDL | 6,06 | 5,35 | 5,81 | 6,32 | 5,43 | 5,85 | 7,77 | 7,15 | 7,50 | 6,70 | 6,19 | 6,76 | 1,21 | 0,012 |
| ZNF219 | 4,93 | 4,53 | 5,12 | 5,09 | 4,64 | 4,63 | 6,08 | 6,10 | 6,19 | 5,82 | 6,16 | 5,55 | 1,16 | 0,001 |
| LY96 | 8,66 | 8,70 | 8,78 | 9,11 | 9,33 | 9,21 | 9,65 | 9,71 | 9,98 | 10,40 | 10,46 | 10,40 | 1,14 | 0,004 |
| TFPI | 6,99 | 7,42 | 7,62 | 7,93 | 8,21 | 7,41 | 7,95 | 8,99 | 8,37 | 8,62 | 9,34 | 9,11 | 1,13 | 0,014 |
| HSDL1 | 4,94 | 4,84 | 5,43 | 5,28 | 5,32 | 5,28 | 5,93 | 6,69 | 6,40 | 6,16 | 6,73 | 5,97 | 1,13 | 0,002 |
| LOC100133866 | 7,09 | 7,24 | 6,75 | 6,79 | 6,85 | 6,69 | 7,91 | 8,45 | 7,31 | 8,09 | 8,77 | 7,58 | 1,11 | 0,009 |
| AKR1B1 | 10,91 | 10,95 | 10,63 | 11,54 | 10,82 | 10,83 | 12,84 | 12,77 | 12,59 | 11,64 | 11,51 | 11,01 | 1,11 | 0,036 |
| SERPINA3 | 4,69 | 4,76 | 4,96 | 4,86 | 4,80 | 5,08 | 5,68 | 5,52 | 5,70 | 6,23 | 6,59 | 6,10 | 1,11 | 0,003 |
| QPCT | 8,50 | 7,37 | 7,90 | 9,40 | 8,01 | 8,19 | 9,96 | 9,03 | 9,50 | 9,50 | 9,09 | 8,95 | 1,11 | 0,030 |
| LOC647691 | 4,91 | 5,26 | 4,87 | 5,06 | 4,58 | 5,02 | 5,78 | 5,89 | 5,79 | 6,17 | 6,55 | 6,14 | 1,10 | 0,001 |
| GLIPR1 | 9,40 | 9,06 | 9,61 | 9,04 | 8,76 | 9,36 | 10,10 | 10,35 | 10,52 | 10,08 | 10,39 | 10,37 | 1,10 | 0,001 |
| MAPRE3 | 5,20 | 5,38 | 5,21 | 4,96 | 5,48 | 5,30 | 6,17 | 6,62 | 6,05 | 6,33 | 6,73 | 6,22 | 1,10 | 0,001 |
| HIST2H2AC | 7,80 | 7,36 | 7,59 | 8,75 | 8,82 | 7,98 | 9,58 | 9,08 | 9,27 | 9,12 | 9,04 | 8,70 | 1,08 | 0,018 |
| ZNF680 | 5,20 | 5,03 | 5,54 | 5,23 | 5,25 | 5,23 | 6,42 | 5,89 | 6,26 | 6,52 | 6,41 | 6,42 | 1,07 | 0,001 |
| C1orf218 | 6,44 | 6,31 | 6,46 | 5,69 | 5,98 | 6,17 | 6,66 | 7,03 | 6,90 | 7,89 | 7,57 | 7,43 | 1,07 | 0,008 |
| SGIP1 | 4,94 | 4,30 | 4,52 | 4,61 | 4,26 | 4,41 | 6,25 | 5,73 | 5,49 | 5,76 | 4,99 | 5,21 | 1,07 | 0,007 |
| HIST1H4E | 4,91 | 4,87 | 4,99 | 5,28 | 5,78 | 5,27 | 6,72 | 6,84 | 6,67 | 5,66 | 6,13 | 5,43 | 1,06 | 0,020 |
| SRPX2 | 8,53 | 8,19 | 8,23 | 8,99 | 8,71 | 8,46 | 9,97 | 10,03 | 9,74 | 9,32 | 9,71 | 8,60 | 1,04 | 0,014 |
| KIAA1467 | 5,66 | 4,97 | 5,25 | 4,82 | 4,52 | 4,91 | 5,89 | 6,12 | 5,64 | 6,23 | 6,29 | 6,22 | 1,04 | 0,005 |
| HIST2H2AB | 5,01 | 5,33 | 4,72 | 5,50 | 6,27 | 4,86 | 6,63 | 6,43 | 6,62 | 6,34 | 6,14 | 5,78 | 1,04 | 0,018 |
| HIST2H2AA3 | 8,67 | 7,99 | 8,03 | 9,53 | 9,76 | 8,69 | 10,05 | 9,74 | 9,81 | 10,03 | 9,53 | 9,74 | 1,04 | 0,036 |
| LOC401074 | 7,72 | 7,85 | 7,95 | 7,81 | 8,06 | 7,83 | 8,79 | 8,75 | 9,13 | 8,78 | 9,42 | 8,56 | 1,03 | 0,001 |
| DENND5B | 5,52 | 5,87 | 5,44 | 5,71 | 6,75 | 5,71 | 6,48 | 6,63 | 6,27 | 7,16 | 7,84 | 6,73 | 1,02 | 0,033 |
| IFI30 | 6,90 | 7,16 | 6,44 | 6,41 | 6,07 | 6,39 | 8,09 | 8,17 | 7,96 | 6,77 | 7,89 | 6,59 | 1,02 | 0,049 |
| NACC2 | 5,98 | 6,03 | 6,24 | 5,50 | 6,10 | 5,84 | 6,34 | 6,67 | 6,80 | 7,08 | 7,57 | 7,32 | 1,02 | 0,009 |
| CUTC | 6,77 | 7,06 | 7,04 | 6,70 | 6,60 | 7,10 | 7,41 | 7,41 | 7,25 | 8,38 | 8,57 | 8,28 | 1,01 | 0,018 |
| LAYN | 8,51 | 8,59 | 8,74 | 8,09 | 8,39 | 8,81 | 8,80 | 9,17 | 8,85 | 10,06 | 10,43 | 9,85 | 1,01 | 0,033 |
| HIST1H3D | 4,67 | 4,82 | 4,38 | 4,71 | 4,62 | 4,66 | 5,92 | 5,83 | 6,03 | 5,46 | 5,72 | 4,92 | 1,00 | 0,005 |
| C7orf41 | 7,65 | 7,59 | 7,48 | 7,77 | 7,91 | 7,83 | 8,60 | 8,35 | 8,72 | 8,70 | 9,14 | 8,74 | 1,00 | 0,001 |
| RRM2B | 6,41 | 5,96 | 6,61 | 6,62 | 6,21 | 6,55 | 7,80 | 7,18 | 7,80 | 7,33 | 6,88 | 7,32 | 0,99 | 0,006 |
| TUBA4A | 6,11 | 5,65 | 5,79 | 6,94 | 6,92 | 6,05 | 6,85 | 7,02 | 6,50 | 7,84 | 7,40 | 7,78 | 0,99 | 0,045 |
| TMEM2 | 7,91 | 7,66 | 7,62 | 7,97 | 7,69 | 7,79 | 9,20 | 9,44 | 9,07 | 8,35 | 8,25 | 8,23 | 0,99 | 0,013 |
| CAV2 | 9,17 | 9,02 | 9,22 | 9,11 | 9,30 | 9,31 | 10,14 | 10,16 | 10,32 | 10,13 | 10,14 | 10,12 | 0,98 | 0,000 |
| LOC645638 | 9,59 | 8,92 | 9,18 | 9,92 | 9,51 | 9,64 | 11,41 | 10,84 | 10,29 | 10,38 | 9,74 | 9,94 | 0,98 | 0,034 |
| C9orf5 | 7,51 | 7,74 | 7,93 | 8,33 | 9,19 | 8,16 | 8,91 | 8,99 | 8,80 | 9,17 | 9,67 | 9,16 | 0,97 | 0,027 |
| SSH2 | 5,50 | 5,79 | 5,52 | 5,20 | 5,73 | 5,41 | 6,26 | 6,53 | 6,58 | 6,53 | 6,52 | 6,52 | 0,97 | 0,001 |
| SEMA4B | 4,69 | 4,82 | 5,23 | 5,83 | 4,97 | 4,94 | 6,29 | 6,50 | 6,24 | 5,82 | 5,79 | 5,62 | 0,96 | 0,012 |
| SPP1 | 5,62 | 5,26 | 5,87 | 5,92 | 7,01 | 5,48 | 7,25 | 6,66 | 7,37 | 6,61 | 6,85 | 6,18 | 0,96 | 0,048 |
| LHFPL2 | 7,96 | 8,38 | 8,37 | 8,25 | 8,64 | 8,14 | 9,26 | 9,76 | 9,25 | 8,75 | 9,52 | 8,95 | 0,96 | 0,006 |
| FAM116A | 6,65 | 6,95 | 6,75 | 6,77 | 6,98 | 6,78 | 7,64 | 7,74 | 7,70 | 7,74 | 7,85 | 7,91 | 0,95 | 0,000 |
| BDKRB1 | 6,57 | 7,83 | 7,21 | 7,29 | 7,51 | 7,23 | 8,05 | 8,23 | 8,61 | 7,96 | 8,46 | 8,03 | 0,95 | 0,009 |
| RRAGC | 7,91 | 7,90 | 8,04 | 8,16 | 8,63 | 8,17 | 9,16 | 9,23 | 9,14 | 8,72 | 9,44 | 8,83 | 0,95 | 0,004 |
| NID2 | 7,61 | 7,71 | 7,66 | 7,97 | 7,90 | 7,73 | 8,08 | 8,54 | 8,34 | 8,85 | 9,55 | 8,91 | 0,95 | 0,013 |
| PTPRM | 7,45 | 7,40 | 7,27 | 7,18 | 7,00 | 7,35 | 7,90 | 8,00 | 7,57 | 8,63 | 9,09 | 8,15 | 0,95 | 0,016 |
| GNPDA1 | 8,77 | 8,68 | 8,75 | 9,20 | 8,99 | 8,57 | 10,15 | 9,86 | 9,84 | 9,78 | 9,42 | 9,55 | 0,94 | 0,003 |
| B3GALTL | 5,89 | 6,60 | 6,42 | 6,22 | 6,96 | 6,35 | 6,81 | 7,28 | 6,96 | 7,64 | 8,10 | 7,28 | 0,94 | 0,019 |
| AFP | 5,68 | 5,72 | 4,84 | 6,20 | 5,85 | 5,74 | 6,15 | 6,41 | 6,21 | 6,94 | 6,80 | 7,07 | 0,93 | 0,021 |
| EIF4E2 | 9,58 | 9,98 | 9,81 | 9,05 | 8,57 | 9,44 | 10,17 | 10,59 | 10,37 | 10,13 | 10,41 | 10,30 | 0,92 | 0,015 |
| SEMA3A | 5,09 | 4,47 | 4,45 | 4,55 | 4,42 | 4,39 | 5,61 | 6,65 | 5,57 | 5,23 | 4,99 | 4,86 | 0,92 | 0,041 |
| LYST | 5,07 | 5,21 | 5,66 | 4,81 | 5,04 | 4,60 | 5,97 | 6,07 | 5,96 | 5,97 | 6,09 | 5,83 | 0,92 | 0,004 |
| DNAJB9 | 7,83 | 7,29 | 7,10 | 8,03 | 8,02 | 7,52 | 8,97 | 8,91 | 8,64 | 8,30 | 8,61 | 7,85 | 0,91 | 0,019 |
| RNF182 | 5,12 | 5,20 | 5,54 | 5,04 | 5,64 | 5,61 | 6,15 | 6,11 | 7,25 | 5,94 | 5,89 | 6,28 | 0,91 | 0,019 |
| IGF2R | 9,43 | 9,54 | 9,56 | 9,88 | 10,06 | 9,67 | 11,00 | 10,99 | 10,81 | 10,23 | 10,50 | 10,07 | 0,91 | 0,009 |
| PEX11B | 8,82 | 8,67 | 8,77 | 8,13 | 7,89 | 8,29 | 9,12 | 9,00 | 8,82 | 9,61 | 9,84 | 9,61 | 0,90 | 0,019 |
| ALDH2 | 6,29 | 6,59 | 6,58 | 7,25 | 7,62 | 6,94 | 7,78 | 7,59 | 7,16 | 7,87 | 8,65 | 7,64 | 0,90 | 0,045 |
| AUH | 6,30 | 6,61 | 6,32 | 5,91 | 5,58 | 5,71 | 6,49 | 6,76 | 6,33 | 7,17 | 7,79 | 7,24 | 0,89 | 0,042 |
| APH1B | 7,54 | 7,45 | 7,29 | 8,03 | 8,13 | 7,31 | 8,81 | 8,96 | 8,51 | 8,09 | 8,65 | 8,05 | 0,89 | 0,016 |
| LOC88523 | 8,21 | 7,76 | 7,96 | 8,51 | 8,40 | 8,20 | 9,23 | 9,06 | 8,89 | 9,28 | 9,14 | 8,71 | 0,88 | 0,004 |
| ETS2 | 5,99 | 6,08 | 6,11 | 5,55 | 5,62 | 5,65 | 6,88 | 7,05 | 7,30 | 6,49 | 6,35 | 6,20 | 0,88 | 0,014 |
| TRAPPC2P1 | 6,63 | 6,90 | 6,93 | 6,82 | 6,78 | 6,96 | 7,89 | 7,65 | 7,57 | 7,57 | 7,62 | 7,96 | 0,87 | 0,001 |
| MMD | 6,41 | 6,01 | 6,29 | 6,57 | 6,40 | 6,01 | 7,61 | 7,60 | 6,90 | 7,20 | 7,07 | 6,55 | 0,87 | 0,011 |
| OBFC2A | 9,64 | 9,25 | 9,30 | 9,56 | 9,76 | 9,50 | 10,44 | 10,68 | 11,10 | 10,10 | 10,08 | 9,83 | 0,87 | 0,015 |
| CKB | 6,27 | 6,43 | 7,11 | 6,49 | 6,68 | 7,28 | 7,47 | 7,86 | 8,06 | 6,97 | 7,23 | 7,85 | 0,86 | 0,027 |
| KLHDC3 | 9,38 | 9,44 | 9,33 | 9,73 | 9,87 | 9,66 | 10,13 | 10,28 | 10,15 | 10,53 | 10,89 | 10,61 | 0,86 | 0,005 |
| ABLIM3 | 5,33 | 4,87 | 5,58 | 5,16 | 5,26 | 5,03 | 6,79 | 6,27 | 6,44 | 5,87 | 5,33 | 5,69 | 0,86 | 0,028 |
| CCDC28A | 7,70 | 7,75 | 7,67 | 7,52 | 7,01 | 7,55 | 8,28 | 8,02 | 7,50 | 8,74 | 9,10 | 8,71 | 0,86 | 0,040 |
| PPP2R5A | 5,66 | 5,48 | 5,57 | 5,07 | 4,82 | 5,37 | 5,61 | 6,33 | 6,02 | 6,52 | 6,60 | 6,05 | 0,86 | 0,014 |
| SYNJ1 | 6,66 | 6,49 | 6,59 | 6,35 | 6,41 | 6,07 | 6,72 | 7,07 | 6,90 | 7,72 | 7,67 | 7,61 | 0,85 | 0,014 |
| SPATA18 | 5,99 | 5,70 | 6,05 | 6,47 | 5,84 | 5,76 | 6,94 | 6,86 | 6,47 | 6,74 | 7,58 | 6,33 | 0,85 | 0,019 |
| DCAF6 | 7,10 | 7,15 | 7,50 | 6,91 | 7,14 | 7,21 | 7,41 | 7,59 | 7,51 | 8,37 | 8,87 | 8,35 | 0,85 | 0,038 |
| TMEM30A | 8,50 | 8,32 | 8,17 | 8,14 | 7,83 | 8,25 | 9,07 | 9,00 | 8,79 | 9,43 | 9,19 | 8,81 | 0,85 | 0,004 |
| JMJD1A | 6,97 | 6,95 | 6,99 | 6,94 | 7,16 | 6,85 | 7,95 | 7,96 | 7,27 | 7,97 | 8,05 | 7,72 | 0,84 | 0,003 |
| MRPS6 | 10,65 | 10,93 | 10,98 | 10,08 | 10,03 | 10,34 | 10,78 | 11,07 | 11,02 | 11,64 | 11,80 | 11,76 | 0,84 | 0,035 |
| MGAT4B | 6,27 | 6,10 | 6,49 | 5,41 | 5,20 | 5,53 | 6,84 | 7,09 | 6,99 | 6,26 | 6,31 | 6,55 | 0,84 | 0,042 |
| LOC647588 | 5,65 | 5,85 | 5,54 | 5,82 | 5,26 | 5,28 | 6,50 | 6,55 | 5,94 | 6,18 | 7,31 | 5,93 | 0,84 | 0,030 |
| FYN | 6,69 | 6,56 | 6,65 | 6,88 | 6,39 | 6,58 | 7,79 | 8,01 | 7,79 | 7,15 | 7,02 | 7,01 | 0,83 | 0,014 |
| CTSL1 | 11,68 | 11,99 | 11,82 | 11,88 | 11,73 | 11,81 | 13,33 | 13,25 | 12,98 | 12,22 | 12,05 | 12,06 | 0,83 | 0,040 |
| ABCC5 | 6,36 | 6,44 | 6,41 | 6,62 | 6,51 | 6,36 | 7,18 | 7,28 | 7,16 | 7,42 | 7,38 | 7,24 | 0,83 | 0,001 |
| TBX2 | 5,57 | 5,86 | 6,39 | 5,40 | 5,94 | 6,16 | 6,36 | 6,44 | 7,57 | 6,61 | 6,69 | 6,62 | 0,83 | 0,030 |
| LOC100129550 | 5,90 | 6,07 | 5,60 | 6,20 | 6,49 | 6,26 | 6,87 | 6,87 | 6,16 | 7,23 | 7,61 | 6,72 | 0,82 | 0,031 |
| LOC387763 | 6,41 | 5,76 | 6,61 | 6,92 | 6,08 | 6,74 | 7,46 | 7,42 | 7,45 | 7,12 | 6,86 | 7,15 | 0,82 | 0,018 |
| CYP4V2 | 5,84 | 6,12 | 5,95 | 6,30 | 6,67 | 5,96 | 6,41 | 7,21 | 6,19 | 7,38 | 7,62 | 6,95 | 0,82 | 0,048 |
| RRAS2 | 8,73 | 8,81 | 8,51 | 8,50 | 9,47 | 8,37 | 9,29 | 10,18 | 9,78 | 9,01 | 9,91 | 9,16 | 0,82 | 0,041 |
| SCD5 | 5,93 | 6,21 | 6,65 | 6,80 | 6,56 | 6,78 | 6,83 | 7,13 | 6,99 | 7,53 | 7,81 | 7,54 | 0,82 | 0,021 |
| LOC653610 | 5,35 | 4,58 | 5,22 | 5,14 | 5,35 | 4,70 | 6,23 | 5,94 | 6,12 | 5,94 | 5,62 | 5,38 | 0,82 | 0,014 |
| HIST1H2BC | 5,44 | 5,21 | 5,22 | 5,68 | 6,25 | 5,39 | 6,56 | 6,93 | 6,11 | 5,63 | 6,69 | 6,15 | 0,82 | 0,043 |
| GLDC | 4,44 | 4,59 | 4,31 | 4,34 | 4,63 | 4,34 | 4,92 | 5,20 | 5,94 | 4,76 | 5,37 | 5,36 | 0,82 | 0,012 |
| CDC34 | 6,69 | 6,30 | 6,61 | 7,23 | 7,16 | 6,97 | 7,84 | 7,94 | 7,68 | 7,45 | 7,52 | 7,40 | 0,81 | 0,010 |
| ZDHHC14 | 4,51 | 5,22 | 5,21 | 5,15 | 5,02 | 4,49 | 5,87 | 5,58 | 6,07 | 5,69 | 5,83 | 5,40 | 0,81 | 0,010 |
| E2F6 | 6,47 | 6,97 | 6,75 | 6,93 | 7,15 | 6,81 | 8,02 | 8,05 | 7,61 | 7,50 | 7,56 | 7,17 | 0,81 | 0,009 |
| RNF44 | 5,12 | 4,51 | 4,77 | 5,04 | 5,81 | 4,65 | 6,22 | 5,76 | 5,53 | 5,73 | 6,05 | 5,42 | 0,80 | 0,031 |
| HECA | 6,72 | 6,40 | 6,65 | 7,00 | 7,21 | 6,67 | 7,24 | 7,31 | 7,32 | 8,08 | 7,97 | 7,54 | 0,80 | 0,016 |
| ZNF91 | 6,66 | 6,57 | 6,00 | 6,57 | 7,23 | 6,21 | 6,95 | 7,69 | 6,92 | 7,48 | 7,71 | 7,30 | 0,80 | 0,030 |
| PPP2R1B | 5,02 | 5,42 | 5,20 | 5,59 | 6,15 | 5,29 | 6,16 | 6,54 | 6,27 | 6,00 | 6,66 | 5,85 | 0,80 | 0,021 |
| ZCCHC3 | 4,65 | 4,96 | 4,70 | 5,45 | 5,00 | 4,73 | 5,62 | 5,52 | 5,91 | 5,54 | 5,87 | 5,84 | 0,80 | 0,006 |
| C16orf45 | 5,84 | 5,67 | 5,82 | 5,74 | 5,07 | 5,69 | 6,60 | 5,60 | 6,07 | 6,84 | 6,77 | 6,74 | 0,80 | 0,035 |
| ABHD5 | 7,29 | 7,43 | 7,32 | 7,09 | 8,02 | 7,23 | 7,73 | 8,34 | 8,20 | 8,10 | 8,65 | 8,17 | 0,80 | 0,014 |
| TMBIM6 | 11,37 | 11,51 | 11,74 | 11,16 | 10,83 | 11,25 | 12,27 | 12,25 | 12,19 | 12,01 | 12,12 | 11,80 | 0,80 | 0,007 |
| C6orf115 | 9,46 | 8,86 | 9,05 | 9,17 | 9,03 | 8,56 | 9,75 | 9,88 | 9,64 | 9,94 | 9,61 | 10,09 | 0,80 | 0,007 |
| PBX3 | 8,30 | 8,47 | 8,33 | 8,61 | 9,32 | 8,39 | 9,21 | 9,45 | 9,48 | 9,32 | 9,47 | 9,25 | 0,79 | 0,010 |
| DTNA | 4,85 | 4,73 | 4,66 | 4,94 | 4,48 | 4,72 | 5,70 | 6,09 | 5,68 | 5,23 | 5,10 | 5,34 | 0,79 | 0,010 |
| ZNF226 | 6,67 | 6,22 | 6,69 | 6,66 | 6,34 | 6,38 | 6,89 | 6,73 | 7,27 | 7,32 | 7,80 | 7,70 | 0,79 | 0,018 |
| C6orf120 | 6,15 | 5,87 | 5,71 | 5,54 | 5,52 | 5,57 | 6,91 | 7,05 | 6,77 | 6,07 | 6,26 | 6,05 | 0,79 | 0,023 |
| HAS2AS | 5,95 | 5,70 | 5,76 | 5,83 | 5,71 | 6,00 | 6,25 | 6,60 | 6,38 | 6,68 | 6,82 | 6,95 | 0,79 | 0,004 |
| C16orf72 | 7,38 | 7,57 | 7,89 | 7,70 | 7,97 | 7,50 | 8,18 | 8,72 | 8,50 | 8,21 | 8,99 | 8,12 | 0,79 | 0,012 |
| THBD | 4,40 | 5,27 | 4,45 | 4,32 | 4,51 | 4,65 | 5,22 | 5,79 | 6,12 | 5,17 | 5,25 | 4,76 | 0,79 | 0,045 |
| SCNN1D | 5,28 | 4,74 | 5,44 | 5,71 | 5,99 | 5,52 | 6,24 | 6,21 | 6,15 | 6,40 | 6,42 | 5,98 | 0,79 | 0,016 |
| ZNF211 | 5,15 | 4,93 | 5,32 | 5,83 | 5,14 | 5,04 | 6,26 | 6,26 | 6,06 | 5,94 | 6,03 | 5,59 | 0,78 | 0,011 |
| SLC35D2 | 5,59 | 5,70 | 5,43 | 5,13 | 5,09 | 5,23 | 6,04 | 6,52 | 6,32 | 6,09 | 6,24 | 5,67 | 0,78 | 0,009 |
| RELL2 | 5,51 | 4,90 | 5,49 | 5,37 | 5,04 | 5,45 | 6,14 | 5,70 | 6,01 | 6,25 | 6,40 | 5,95 | 0,78 | 0,007 |
| POPDC3 | 5,89 | 5,76 | 6,00 | 6,37 | 6,13 | 6,00 | 6,95 | 7,02 | 7,08 | 6,62 | 6,57 | 6,58 | 0,78 | 0,005 |
| MARCH4 | 8,40 | 8,25 | 8,46 | 8,13 | 8,76 | 8,61 | 8,59 | 9,16 | 9,07 | 9,18 | 9,74 | 9,53 | 0,78 | 0,018 |
| C9orf150 | 4,90 | 4,63 | 5,51 | 5,37 | 4,79 | 5,33 | 6,39 | 5,83 | 6,44 | 5,44 | 5,25 | 5,82 | 0,78 | 0,050 |
| TP53INP2 | 5,63 | 5,53 | 5,85 | 6,08 | 6,11 | 5,66 | 6,90 | 7,08 | 6,71 | 6,27 | 6,19 | 6,37 | 0,77 | 0,015 |
| CDKN1B | 7,52 | 7,45 | 7,50 | 7,15 | 7,35 | 7,62 | 7,92 | 8,23 | 7,54 | 8,40 | 8,71 | 8,40 | 0,77 | 0,016 |
| CREBZF | 5,34 | 5,65 | 5,83 | 5,75 | 5,64 | 5,90 | 6,50 | 6,42 | 6,26 | 6,50 | 6,73 | 6,31 | 0,77 | 0,003 |
| C8orf34 | 4,48 | 5,54 | 5,55 | 5,05 | 4,82 | 5,48 | 5,57 | 6,03 | 6,27 | 5,59 | 5,96 | 6,11 | 0,77 | 0,030 |
| RFXANK | 6,58 | 6,37 | 6,40 | 6,72 | 5,92 | 6,73 | 7,33 | 7,17 | 7,23 | 7,32 | 7,24 | 7,01 | 0,77 | 0,006 |
| CTSA | 7,93 | 7,84 | 8,06 | 8,33 | 8,51 | 8,13 | 8,75 | 8,93 | 8,46 | 8,82 | 9,73 | 8,69 | 0,76 | 0,026 |
| DPY19L4 | 6,46 | 6,29 | 6,81 | 6,41 | 6,42 | 6,59 | 7,14 | 6,90 | 6,89 | 7,65 | 7,61 | 7,39 | 0,76 | 0,010 |
| HIST1H2BJ | 5,30 | 5,78 | 5,35 | 5,51 | 6,11 | 5,16 | 6,35 | 6,51 | 6,61 | 5,81 | 6,50 | 5,99 | 0,76 | 0,022 |
| CD83 | 4,68 | 4,95 | 4,75 | 4,78 | 4,40 | 4,43 | 5,40 | 5,48 | 5,31 | 5,29 | 6,00 | 5,02 | 0,75 | 0,012 |
| CAB39 | 9,03 | 9,44 | 9,25 | 9,13 | 9,71 | 9,32 | 9,74 | 10,10 | 9,68 | 10,27 | 10,49 | 10,11 | 0,75 | 0,012 |
| IDUA | 5,82 | 6,38 | 6,37 | 6,47 | 6,66 | 6,43 | 7,10 | 7,22 | 6,91 | 6,91 | 7,64 | 6,85 | 0,75 | 0,013 |
| CYP2R1 | 5,25 | 4,99 | 5,23 | 5,36 | 5,34 | 5,52 | 5,76 | 5,22 | 6,11 | 6,46 | 6,61 | 6,02 | 0,75 | 0,036 |
| LMBRD1 | 8,59 | 8,92 | 8,68 | 9,00 | 9,11 | 8,92 | 9,24 | 9,36 | 9,42 | 9,88 | 10,13 | 9,69 | 0,75 | 0,012 |
| MESDC1 | 4,60 | 5,12 | 5,09 | 4,76 | 5,29 | 4,99 | 5,85 | 6,23 | 5,80 | 5,21 | 5,64 | 5,60 | 0,75 | 0,015 |
| ZFYVE26 | 7,04 | 7,27 | 7,13 | 7,16 | 7,36 | 7,15 | 7,71 | 7,45 | 7,56 | 8,27 | 8,44 | 8,17 | 0,75 | 0,015 |
| NSMCE2 | 6,47 | 6,79 | 6,78 | 6,29 | 5,79 | 6,52 | 7,09 | 7,17 | 6,84 | 7,27 | 7,20 | 7,54 | 0,75 | 0,018 |
| HBP1 | 6,98 | 7,09 | 7,06 | 7,58 | 7,75 | 7,21 | 8,23 | 8,05 | 7,67 | 8,12 | 8,41 | 7,66 | 0,74 | 0,018 |
| GPRC5C | 4,53 | 4,69 | 4,45 | 4,90 | 5,01 | 4,43 | 5,78 | 5,69 | 5,07 | 4,90 | 5,81 | 5,22 | 0,74 | 0,022 |
| ISCU | 8,76 | 8,86 | 8,73 | 8,46 | 8,34 | 8,73 | 9,42 | 9,31 | 8,94 | 9,34 | 9,94 | 9,38 | 0,74 | 0,011 |
| ZMAT3 | 10,61 | 10,66 | 10,71 | 10,31 | 10,49 | 10,61 | 10,96 | 10,86 | 10,81 | 11,71 | 11,92 | 11,57 | 0,74 | 0,030 |
| MED28 | 7,82 | 7,68 | 7,65 | 7,98 | 8,07 | 7,71 | 8,91 | 8,73 | 8,90 | 8,27 | 8,29 | 8,26 | 0,74 | 0,009 |
| HIF1AN | 6,11 | 6,12 | 6,33 | 6,59 | 6,31 | 6,38 | 6,91 | 6,96 | 7,02 | 7,02 | 7,06 | 7,30 | 0,74 | 0,002 |
| ARMC7 | 5,12 | 5,20 | 4,95 | 4,89 | 4,98 | 5,16 | 5,78 | 5,06 | 5,32 | 6,00 | 6,49 | 6,07 | 0,74 | 0,039 |
| PCDH9 | 4,40 | 4,65 | 4,40 | 4,53 | 4,40 | 4,72 | 5,47 | 5,78 | 5,85 | 5,02 | 4,99 | 4,41 | 0,74 | 0,049 |
| WDR26 | 8,17 | 8,19 | 8,11 | 8,23 | 8,74 | 8,29 | 9,02 | 9,04 | 8,74 | 8,91 | 9,53 | 8,92 | 0,74 | 0,009 |
| ANKRD10 | 6,10 | 6,57 | 6,18 | 6,44 | 6,97 | 6,12 | 7,12 | 7,28 | 6,87 | 7,28 | 7,46 | 6,75 | 0,73 | 0,018 |
| ASB13 | 5,10 | 5,11 | 4,73 | 5,37 | 5,02 | 5,17 | 5,83 | 5,84 | 5,77 | 6,01 | 5,98 | 5,47 | 0,73 | 0,005 |
| MYL5 | 6,08 | 5,81 | 5,92 | 6,34 | 6,37 | 6,13 | 7,11 | 6,89 | 6,92 | 6,57 | 6,93 | 6,62 | 0,73 | 0,006 |
| CBLB | 8,24 | 8,57 | 7,83 | 8,31 | 8,69 | 7,96 | 8,92 | 9,53 | 9,20 | 8,78 | 9,06 | 8,47 | 0,73 | 0,030 |
| RELN | 4,36 | 4,34 | 4,55 | 4,37 | 4,48 | 4,52 | 4,64 | 5,19 | 5,14 | 5,05 | 5,61 | 5,36 | 0,73 | 0,008 |
| ADAM17 | 5,96 | 6,10 | 5,84 | 5,96 | 6,21 | 6,08 | 7,04 | 6,92 | 6,67 | 6,49 | 6,96 | 6,46 | 0,73 | 0,005 |
| ZNF702P | 4,32 | 4,32 | 4,39 | 4,54 | 4,38 | 4,31 | 4,67 | 5,36 | 4,65 | 5,04 | 6,07 | 4,85 | 0,73 | 0,046 |
| TP53BP1 | 7,35 | 7,50 | 7,49 | 7,55 | 7,83 | 7,53 | 8,14 | 7,96 | 8,15 | 8,35 | 8,67 | 8,30 | 0,72 | 0,006 |
| YPEL5 | 8,57 | 8,53 | 8,43 | 9,07 | 9,19 | 8,94 | 9,11 | 9,48 | 9,03 | 9,70 | 10,08 | 9,62 | 0,71 | 0,036 |
| VPS26 | 7,79 | 8,35 | 8,17 | 8,15 | 8,23 | 8,26 | 8,94 | 8,88 | 9,21 | 8,62 | 8,71 | 8,81 | 0,71 | 0,006 |
| MAP3K11 | 5,16 | 5,43 | 5,54 | 5,05 | 5,72 | 5,33 | 6,26 | 6,61 | 6,38 | 5,46 | 5,88 | 5,86 | 0,70 | 0,033 |
| LPHN1 | 4,41 | 4,91 | 4,53 | 4,61 | 4,99 | 4,72 | 4,86 | 5,75 | 5,10 | 5,42 | 6,00 | 5,25 | 0,70 | 0,031 |
| TNFRSF10D | 5,92 | 6,21 | 6,28 | 6,33 | 6,79 | 6,28 | 6,67 | 7,35 | 7,17 | 6,66 | 7,32 | 6,84 | 0,70 | 0,020 |
| FBXO22 | 6,05 | 6,20 | 6,22 | 6,02 | 6,02 | 6,05 | 7,08 | 7,02 | 6,96 | 6,46 | 6,78 | 6,46 | 0,70 | 0,006 |
| TDG | 7,83 | 7,67 | 7,67 | 7,99 | 8,10 | 7,83 | 8,22 | 8,48 | 8,30 | 8,55 | 9,03 | 8,71 | 0,70 | 0,010 |
| C2CD2 | 7,36 | 7,54 | 7,45 | 7,71 | 7,72 | 7,34 | 8,40 | 8,39 | 8,98 | 7,74 | 7,94 | 7,87 | 0,70 | 0,036 |
| LOC100133443 | 4,98 | 5,11 | 5,14 | 4,73 | 5,07 | 5,03 | 5,26 | 6,11 | 5,07 | 6,03 | 6,04 | 5,75 | 0,70 | 0,030 |
| LOC641825 | 4,94 | 5,32 | 4,90 | 5,20 | 5,36 | 4,73 | 5,54 | 6,24 | 5,66 | 5,72 | 6,02 | 5,46 | 0,70 | 0,016 |
| NPC1 | 7,51 | 8,10 | 7,89 | 7,63 | 8,59 | 7,85 | 8,55 | 8,90 | 8,77 | 8,42 | 8,79 | 8,32 | 0,70 | 0,026 |
| HDHD2 | 7,79 | 8,01 | 7,83 | 8,10 | 8,19 | 8,12 | 8,74 | 8,62 | 8,31 | 8,86 | 9,01 | 8,67 | 0,70 | 0,006 |
| ACVR1 | 9,64 | 9,50 | 9,65 | 9,63 | 9,72 | 9,51 | 10,50 | 10,60 | 10,42 | 10,14 | 10,30 | 9,86 | 0,70 | 0,006 |
| LOC646463 | 6,48 | 6,95 | 6,09 | 6,63 | 7,19 | 6,83 | 7,50 | 7,90 | 7,33 | 7,00 | 7,60 | 7,01 | 0,69 | 0,046 |
| SMPDL3A | 6,49 | 6,53 | 6,18 | 6,51 | 6,79 | 6,43 | 7,09 | 7,68 | 7,01 | 6,94 | 7,57 | 6,81 | 0,69 | 0,019 |
| IGF2BP2 | 9,79 | 9,19 | 9,66 | 9,75 | 9,48 | 9,56 | 10,54 | 10,78 | 10,39 | 10,01 | 9,96 | 9,89 | 0,69 | 0,022 |
| CD58 | 6,96 | 7,01 | 6,83 | 6,74 | 6,62 | 6,92 | 6,83 | 7,65 | 7,25 | 7,43 | 8,29 | 7,74 | 0,69 | 0,045 |
| MTF2 | 7,14 | 7,09 | 6,80 | 6,65 | 7,08 | 6,66 | 7,40 | 7,51 | 7,34 | 7,73 | 8,11 | 7,43 | 0,68 | 0,014 |
| RNF38 | 6,05 | 5,60 | 5,83 | 6,02 | 6,60 | 6,11 | 6,71 | 6,84 | 6,55 | 6,69 | 6,78 | 6,75 | 0,68 | 0,012 |
| SESTD1 | 7,11 | 7,14 | 7,14 | 7,29 | 7,45 | 7,16 | 7,64 | 8,02 | 8,00 | 7,84 | 8,22 | 7,67 | 0,68 | 0,005 |
| STX6 | 7,28 | 6,92 | 7,20 | 7,04 | 6,43 | 6,82 | 7,51 | 7,69 | 7,70 | 7,66 | 7,54 | 7,69 | 0,68 | 0,009 |
| KAT2B | 6,57 | 6,72 | 6,91 | 6,23 | 6,79 | 7,04 | 7,44 | 7,45 | 7,61 | 7,17 | 7,34 | 7,32 | 0,68 | 0,009 |
| HADH2 | 6,86 | 6,74 | 6,57 | 6,76 | 6,58 | 6,96 | 7,30 | 7,62 | 7,45 | 7,21 | 7,59 | 7,37 | 0,68 | 0,004 |
| WASF1 | 4,47 | 4,96 | 4,69 | 4,86 | 4,80 | 4,67 | 4,98 | 5,48 | 5,07 | 5,34 | 6,09 | 5,59 | 0,68 | 0,026 |
| CXorf39 | 5,48 | 5,49 | 5,29 | 4,91 | 5,18 | 5,48 | 5,90 | 6,09 | 5,88 | 5,94 | 6,14 | 5,94 | 0,68 | 0,005 |
| NECAP1 | 7,84 | 8,00 | 7,95 | 7,94 | 7,87 | 7,78 | 8,67 | 8,53 | 8,46 | 8,56 | 8,66 | 8,56 | 0,68 | 0,001 |
| EFR3A | 8,87 | 8,85 | 8,86 | 8,95 | 8,93 | 9,04 | 9,51 | 9,41 | 9,30 | 9,93 | 9,98 | 9,42 | 0,68 | 0,008 |
| APLP2 | 7,29 | 7,25 | 7,33 | 7,36 | 7,39 | 7,30 | 8,01 | 8,02 | 7,71 | 8,05 | 8,36 | 7,82 | 0,67 | 0,004 |
| OGFRL1 | 5,89 | 5,19 | 5,55 | 6,09 | 5,77 | 5,60 | 6,32 | 6,76 | 6,64 | 6,10 | 6,28 | 6,01 | 0,67 | 0,026 |
| SLC38A6 | 7,13 | 6,84 | 7,05 | 7,26 | 6,93 | 7,16 | 7,58 | 7,45 | 7,40 | 8,11 | 7,91 | 7,93 | 0,67 | 0,011 |
| IFNGR1 | 8,60 | 8,47 | 8,64 | 8,92 | 8,49 | 8,73 | 9,80 | 9,82 | 9,44 | 9,08 | 9,10 | 8,61 | 0,67 | 0,046 |
| VPS33A | 5,61 | 5,66 | 5,81 | 6,04 | 5,98 | 5,98 | 6,42 | 6,78 | 6,42 | 6,25 | 6,81 | 6,39 | 0,66 | 0,008 |
| NR2F1 | 5,26 | 5,42 | 5,47 | 5,63 | 5,61 | 5,21 | 5,98 | 6,42 | 5,96 | 5,79 | 6,44 | 6,00 | 0,66 | 0,010 |
| SPIRE1 | 8,60 | 8,64 | 8,96 | 8,70 | 8,68 | 8,88 | 9,43 | 9,52 | 9,57 | 9,17 | 9,49 | 9,26 | 0,66 | 0,004 |
| SNX24 | 6,79 | 6,35 | 6,45 | 7,04 | 7,31 | 6,74 | 7,33 | 7,56 | 7,11 | 7,44 | 7,81 | 7,40 | 0,66 | 0,028 |
| TMEM57 | 5,22 | 5,44 | 5,42 | 5,57 | 5,68 | 5,56 | 5,81 | 6,35 | 6,20 | 6,06 | 6,56 | 5,87 | 0,66 | 0,012 |
| ATP6V1G1 | 11,02 | 10,72 | 10,72 | 11,32 | 11,47 | 11,03 | 11,88 | 11,70 | 11,80 | 11,57 | 11,69 | 11,57 | 0,66 | 0,012 |
| TMEM8 | 6,90 | 7,04 | 7,32 | 7,48 | 7,72 | 7,54 | 7,91 | 8,09 | 7,91 | 7,77 | 8,35 | 7,93 | 0,66 | 0,018 |
| KLHL5 | 6,67 | 6,94 | 6,68 | 6,51 | 6,52 | 6,55 | 7,10 | 7,75 | 7,02 | 7,25 | 7,74 | 6,95 | 0,66 | 0,020 |
| ST6GALNAC4 | 6,19 | 6,66 | 6,46 | 6,47 | 6,73 | 6,26 | 7,05 | 7,61 | 7,53 | 6,69 | 7,15 | 6,66 | 0,65 | 0,036 |
| PHLPP2 | 5,67 | 5,82 | 5,66 | 4,74 | 5,45 | 5,72 | 5,85 | 6,39 | 6,22 | 6,12 | 6,16 | 6,22 | 0,65 | 0,030 |
| SAMD14 | 5,09 | 4,87 | 5,25 | 5,91 | 5,66 | 5,07 | 6,32 | 6,14 | 5,87 | 5,86 | 5,85 | 5,72 | 0,65 | 0,037 |
| G3BP2 | 8,51 | 8,41 | 8,50 | 7,99 | 8,24 | 8,27 | 8,92 | 8,97 | 8,69 | 9,17 | 8,95 | 9,11 | 0,65 | 0,007 |
| SNRK | 6,52 | 6,36 | 6,70 | 6,25 | 6,18 | 6,29 | 6,94 | 7,18 | 7,33 | 6,89 | 7,03 | 6,80 | 0,64 | 0,008 |
| ZNF605 | 5,24 | 5,54 | 5,26 | 5,60 | 5,94 | 5,89 | 6,32 | 6,36 | 6,36 | 6,22 | 6,07 | 6,00 | 0,64 | 0,014 |
| GATS | 5,22 | 5,14 | 5,21 | 5,57 | 4,99 | 5,52 | 6,07 | 5,74 | 5,54 | 5,99 | 6,30 | 5,86 | 0,64 | 0,015 |
| HECW2 | 5,63 | 5,72 | 5,85 | 5,48 | 5,11 | 5,36 | 6,17 | 6,18 | 6,18 | 6,22 | 6,11 | 6,13 | 0,64 | 0,007 |
| BRP44 | 8,86 | 9,26 | 8,97 | 8,58 | 9,19 | 9,15 | 9,37 | 9,74 | 9,51 | 9,49 | 9,98 | 9,78 | 0,64 | 0,014 |
| CPEB2 | 5,81 | 5,63 | 5,93 | 5,53 | 5,74 | 5,93 | 6,53 | 6,34 | 7,11 | 6,31 | 6,12 | 6,01 | 0,64 | 0,029 |
| MAP1A | 7,81 | 7,48 | 7,84 | 7,73 | 8,07 | 8,19 | 8,35 | 8,60 | 8,34 | 8,58 | 8,52 | 8,59 | 0,64 | 0,008 |
| ZCCHC6 | 5,17 | 5,11 | 5,03 | 5,04 | 4,70 | 5,22 | 5,64 | 5,79 | 5,91 | 5,43 | 5,86 | 5,50 | 0,64 | 0,008 |
| EML1 | 6,99 | 6,36 | 7,11 | 6,91 | 6,54 | 6,94 | 7,91 | 7,31 | 7,67 | 7,24 | 7,22 | 7,36 | 0,64 | 0,025 |
| PDHX | 7,30 | 7,13 | 7,29 | 7,65 | 7,71 | 7,17 | 8,22 | 7,98 | 8,01 | 7,88 | 8,07 | 7,96 | 0,64 | 0,008 |
| CCDC23 | 8,14 | 8,18 | 8,14 | 8,21 | 8,05 | 8,20 | 8,61 | 8,47 | 8,33 | 9,09 | 9,20 | 9,06 | 0,64 | 0,019 |
| LOC732360 | 5,24 | 5,37 | 5,54 | 5,89 | 5,62 | 5,39 | 5,95 | 6,20 | 6,03 | 6,04 | 6,46 | 6,21 | 0,64 | 0,010 |
| C14orf159 | 6,62 | 6,83 | 6,82 | 6,75 | 6,54 | 6,51 | 7,48 | 7,30 | 6,76 | 7,70 | 7,53 | 7,13 | 0,64 | 0,019 |
| DOCK10 | 9,32 | 9,18 | 9,24 | 9,26 | 8,92 | 9,01 | 9,50 | 9,37 | 9,99 | 10,11 | 9,91 | 9,88 | 0,64 | 0,014 |
| SORCS2 | 4,48 | 4,52 | 4,61 | 4,56 | 4,93 | 4,63 | 5,48 | 4,97 | 5,63 | 5,06 | 5,57 | 4,83 | 0,64 | 0,021 |
| PBX2 | 5,48 | 5,40 | 5,94 | 6,27 | 5,49 | 5,78 | 6,51 | 6,25 | 6,41 | 6,39 | 6,08 | 6,55 | 0,64 | 0,021 |
| PDZD8 | 5,72 | 5,92 | 5,88 | 5,17 | 5,37 | 5,34 | 5,96 | 6,26 | 6,22 | 6,29 | 6,51 | 5,97 | 0,63 | 0,021 |
| C3 | 4,76 | 4,53 | 4,42 | 4,90 | 4,70 | 4,38 | 5,54 | 5,23 | 5,04 | 5,23 | 5,48 | 4,99 | 0,63 | 0,011 |
| DAXX | 6,08 | 5,89 | 6,07 | 6,15 | 6,12 | 5,93 | 6,81 | 6,60 | 6,65 | 6,39 | 7,10 | 6,49 | 0,63 | 0,008 |
| RNF135 | 4,74 | 5,24 | 5,23 | 4,93 | 4,77 | 4,51 | 5,33 | 5,48 | 5,29 | 5,76 | 6,04 | 5,33 | 0,63 | 0,030 |
| PSTPIP2 | 6,15 | 6,00 | 5,62 | 6,41 | 5,89 | 5,76 | 6,82 | 6,62 | 6,46 | 6,46 | 6,68 | 6,58 | 0,63 | 0,012 |
| SHB | 5,79 | 5,69 | 5,84 | 6,03 | 5,98 | 6,26 | 6,69 | 6,96 | 6,75 | 6,29 | 6,43 | 6,27 | 0,63 | 0,016 |
| SECISBP2 | 6,27 | 6,41 | 6,45 | 6,36 | 6,17 | 6,19 | 7,22 | 7,38 | 6,89 | 6,78 | 6,82 | 6,54 | 0,63 | 0,014 |
| NEDD4 | 6,05 | 5,71 | 5,72 | 5,92 | 6,27 | 5,74 | 7,03 | 6,85 | 6,80 | 6,22 | 6,23 | 6,06 | 0,63 | 0,046 |
| ERCC2 | 6,85 | 6,49 | 6,62 | 6,21 | 6,17 | 6,61 | 6,91 | 7,05 | 7,02 | 7,29 | 7,34 | 7,11 | 0,63 | 0,012 |
| PCMTD2 | 6,15 | 6,08 | 6,44 | 6,45 | 5,92 | 6,38 | 6,84 | 6,40 | 6,75 | 7,07 | 7,23 | 6,90 | 0,63 | 0,019 |
| PPP2R2A | 8,14 | 7,95 | 7,93 | 8,06 | 8,31 | 7,99 | 8,73 | 9,04 | 9,08 | 8,48 | 8,42 | 8,39 | 0,63 | 0,016 |
| N4BP2L1 | 4,47 | 4,29 | 4,40 | 4,43 | 4,33 | 4,26 | 4,77 | 4,50 | 4,58 | 5,24 | 5,61 | 5,24 | 0,63 | 0,041 |
| NOMO1 | 7,24 | 7,11 | 7,51 | 7,79 | 7,28 | 7,64 | 8,12 | 7,88 | 7,98 | 8,06 | 8,28 | 8,00 | 0,63 | 0,011 |
| ENTPD4 | 6,33 | 5,76 | 6,27 | 5,97 | 6,16 | 6,33 | 7,21 | 6,73 | 6,97 | 6,49 | 6,50 | 6,69 | 0,63 | 0,019 |
| LOC100130886 | 6,89 | 7,17 | 7,06 | 7,23 | 7,62 | 7,33 | 7,92 | 8,25 | 7,74 | 7,75 | 7,74 | 7,65 | 0,62 | 0,015 |
| HSD17B10 | 7,11 | 6,94 | 7,12 | 6,95 | 7,07 | 7,24 | 7,85 | 7,73 | 7,84 | 7,39 | 7,99 | 7,38 | 0,62 | 0,009 |
| CYB561D1 | 6,64 | 6,77 | 6,73 | 6,97 | 7,07 | 6,94 | 7,10 | 7,20 | 7,21 | 7,70 | 7,93 | 7,72 | 0,62 | 0,024 |
| ARG2 | 5,28 | 4,73 | 4,47 | 4,47 | 4,57 | 4,80 | 5,03 | 5,73 | 5,33 | 5,26 | 5,42 | 5,30 | 0,62 | 0,023 |
| BIRC2 | 8,09 | 7,77 | 8,10 | 7,83 | 8,54 | 8,27 | 8,68 | 8,95 | 9,21 | 8,48 | 8,47 | 8,52 | 0,62 | 0,033 |
| ATP6V0E1 | 11,70 | 12,03 | 11,80 | 11,41 | 12,15 | 11,74 | 12,29 | 12,85 | 12,52 | 12,24 | 12,41 | 12,21 | 0,62 | 0,019 |
| LOC100133211 | 7,42 | 7,03 | 7,01 | 7,42 | 7,52 | 7,35 | 7,78 | 8,30 | 8,20 | 7,69 | 7,85 | 7,63 | 0,62 | 0,019 |
| SLC35A2 | 5,99 | 6,38 | 6,21 | 6,32 | 6,14 | 6,49 | 6,58 | 6,77 | 6,55 | 6,77 | 7,19 | 7,34 | 0,61 | 0,023 |
| FBXO46 | 5,38 | 5,45 | 5,25 | 5,20 | 5,04 | 5,64 | 5,59 | 5,77 | 5,46 | 6,20 | 6,39 | 6,21 | 0,61 | 0,041 |
| C20orf111 | 8,53 | 8,25 | 8,52 | 8,53 | 8,69 | 8,72 | 9,13 | 9,03 | 8,92 | 9,23 | 9,40 | 9,20 | 0,61 | 0,007 |
| TAP1 | 8,45 | 8,42 | 8,32 | 8,79 | 8,41 | 8,25 | 9,17 | 8,92 | 8,52 | 9,24 | 9,68 | 8,77 | 0,61 | 0,046 |
| FAM103A1 | 7,37 | 7,25 | 7,27 | 7,42 | 7,32 | 7,33 | 7,91 | 7,94 | 7,93 | 7,95 | 7,77 | 8,11 | 0,61 | 0,002 |
| TSPAN6 | 6,69 | 6,50 | 6,49 | 6,16 | 5,91 | 5,95 | 6,86 | 7,01 | 7,12 | 6,99 | 6,60 | 6,79 | 0,61 | 0,024 |
| LOC100129104 | 4,98 | 5,31 | 5,22 | 5,16 | 5,46 | 4,59 | 5,91 | 5,93 | 6,13 | 5,72 | 5,28 | 5,38 | 0,61 | 0,048 |
| LOC388681 | 5,69 | 5,74 | 5,48 | 5,69 | 5,49 | 5,24 | 6,14 | 6,63 | 5,63 | 6,11 | 6,42 | 6,04 | 0,61 | 0,029 |
| C7orf43 | 6,22 | 5,88 | 5,85 | 5,78 | 6,40 | 6,29 | 6,74 | 7,03 | 6,94 | 6,54 | 6,17 | 6,62 | 0,61 | 0,033 |
| CAPN5 | 6,81 | 7,14 | 7,50 | 7,06 | 6,88 | 7,20 | 7,72 | 7,41 | 7,51 | 7,96 | 7,88 | 7,74 | 0,61 | 0,016 |
| USP24 | 7,47 | 7,24 | 7,41 | 7,32 | 7,50 | 7,38 | 8,19 | 7,96 | 7,76 | 8,02 | 8,12 | 7,93 | 0,61 | 0,004 |
| DSCR3 | 7,74 | 7,79 | 7,67 | 8,02 | 8,44 | 7,89 | 8,12 | 8,28 | 8,38 | 8,68 | 9,06 | 8,63 | 0,60 | 0,046 |
| RABL4 | 5,83 | 5,84 | 5,72 | 5,74 | 5,45 | 6,25 | 6,54 | 6,59 | 6,53 | 6,15 | 6,33 | 6,29 | 0,60 | 0,014 |
| AFG3L1 | 5,87 | 5,68 | 5,53 | 5,46 | 5,87 | 5,44 | 6,70 | 6,48 | 6,33 | 5,97 | 6,19 | 5,79 | 0,60 | 0,030 |
| C9orf6 | 6,95 | 6,70 | 6,54 | 7,11 | 7,11 | 6,76 | 7,57 | 7,55 | 7,31 | 7,38 | 7,81 | 7,15 | 0,60 | 0,017 |
| SERP2 | 7,12 | 7,02 | 6,84 | 7,53 | 7,46 | 7,07 | 7,71 | 7,66 | 7,94 | 7,69 | 7,88 | 7,75 | 0,60 | 0,012 |
| TNRC6B | 6,65 | 7,08 | 7,11 | 7,23 | 7,03 | 6,93 | 7,97 | 7,73 | 7,87 | 7,36 | 7,05 | 7,63 | 0,60 | 0,033 |
| CCDC55 | 6,69 | 6,59 | 6,53 | 6,53 | 6,03 | 6,21 | 7,02 | 7,05 | 7,05 | 6,90 | 7,22 | 6,91 | 0,60 | 0,011 |
| ZNF75A | 6,51 | 6,35 | 6,86 | 6,61 | 6,67 | 6,63 | 7,39 | 7,34 | 7,15 | 7,22 | 7,01 | 7,09 | 0,60 | 0,007 |
| SNX4 | 7,53 | 7,66 | 7,79 | 8,03 | 8,55 | 7,87 | 8,29 | 8,52 | 8,45 | 8,47 | 8,88 | 8,36 | 0,59 | 0,038 |
| FAM190B | 6,60 | 7,14 | 6,83 | 6,81 | 7,13 | 6,98 | 7,41 | 7,31 | 7,33 | 7,60 | 8,14 | 7,28 | 0,59 | 0,032 |
| NOTCH3 | 6,45 | 6,46 | 7,12 | 6,94 | 6,97 | 6,83 | 7,32 | 7,24 | 7,74 | 7,21 | 7,45 | 7,38 | 0,59 | 0,020 |
| C17orf91 | 9,21 | 8,72 | 8,89 | 8,96 | 8,67 | 8,95 | 10,03 | 9,64 | 9,73 | 9,28 | 9,25 | 9,03 | 0,59 | 0,041 |
| RIT1 | 6,59 | 6,72 | 6,89 | 6,94 | 7,15 | 7,02 | 7,30 | 7,55 | 7,45 | 7,27 | 7,83 | 7,46 | 0,59 | 0,012 |
| PTPN4 | 6,21 | 5,89 | 6,04 | 6,00 | 6,14 | 6,17 | 6,63 | 6,80 | 6,34 | 6,53 | 7,14 | 6,56 | 0,59 | 0,014 |
| LMBR1 | 7,05 | 6,99 | 7,44 | 7,24 | 6,77 | 7,12 | 7,53 | 7,40 | 7,63 | 7,93 | 8,11 | 7,52 | 0,59 | 0,025 |
| DPH3 | 7,55 | 7,43 | 7,22 | 7,64 | 7,87 | 7,38 | 8,12 | 8,45 | 8,30 | 7,96 | 7,58 | 8,19 | 0,59 | 0,031 |
| RTN4 | 11,32 | 11,35 | 11,76 | 11,30 | 11,80 | 11,65 | 12,08 | 12,14 | 12,21 | 11,96 | 12,24 | 12,05 | 0,59 | 0,009 |
| SDCBP | 9,03 | 8,92 | 9,09 | 9,07 | 9,33 | 9,09 | 9,76 | 9,63 | 10,01 | 9,45 | 9,55 | 9,62 | 0,58 | 0,008 |
| LOC641989 | 4,55 | 4,47 | 4,63 | 4,61 | 4,59 | 4,37 | 5,15 | 5,55 | 5,01 | 5,45 | 4,82 | 4,75 | 0,58 | 0,022 |
| CCDC90B | 9,35 | 9,15 | 9,29 | 8,89 | 8,61 | 8,76 | 9,65 | 9,69 | 9,49 | 9,41 | 9,79 | 9,53 | 0,58 | 0,020 |
| ARMCX1 | 8,54 | 8,10 | 8,39 | 8,62 | 8,60 | 8,54 | 9,02 | 8,50 | 8,97 | 9,20 | 9,32 | 9,27 | 0,58 | 0,026 |
| LOC645969 | 6,68 | 6,77 | 6,86 | 6,54 | 6,81 | 6,78 | 7,08 | 7,43 | 7,33 | 7,17 | 7,51 | 7,41 | 0,58 | 0,006 |
| CCDC28B | 6,19 | 6,05 | 5,98 | 6,29 | 5,84 | 6,20 | 6,80 | 6,76 | 6,42 | 6,59 | 6,80 | 6,67 | 0,58 | 0,007 |
| MPP5 | 6,69 | 6,58 | 6,85 | 6,87 | 7,28 | 6,92 | 7,23 | 7,52 | 7,57 | 7,30 | 7,99 | 7,06 | 0,58 | 0,041 |
| PVRL2 | 5,43 | 5,46 | 5,27 | 5,85 | 5,72 | 5,17 | 6,29 | 6,42 | 5,84 | 5,65 | 6,27 | 5,91 | 0,58 | 0,039 |
| EIF3J | 7,25 | 7,39 | 7,08 | 6,91 | 6,74 | 7,02 | 7,63 | 8,05 | 7,86 | 7,36 | 7,62 | 7,34 | 0,58 | 0,028 |
| LYRM2 | 7,99 | 8,16 | 8,02 | 7,55 | 7,81 | 7,92 | 8,63 | 8,60 | 8,45 | 8,56 | 8,16 | 8,51 | 0,58 | 0,012 |
| C10orf104 | 5,53 | 5,31 | 5,09 | 5,70 | 5,60 | 5,22 | 5,91 | 5,92 | 5,95 | 5,97 | 6,32 | 5,83 | 0,58 | 0,014 |
| UBE2H | 6,63 | 6,72 | 6,60 | 6,96 | 7,09 | 6,69 | 7,30 | 7,77 | 7,27 | 7,24 | 7,53 | 7,02 | 0,58 | 0,020 |
| ARHGAP12 | 6,48 | 6,37 | 6,42 | 6,58 | 6,51 | 6,40 | 6,73 | 6,76 | 6,90 | 7,15 | 7,61 | 7,06 | 0,58 | 0,022 |
| UBTD2 | 6,36 | 6,39 | 6,56 | 5,87 | 5,93 | 6,37 | 6,57 | 6,54 | 6,55 | 7,16 | 7,24 | 6,87 | 0,58 | 0,049 |
| LOC286208 | 5,11 | 5,16 | 4,65 | 5,42 | 5,47 | 5,24 | 5,84 | 6,10 | 5,91 | 5,47 | 5,57 | 5,59 | 0,57 | 0,033 |
| STK35 | 5,82 | 5,90 | 5,80 | 5,57 | 6,03 | 5,66 | 6,37 | 6,20 | 6,45 | 6,43 | 6,43 | 6,34 | 0,57 | 0,005 |
| C1orf52 | 8,64 | 8,09 | 8,43 | 8,63 | 8,54 | 8,58 | 9,16 | 9,20 | 8,95 | 8,99 | 9,01 | 9,04 | 0,57 | 0,008 |
| TACC2 | 4,78 | 5,39 | 5,01 | 5,08 | 5,37 | 5,28 | 5,74 | 5,85 | 5,69 | 5,33 | 6,19 | 5,54 | 0,57 | 0,032 |
| C6orf106 | 5,39 | 5,19 | 5,74 | 5,12 | 5,24 | 5,81 | 5,88 | 6,07 | 6,08 | 5,68 | 6,16 | 6,04 | 0,57 | 0,024 |
| LYPLA2P1 | 5,75 | 5,63 | 5,05 | 5,33 | 5,81 | 5,24 | 6,25 | 5,89 | 5,81 | 5,92 | 6,23 | 6,14 | 0,57 | 0,029 |
| NRP1 | 8,64 | 8,88 | 8,69 | 8,40 | 8,62 | 8,41 | 9,48 | 9,28 | 9,47 | 8,96 | 9,07 | 8,79 | 0,57 | 0,022 |
| C3orf63 | 4,87 | 5,51 | 5,32 | 5,29 | 5,17 | 4,90 | 5,84 | 5,95 | 5,93 | 5,40 | 5,85 | 5,51 | 0,57 | 0,024 |
| ATP6V1B2 | 9,33 | 9,48 | 9,46 | 9,42 | 9,84 | 9,51 | 10,17 | 10,29 | 10,32 | 9,90 | 10,02 | 9,76 | 0,57 | 0,014 |
| LPIN1 | 8,26 | 8,23 | 7,97 | 8,50 | 8,57 | 8,07 | 8,50 | 8,89 | 8,74 | 8,84 | 9,40 | 8,63 | 0,57 | 0,039 |
| HIST1H2AD | 4,88 | 4,59 | 4,64 | 4,73 | 4,71 | 4,85 | 5,44 | 5,14 | 5,17 | 5,10 | 5,89 | 5,07 | 0,57 | 0,024 |
| CCDC127 | 5,94 | 6,11 | 6,32 | 6,35 | 6,57 | 6,51 | 6,53 | 6,61 | 6,65 | 7,06 | 7,33 | 7,00 | 0,56 | 0,043 |
| BIRC3 | 7,03 | 7,19 | 7,14 | 7,08 | 7,46 | 7,17 | 7,51 | 8,24 | 7,96 | 7,60 | 7,67 | 7,45 | 0,56 | 0,026 |
| NRBP1 | 6,10 | 6,57 | 6,35 | 5,83 | 6,23 | 6,38 | 6,53 | 6,86 | 6,76 | 6,78 | 7,31 | 6,59 | 0,56 | 0,036 |
| C9orf123 | 5,74 | 5,65 | 5,72 | 6,21 | 6,30 | 5,81 | 6,70 | 6,63 | 6,67 | 6,32 | 6,51 | 5,96 | 0,56 | 0,044 |
| LOC390298 | 7,10 | 6,80 | 6,81 | 6,78 | 6,84 | 7,04 | 7,66 | 7,59 | 7,63 | 7,16 | 7,22 | 7,49 | 0,56 | 0,012 |
| MRPL44 | 7,24 | 7,19 | 7,32 | 7,09 | 7,45 | 7,36 | 7,52 | 7,95 | 7,79 | 7,80 | 8,00 | 7,94 | 0,56 | 0,008 |
| KLHL28 | 6,96 | 6,69 | 6,60 | 7,10 | 7,25 | 6,86 | 7,13 | 7,33 | 7,22 | 7,67 | 7,95 | 7,49 | 0,56 | 0,043 |
| LOC100128353 | 9,98 | 10,12 | 10,05 | 9,93 | 9,98 | 9,96 | 10,36 | 10,54 | 10,58 | 10,47 | 10,74 | 10,67 | 0,56 | 0,004 |
| FLJ43663 | 4,47 | 4,63 | 5,00 | 4,54 | 4,52 | 4,46 | 4,86 | 5,00 | 5,31 | 5,42 | 5,42 | 4,94 | 0,56 | 0,023 |
| KIAA1128 | 6,66 | 6,97 | 6,65 | 6,48 | 6,87 | 6,98 | 7,13 | 7,18 | 7,14 | 7,40 | 7,94 | 7,14 | 0,56 | 0,038 |
| PAIP2 | 8,08 | 7,71 | 7,66 | 7,59 | 7,81 | 7,78 | 8,43 | 8,69 | 8,35 | 8,17 | 8,22 | 8,10 | 0,55 | 0,014 |
| LEPROTL1 | 8,00 | 8,35 | 8,09 | 8,42 | 8,90 | 8,29 | 8,66 | 8,91 | 8,70 | 8,96 | 9,30 | 8,84 | 0,55 | 0,045 |
| C4orf32 | 6,32 | 5,85 | 6,07 | 6,01 | 6,33 | 5,93 | 6,48 | 6,96 | 6,87 | 6,33 | 6,67 | 6,50 | 0,55 | 0,021 |
| WDR81 | 6,34 | 6,01 | 6,12 | 6,29 | 5,82 | 6,07 | 6,97 | 6,93 | 6,87 | 6,20 | 6,44 | 6,54 | 0,55 | 0,036 |
| VPS26A | 9,60 | 9,64 | 9,53 | 9,55 | 9,57 | 9,58 | 10,28 | 10,23 | 10,25 | 10,05 | 10,01 | 9,95 | 0,55 | 0,004 |
| VRK3 | 5,22 | 5,07 | 4,75 | 5,13 | 5,43 | 4,95 | 6,13 | 5,67 | 5,59 | 5,27 | 5,84 | 5,32 | 0,55 | 0,049 |
| STK10 | 6,62 | 6,46 | 6,30 | 6,06 | 6,52 | 6,45 | 6,87 | 6,99 | 6,98 | 6,77 | 7,19 | 6,88 | 0,55 | 0,012 |
| PHACTR2 | 8,09 | 8,04 | 8,00 | 7,53 | 7,91 | 7,71 | 8,20 | 8,33 | 8,14 | 8,64 | 8,74 | 8,51 | 0,55 | 0,025 |
| ABCF3 | 5,21 | 4,90 | 4,91 | 5,15 | 5,03 | 4,65 | 5,58 | 5,47 | 5,60 | 5,51 | 5,77 | 5,19 | 0,54 | 0,016 |
| MTHFD2L | 5,32 | 5,45 | 5,00 | 5,41 | 5,82 | 5,28 | 6,17 | 6,13 | 6,07 | 5,82 | 5,69 | 5,65 | 0,54 | 0,034 |
| PHTF1 | 7,24 | 7,25 | 7,14 | 7,38 | 7,26 | 7,13 | 7,69 | 7,49 | 7,70 | 8,02 | 8,05 | 7,71 | 0,54 | 0,011 |
| WASF3 | 7,48 | 7,42 | 7,51 | 7,70 | 7,53 | 7,52 | 8,37 | 8,28 | 7,85 | 8,13 | 8,16 | 7,60 | 0,54 | 0,020 |
| LOC100132048 | 4,52 | 4,67 | 4,83 | 4,84 | 4,73 | 4,73 | 4,88 | 5,37 | 4,93 | 5,21 | 5,75 | 5,41 | 0,54 | 0,033 |
| SAP30L | 9,01 | 9,09 | 8,83 | 8,88 | 9,20 | 8,86 | 9,41 | 9,58 | 9,29 | 9,53 | 9,73 | 9,56 | 0,54 | 0,009 |
| MEG3 | 9,32 | 8,82 | 9,39 | 9,55 | 9,59 | 9,42 | 9,95 | 9,90 | 10,07 | 9,75 | 10,13 | 9,53 | 0,54 | 0,035 |
| FLJ38482 | 6,34 | 6,26 | 5,97 | 6,09 | 6,23 | 6,11 | 6,62 | 6,45 | 6,32 | 6,82 | 7,09 | 6,94 | 0,54 | 0,027 |
| LOC100133398 | 5,95 | 5,93 | 5,94 | 5,32 | 5,45 | 5,75 | 6,12 | 6,40 | 6,27 | 6,05 | 6,16 | 6,55 | 0,53 | 0,030 |
| NOMO2 | 10,10 | 9,71 | 10,15 | 10,26 | 10,14 | 10,22 | 10,63 | 10,48 | 10,61 | 10,82 | 10,65 | 10,58 | 0,53 | 0,011 |
| SLC35B3 | 7,72 | 7,91 | 7,69 | 7,88 | 7,58 | 7,56 | 8,21 | 8,30 | 8,23 | 8,16 | 8,53 | 8,11 | 0,53 | 0,009 |
| ZNF439 | 6,25 | 5,54 | 5,99 | 6,33 | 6,26 | 6,37 | 6,56 | 6,72 | 6,54 | 6,75 | 6,60 | 6,77 | 0,53 | 0,030 |
| RFP | 5,83 | 5,84 | 5,52 | 6,28 | 6,02 | 5,71 | 6,49 | 6,19 | 6,61 | 6,31 | 6,36 | 6,45 | 0,53 | 0,021 |
| FBXO33 | 8,00 | 8,19 | 8,27 | 8,19 | 8,53 | 8,63 | 8,72 | 9,07 | 9,17 | 8,67 | 8,68 | 8,70 | 0,53 | 0,028 |
| LOC728537 | 4,78 | 5,04 | 4,59 | 5,12 | 5,02 | 4,60 | 5,23 | 5,35 | 5,30 | 5,84 | 5,49 | 5,12 | 0,53 | 0,034 |
| UBE4B | 7,44 | 7,63 | 7,64 | 7,19 | 7,13 | 7,32 | 7,74 | 7,53 | 7,77 | 8,14 | 8,24 | 8,12 | 0,53 | 0,037 |
| GPR177 | 6,79 | 6,85 | 7,09 | 6,76 | 6,40 | 6,82 | 7,12 | 7,36 | 7,69 | 7,01 | 7,47 | 7,26 | 0,53 | 0,030 |
| PSAP | 11,80 | 11,74 | 11,79 | 12,37 | 12,25 | 12,06 | 12,72 | 12,51 | 12,39 | 12,51 | 12,79 | 12,25 | 0,53 | 0,031 |
| SAPS3 | 6,19 | 6,60 | 6,33 | 5,95 | 6,29 | 6,23 | 6,54 | 6,86 | 6,49 | 6,96 | 7,01 | 6,91 | 0,53 | 0,024 |
| LRP10 | 10,81 | 10,47 | 10,54 | 10,75 | 10,84 | 10,67 | 11,16 | 11,22 | 11,12 | 11,05 | 11,48 | 11,22 | 0,53 | 0,009 |
| TUSC3 | 7,52 | 7,79 | 7,57 | 8,08 | 8,37 | 7,88 | 8,35 | 8,56 | 8,72 | 8,20 | 8,27 | 8,27 | 0,53 | 0,048 |
| TAOK1 | 5,80 | 6,06 | 5,84 | 5,46 | 5,69 | 5,88 | 6,30 | 6,65 | 6,58 | 6,24 | 6,13 | 5,97 | 0,53 | 0,031 |
| LOC644517 | 6,74 | 6,74 | 6,96 | 6,85 | 6,66 | 6,98 | 7,28 | 7,62 | 7,27 | 7,04 | 7,53 | 7,35 | 0,53 | 0,013 |
| SERF1B | 7,94 | 7,59 | 8,18 | 7,90 | 7,66 | 7,94 | 8,36 | 8,11 | 8,26 | 8,69 | 8,22 | 8,69 | 0,52 | 0,031 |
| STK39 | 8,75 | 8,66 | 8,81 | 8,65 | 9,04 | 8,80 | 9,07 | 9,35 | 9,41 | 9,31 | 9,32 | 9,40 | 0,52 | 0,008 |
| ROD1 | 9,13 | 8,71 | 9,03 | 9,28 | 9,38 | 9,22 | 9,68 | 9,84 | 9,80 | 9,55 | 9,51 | 9,50 | 0,52 | 0,019 |
| CYB5R4 | 6,64 | 6,50 | 6,48 | 6,27 | 6,69 | 6,18 | 7,09 | 7,03 | 7,15 | 7,07 | 6,74 | 6,82 | 0,52 | 0,016 |
| ANKRD28 | 5,45 | 5,85 | 5,95 | 5,74 | 5,87 | 5,80 | 6,08 | 6,30 | 6,06 | 6,41 | 6,63 | 6,32 | 0,52 | 0,019 |
| LOC645659 | 4,80 | 4,69 | 4,65 | 4,75 | 5,16 | 4,93 | 5,65 | 5,22 | 5,01 | 5,48 | 5,60 | 5,13 | 0,52 | 0,031 |
| RNASEK | 10,40 | 10,53 | 10,42 | 10,71 | 10,74 | 10,46 | 11,03 | 11,27 | 11,06 | 10,89 | 11,24 | 10,87 | 0,52 | 0,012 |
| SERPINB8 | 6,71 | 6,40 | 6,71 | 6,49 | 6,50 | 6,51 | 6,82 | 6,99 | 7,60 | 6,90 | 7,03 | 7,10 | 0,52 | 0,026 |
| KIAA0895L | 4,44 | 4,82 | 4,55 | 4,56 | 4,36 | 5,02 | 4,74 | 4,95 | 5,13 | 5,14 | 5,46 | 5,42 | 0,52 | 0,049 |
| HEATR5B | 6,20 | 6,43 | 6,20 | 6,33 | 6,36 | 6,22 | 6,49 | 6,81 | 6,82 | 6,88 | 7,03 | 6,81 | 0,52 | 0,009 |
| C20orf3 | 8,55 | 8,25 | 8,37 | 7,92 | 7,83 | 8,10 | 8,88 | 8,80 | 8,80 | 8,62 | 8,46 | 8,55 | 0,52 | 0,030 |
| FAM45A | 9,62 | 9,54 | 9,31 | 9,91 | 9,96 | 9,63 | 10,07 | 10,44 | 9,94 | 10,15 | 10,50 | 9,96 | 0,52 | 0,038 |
| COG1 | 6,12 | 6,16 | 5,76 | 6,09 | 6,05 | 5,92 | 6,64 | 6,66 | 6,58 | 6,21 | 6,87 | 6,24 | 0,52 | 0,025 |
| KIAA0174 | 9,50 | 9,59 | 9,60 | 9,92 | 9,76 | 9,64 | 10,07 | 10,21 | 9,95 | 10,36 | 10,40 | 10,12 | 0,52 | 0,012 |
| DNAJB6 | 9,25 | 9,37 | 9,24 | 9,34 | 9,84 | 9,27 | 9,66 | 10,28 | 9,98 | 9,66 | 10,06 | 9,75 | 0,51 | 0,037 |
| MGC71993 | 10,11 | 10,08 | 10,11 | 10,37 | 10,55 | 10,34 | 10,73 | 10,97 | 10,82 | 10,56 | 10,92 | 10,64 | 0,51 | 0,015 |
| ZNF256 | 5,29 | 5,59 | 5,27 | 5,75 | 5,59 | 5,53 | 6,05 | 6,04 | 5,90 | 6,24 | 6,04 | 5,81 | 0,51 | 0,014 |
| CLDN1 | 4,26 | 4,37 | 4,20 | 4,37 | 4,33 | 4,40 | 5,00 | 4,98 | 4,59 | 4,84 | 5,03 | 4,56 | 0,51 | 0,012 |
| PHLDA3 | 7,13 | 7,00 | 7,23 | 7,34 | 7,42 | 7,30 | 7,84 | 7,99 | 7,95 | 7,48 | 7,75 | 7,46 | 0,51 | 0,020 |
| TMEM55B | 5,70 | 6,22 | 6,05 | 6,18 | 6,37 | 6,06 | 6,78 | 6,57 | 6,75 | 6,34 | 6,73 | 6,46 | 0,51 | 0,023 |
| GOLGA4 | 5,73 | 6,22 | 6,12 | 5,56 | 5,76 | 5,64 | 6,34 | 6,45 | 6,48 | 6,48 | 6,45 | 5,87 | 0,51 | 0,049 |
| GLB1 | 9,38 | 9,35 | 9,42 | 9,75 | 9,62 | 9,59 | 10,00 | 9,81 | 9,72 | 10,23 | 10,55 | 9,84 | 0,51 | 0,046 |
| LOC728006 | 8,02 | 7,82 | 8,08 | 8,21 | 7,62 | 7,81 | 8,61 | 8,15 | 8,51 | 8,64 | 8,26 | 8,43 | 0,51 | 0,024 |
| DPY19L1 | 8,27 | 8,56 | 8,48 | 8,53 | 8,86 | 8,49 | 9,21 | 9,31 | 8,93 | 9,08 | 9,00 | 8,69 | 0,51 | 0,024 |
| CIDECP | 5,54 | 5,63 | 5,45 | 5,75 | 5,70 | 5,76 | 6,14 | 6,47 | 5,93 | 5,86 | 6,38 | 6,10 | 0,51 | 0,021 |
| GPX1 | 11,20 | 11,15 | 10,88 | 11,04 | 10,76 | 10,83 | 11,65 | 11,96 | 11,50 | 11,19 | 11,39 | 11,20 | 0,51 | 0,043 |
| MTMR14 | 5,76 | 5,73 | 5,38 | 5,73 | 5,49 | 5,96 | 6,06 | 6,11 | 5,84 | 6,28 | 6,51 | 6,29 | 0,50 | 0,031 |
| TULP3 | 5,86 | 5,68 | 5,79 | 5,10 | 5,45 | 5,43 | 6,21 | 5,84 | 6,04 | 5,87 | 6,22 | 6,16 | 0,50 | 0,037 |
| MAPK13 | 8,47 | 8,63 | 8,10 | 8,76 | 8,81 | 8,32 | 9,02 | 9,02 | 9,28 | 8,98 | 9,05 | 8,77 | 0,50 | 0,034 |
| KIAA1737 | 6,39 | 6,01 | 6,02 | 5,89 | 5,87 | 5,63 | 6,60 | 6,54 | 6,40 | 6,33 | 6,48 | 6,47 | 0,50 | 0,020 |
| ATP6AP1 | 10,69 | 10,78 | 10,87 | 10,76 | 10,55 | 10,81 | 11,29 | 11,41 | 10,91 | 11,32 | 11,37 | 11,16 | 0,50 | 0,012 |
| PPM1D | 6,69 | 6,50 | 6,59 | 6,73 | 6,71 | 6,74 | 7,53 | 7,55 | 7,19 | 6,93 | 7,04 | 6,74 | 0,50 | 0,043 |
| METTL9 | 5,11 | 5,21 | 5,17 | 5,32 | 5,28 | 5,29 | 5,82 | 5,80 | 5,85 | 6,00 | 5,57 | 5,34 | 0,50 | 0,017 |
